# Control of Virulence by Small RNAs in *Streptococcus pneumoniae*

**DOI:** 10.1371/journal.ppat.1002788

**Published:** 2012-07-12

**Authors:** Beth Mann, Tim van Opijnen, Jianmin Wang, Caroline Obert, Yong-Dong Wang, Robert Carter, Daniel J. McGoldrick, Granger Ridout, Andrew Camilli, Elaine I. Tuomanen, Jason W. Rosch

**Affiliations:** 1 Department of Infectious Diseases, St. Jude Children's Research Hospital, Memphis, Tennessee, United States of America; 2 Department of Molecular Biology and Microbiology, Tufts University School of Medicine, Boston, Massachusetts, United States of America; 3 Hartwell Center for Bioinformatics and Biotechnology, St. Jude Children's Research Hospital, Memphis, Tennessee, United States of America; 4 Howard Hughes Medical Institute and Tufts University School of Medicine, Department of Molecular Biology and Microbiology, Boston, Massachusetts, United States of America; Institut Pasteur, France

## Abstract

Small noncoding RNAs (sRNAs) play important roles in gene regulation in both prokaryotes and eukaryotes. Thus far, no sRNA has been assigned a definitive role in virulence in the major human pathogen *Streptococcus pneumoniae*. Based on the potential coding capacity of intergenic regions, we hypothesized that the pneumococcus produces many sRNAs and that they would play an important role in pathogenesis. We describe the application of whole-genome transcriptional sequencing to systematically identify the sRNAs of *Streptococcus pneumoniae*. Using this approach, we have identified 89 putative sRNAs, 56 of which are newly identified. Furthermore, using targeted genetic approaches and Tn-seq transposon screening, we demonstrate that many of the identified sRNAs have important global and niche-specific roles in virulence. These data constitute the most comprehensive analysis of pneumococcal sRNAs and provide the first evidence of the extensive roles of sRNAs in pneumococcal pathogenesis.

## Author Summary

Pneumonia is a leading cause of childhood mortality worldwide, resulting in more deaths in young children than any other infectious disease. One of the leading causes of pneumonia is the human pathogen, *Streptococcus pneumoniae*, the causative agent of over six million infections each year in the United States. Understanding how bacterial pathogens rapidly respond to dynamic host environments is a central aspect of microbial pathogenesis. Accumulating evidence has implicated sRNAs as vital regulators in a number of important cellular processes though few have been implicated in virulence. In our investigations we have applied next-generation sequencing to define the sRNA repertoire of *S. pneumoniae*. In addition, we utilized both targeted genetic knockouts and transposon mutagenesis to show that a significant portion of these sRNAs play important roles at various stages of pneumococcal pathogenesis. These data represent the first example of sRNAs being involved in pneumococcal pathogenesis and greatly expand the number of sRNAs that play important roles in bacterial pathogenesis.

## Introduction

Gene regulation and intercellular communication are fundamental aspects of bacterial adaptation to dynamic environments. As such, bacteria have evolved numerous strategies to facilitate tight control of genetic networks in response to diverse extracellular stimuli. Roles have been described for DNA, RNA and protein in gene regulation. Only recently have we begun to appreciate the global roles of sRNAs, particularly in regards to bacterial pathogenesis, as the traditional genetic screens for virulence factors have typically not focused on these small, rarely annotated sRNAs. In recent years there has been a constantly expanding repertoire of sRNAs being identified in a number of bacterial pathogens using both tiling arrays as well as high-throughput sequencing of RNA (RNA-seq). Bioinformatic approaches have also predicted numerous sRNAs in many bacterial pathogens indicating a high prevalence of sRNAs encoded by diverse bacterial species [1], [2]. The increasingly important role of sRNAs in controlling gene expression in bacteria suggests a subset of these molecules may have roles in bacterial virulence [3], [4].

One of the more compelling cases for the role of sRNAs in bacterial pathogenesis arose from studies of Hfq, a chaperone providing stability to sRNA, which substantially advanced our knowledge of the diversity and functional roles of sRNAs in bacteria [5]. Homologs of Hfq are found in diverse species of Gram-negative and Gram-positive bacterial pathogens [6]. Deleting Hfq, which has pleiotropic effects on the stability of several sRNAs, predictably results in numerous phenotypes, mainly consisting of resistance to various environmental stresses, suggesting potential roles in host pathogenesis [6], [7], [8]. There are also numerous examples of sRNAs that function independently of Hfq, even in bacterial species that encode the chaperone. While deletion of Hfq in *Listeria* has a discernable effect on virulence, its absence does not affect the level of expression of sRNAs [7], [9]. Additionally, deletion of Hfq in *S. aureus* was found to have no detectable effect on the microbial stress response nor the function of sRNAs [10]. Despite the apparent absence of Hfq, pathogenic streptococci nonetheless encode and express an abundance of sRNAs [11], [12], [13]. In *S. pyogenes*, the regulatory RNAs RivX and FasX have been implicated in virulence gene regulation and interactions with host cells, respectively [14], [15], [16], [17]. Additionally, a specific sRNA, tracrRNA, serves a central function in the CRISPR system that mediates the silencing of foreign nucleic acid sequences [18]. Regulatory RNAs targeting virulence gene expression in streptococci function both at the transcriptional and translational levels [19]. The interactions of sRNAs are complex, with examples of the same sRNA functioning to both activate and repress target genes by a number of mechanisms [20]. Despite the increase in our knowledge of sRNAs, their contribution to virulence has been much less well established though examples have been demonstrated [3], [21], [22]. In *S. pyogenes*, deletion of the 4.5S RNA component of the signal recognition particle pathway results in significant attenuation of tissue disease [23]. *S. aureus* encodes numerous sRNAs, of which the best characterized example is RNAIII, which coordinates the expression of virulence genes [24], [25], [26], [27], [28]. Examination of the transcriptome of *L. monocytogenes* indicated the presence of several sRNAs implicated in pathogenesis that were not found in closely related non-pathogenic species [9], [29]. Recent reports have also shown sRNAs being involved in pathogenesis in *Salmonella* and *Yersinia* [30], [31]. Despite these examples, the contribution of the vast majority of sRNAs to bacterial pathogenesis, particularly in *Streptococcus pneumoniae*, remains uncharacterized.

*S. pneumoniae* is a leading cause of childhood mortality worldwide and is a major health concern despite widespread vaccination. The pneumococcus is remarkably adept at colonizing and infecting diverse niches in the human body, readily establishing itself as a commensal in the nasopharynx in over 40% of healthy individuals as well as being a major causative agent of pneumonia, otitis media, sepsis, and meningitis [32], [33]. A number of well characterized virulence genes have tissue-restricted virulence phenotypes, underscoring the diverse pneumococcal arsenal for targeting dissimilar host tissues [34], [35]. One major facet of gene regulation is the set of 13 two-component systems (TCSs) encoded in the pneumococcal genome that control a multitude of gene networks and are implicated in pathogenesis [36]. Included in these networks are sRNAs, some of which are controlled by the CiaR response regulator in the pneumococcus [37]. This phenomenon is not restricted to pneumococci, as other streptococcal species harboring CiaR also are predicted to encode numerous sRNAs, indicating that downstream sRNAs may be an important facet of regulation by this TCS [38]. Of the sRNAs identified thus far in the pneumococcus, none have been found to play a definitive role in the regulation of virulence genes or networks.

A substantial number of sRNAs have been predicted in the sequenced pneumococcal reference strains D39 and TIGR4 using bioinformatics, tiling arrays, and sequencing [11], [12], [39]. However, none have been assigned a role in host pathogenesis. To address this possibility, we undertook a sequencing based approach to identify sRNAs in pneumococcus coupled with both targeted and random gene deletions to ascertain the impact of sRNAs on pneumococcal disease. We present data identifying sRNAs in the pneumococcus by RNA sequencing (RNA-seq). Furthermore, using both transposon mutagenesis (Tn-seq) and targeted deletions, we describe data indicating that many sRNAs play vital roles in progression of infection with unique sRNAs being required for specific tissue tropism. These data provide the first comprehensive analysis of the contribution of sRNAs to pneumococcal pathogenesis and greatly expand the repertoire of sRNAs that play definitive roles in bacterial virulence.

## Results

### Isolation and Identification of sRNAs

**Figure 1:**
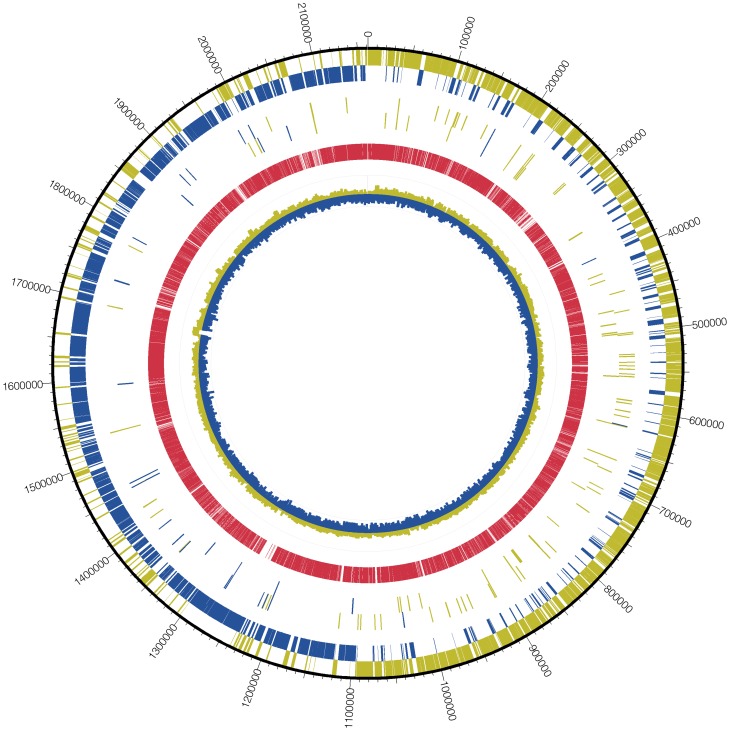
Transcriptome map of *S. pneumoniae* TIGR4. Compilation of the sRNA sequences yields a comprehensive transcriptome map of TIGR4. The outermost circle represents the chromosomal sequence of TIGR4 along with genomic coordinates. The sense and antisense transcripts are indicated by yellow and blue bars, respectively. Going inwards, the next pair of circles represents the position and orientation of the sRNAs identified in our study in addition to those predicted from previous reports. The red circle represents the total coverage of RNA based upon sequencing. The innermost circle indicates 60 bp windows of below average GC content (blue) and above average GC content (yellow).

To initially identify sRNAs, we isolated, enriched, and fully sequenced small (<200 nt) transcripts of the TIGR4 strain of pneumococcus. To broaden sRNA capture, we also analyzed mutants in genes encoding the response regulator of three two-component systems (TCS): GRR (TCS03), CbpR (TCS06), and VncR (TCS10) - all of which influence the expression of many transcripts in pneumococcus [40], [41]. TCSs monitor environmental cues to precisely control networks of gene expression; elimination of TCS control could potentially broaden total transcript abundance and thereby capture sRNAs that would otherwise be overlooked. In addition, TCSs have been shown to control the expression of sRNAs both in Gram-negative and Gram-positive bacteria, both as positive and negative regulators [37], [42]. The TCS mutants and TIGR4 were sequenced individually and the data were pooled to generate the composite of sRNAs. For each strain analyzed, coverage exceeded 99.9% with a read depth ranging from 100–400 providing high confidence in sequence quality. The data were next processed to eliminate all sequences within known ORFs to focus on intergenic regions or those running antisense to known ORFs as well as further constraints as detailed in the methods. The position of the identified sRNAs both from our analysis and previous reports were mapped to the TIGR4 genome. The sRNAs were found to be more abundant on the positive strand, though numerous sequences were identified on the negative strand (Figure 1).

**Figure 2:**
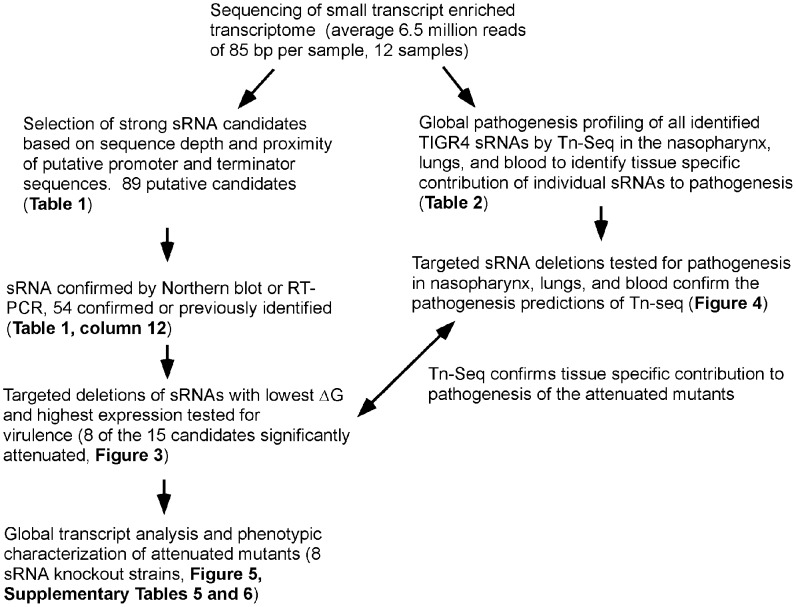
Flowchart of the order and types of analyses for identification and characterization of sRNAs. The order of the experiments performed, along with the corresponding figures and tables is outlined.

We identified 89 putative sRNAs (Table 1). Of these, 56 were novel and the rest have been recently identified by various studies (Table 1, column 11). By BLAST analysis, 85 sRNAs were highly conserved (>90%) amongst pneumococci, 11 were conserved amongst streptococci, and 17 were conserved amongst other Gram-positive bacteria, typically other lactic acid bacteria. Figure 2 outlines the order of analyses applied to the identified sRNAs. Of the 89 sRNAs identified by sequencing, 41 were confirmed for expression and size via Northern blot analysis (Figure S1 in Text S1), an additional 4 were confirmed by qRT-PCR analysis (Table 1), and 10 sRNAs were confirmed by previous studies. Seventeen of the novel sRNAs contained a highly conserved BOX element, making specific detection by Northern blotting or qRT-PCR difficult as the BOX element encompassed a majority of the predicted sRNA sequence in many instances. RNA-seq of the TCS knockouts allowed for the identification of additional sRNAs that were not expressed in the parental TIGR4. An example is shown in Figure S2 in Text S1; the F13 sRNA had high expression in the TCS knockout while being undetectable in the parental TIGR4. In total, there were 24 sRNA candidates that failed to meet the cutoff criteria in all three TIGR4 RNA-seq assemblies but were present in at least one of the TCS knockouts. These data indicate the pneumococcus expresses numerous, highly conserved sRNAs.

**Table 1:** sRNA identification and characteristics.

					Flanking Genes								
ID	Start	End	Size (nt)	Strand	Up	Down	Orientation	ΔG	Conservation	Previously identified	Confirmation	Motif/Family	Virulence
R1	171483	171628	146	−	SP0178	SP0179	< > >	−46.9	α γ	SN4 (a), srn029 (d)	Northern		
R2	1221797	1221902	106	−	SP1286	SP1287	> < <	−23.1	α		No Signal	Motif 2	
R3	1275652	1275704	53	−	SP1355	SP1356	< < <	−5.4	α β		No, BOX element		
R4	1351009	1351076	68	−	SP1431	SP1432	> < >	−6.6	α		No Signal		Blood
R6	1731557	1731734	178	−	SP1820	SP1821	< < <	−42.37	α	srn400 (d)	Northern	T-box	
R7	1791010	1791080	71	−	SP1886	SP1887	> < <	−13	α		Northern	Motif 4	
R8	1892645	1892717	73	−	SP1988	SP1989	< < <	−11	α		Northern		Nasopharynx,Blood
R9	1903548	1903638	91	−	SP1999	SP2000	< < <	−19.5	α	trn0935 (d)	Northern		
R10	1987487	1987533	47	−	SP2076	SP2077	< < <	−7.1	α β γ		Northern		
R11	2002164	2002195	32	−	SP2093	SP2094	> < <	−2.1	α γ		Northern		
R12	1731041	1731440	400	−	SP1818	SP1819	> < <	−111.39	α	trn0830 (d)	Northern	Motif 1 & 5	Nasopharynx, Blood
R13	623327	623372	46	−	SP0649	SP0650	< < >	−8.7	α γ		No Signal		
R14	1034021	1034100	80	−	SP1100	SP1101	> < <	−9.8	α		qRT-PCR		Lung
R15	1214248	1214371	124	−	SP1278	SP1279	< < <	−38.9	α	SN42 (a), srn299 (d)	Previous Study	Pyr	
R16	1227703	1227744	42	−	SP1292	SP1293	> < <	−3.9	α γ		No Signal		Nasopharynx
R17	1277341	1277388	48	−	SP1356	SP1357	< < <	−3.7	α β		No Signal		
R18	1364575	1364706	132	−	SP1444	SP1445	> < <	−25.5	α		Northern		
R19	1390953	1391074	122	−	SP1477	SP1478	< < <	−23.1	α β	trn0663 (d)	Northern		
R20	1455141	1455185	45	−	SP1547	SP1548	< < <	−12.2	α γ		Northern		
R21	1461116	1461178	63	−	SP1551	SP1552	< < >	−11.3	α	SN44 (a), srn351 (d)	Previous Study		
F1	91593	91664	72	+	SP0085	SP0086	> > >	−17.1	α		Northern	Motif 2	
F2	106533	106577	45	+	SP0103	SP0104	> > <	−5.9	α γ		No, BOX element	Motif 2	Blood
F3	117143	117248	106	+	SP0115	SP0116	> > >	−21.97	α		qRT-PCR		
F4	118118	118166	49	+	SP0116	SP0117	> > >	−11	α γ		No Signal		
F5	120678	120848	171	+	SP0117	SP0118	> > >	−51.6	α	trn0052 (d)	No, BOX element		Blood
F6	130439	130495	57	+	SP0129	SP0130	> > <	−19.9	α		Northern		
F7	209812	209917	106	+	SP0239	SP0240	> > >	−18.9	α	csRNA5, (b), SN35 (a), srn061 (d)	Northern		Lung, IN Challenge
F8	228667	228720	54	+	SP0256	SP0257	> > >	−14.5	α	csRNA1, (b), (c), SN5 (a)	Previous Study	Motif 3	
F9	231310	231359	50	+	SP0257	SP0258	> > <	−6.4	α β γ		No, BOX element		
F10	284261	284317	57	+	SP0311	SP0312	> > >	−17.9	α	trn0157 (d)	Northern	Motif 3	
F11	286614	286708	95	+	SP0312	SP0313	> > <	−17.9	α		Northern		
F12	444624	444663	40	+	SP0464	SP0465	> > <	−4.7	α γ		No, BOX element		
F13	470344	470441	98	+	SP0493	SP0494	> > >	−32.1	α	srn145 (d)	No, BOX element		
F14	499570	499689	120	+	SP0518	SP0519	> > >	−12.87	α		Northern		Nasopharynx
F15	501732	501844	113	+	SP0519	SP0520	> > <	−10.53	α	(c), srn151 (d)	Northern		
F16	528096	528162	67	+	SP0560	SP0561	< > >	−7.3	α		No, BOX element	Motif 1,5	
F17	530769	530895	127	+	SP0564	SP0565	> > >	−7.3	α	(c)	Previous Study	T-box	
F18	538437	538492	56	+	SP0571	SP0572	> > <	−14.16	α		Northern	Motif 4	
F19	543052	543150	99	+	SP0575	SP0576	> > >	−29.23	α		Northern		
F20	547541	547749	209	+	SP0578	SP0579	> > >	−49.72	α β	srn157 (d)	Northern		Nasopharynx, IN Challenge
F21	557415	557447	33	+	SP0586	SP0587	> > <	−1.3	α		No, BOX element		
F22	592573	592712	140	+	SP0625	SP0626	> > >	−36.8	α		qRT-PCR		IN Challenge
F23	603204	603346	143	+	SP0640	SP0641	> > >	−28.34	α	trn0329 (d)	No, BOX element		
F24	610528	610660	133	+	SP0641	SP0642	> > <	−32.25	α β		Northern	Motif 4	Nasopharynx
F25	623244	623345	102	+	SP0649	SP0650	< > <	−32.8	α	SN11 (a), trn0332 (d)	Northern		Blood, IN Challenge
F26	668007	668112	106	+	SP0700	SP0701	< > >	−31.18	α	SN12 (a)	Northern	Pyr	
F27	684656	684797	142	+	SP0718	SP0719	> > >	−31.95	α	trn0358 (d)	No, BOX element	TPP	Blood
F28	709064	709122	59	+	SP0749	SP0750	> > >	−26.3	α		No, BOX element		
F29	743792	743828	37	+	SP0788	SP0789	> > >	−9.2	α		No, BOX element		Lung
F30	743886	743933	48	+	SP0788	SP0789	> > >	−6.2	α γ		No, BOX element	Motif 5	
F31	810811	810862	52	+	SP0863	SP0864	< > >	−10.4	α β	(c)	Previous Study		
F32	821924	822271	348	+	SP0873	SP0874	> > <	−81.41	α β	SN16 (a), srn226 (d)	Previous Study	tmRNA	Lung, IN Challenge
F33	863736	863818	83	+	SP0910	SP0911	> > <	−17.38	α	(c)	Previous Study	Motif 2	
F34	907218	907312	95	+	SP0958	SP0959	> > >	−34.2	α	(c), SN40 (a), srn239 (d)	Previous Study		
F35	909029	909169	141	+	SP0962	SP0963	> > >	−38.03	α	srn241 (d)	Northern		
F36	941435	941487	53	+	SP1000	SP1001	> > >	−16.1	α		Northern		
F38	956782	956928	147	+	SP1012	SP1013	> > >	−45.94	α	©, srn254 (d)	Previous Study		Nasopharynx
F39	972498	972607	110	+	SP1029	SP1030	> > >	−18.52	α γ		qRT-PCR		
F40	1063101	1063151	51	+	SP1128	SP1129	> > <	−17.3			Northern	Motif 2	
F41	1071112	1071214	103	+	SP1142	SP1143	> > >	−33.08		srn277 (d)	Northern		Nasopharynx, Blood, IN Challenge
F42	1093532	1093614	83	+	SP1157	SP1158	> > <	−23.2	α		No Signal		
F43	1216148	1216246	99	+	SP1281	SP1282	> > >	−27.8	α		Northern		
F44	1220366	1220460	95	+	SP1285	SP1286	> > >	−20.74	α		Northern		IN Challenge
F45	1408204	1408275	72	+	SP1499	SP1500	> > <	−22.1	α		qRT-PCR	Motif 2 & 3	Blood
F46	1454220	1454277	58	+	SP1546	SP1547	> > <	−20.6	α		No, BOX element		
F47	1530016	1530100	85	+	SP1629	SP1630	> > >	−12.55	α β	SN24 (a)	Northern		
F48	1778293	1778427	135	+	SP1872	SP1873	> > <	−38.3	α		Northern	Motif 1 & 2 & 3	IN Challenge
F49	2026337	2026403	67	+	SP2112	SP2113	> > <	−21.9	α		No, BOX element	Motif 2	
F50	2085887	2085994	108	+	SP2168	SP2169	> > <	−27.1	α		No, BOX element	Motif 2 & 3	
F51	2133001	2133045	45	+	SP2213	SP2214	< > <	−9.9	α γ		Northern	Motif 3	Nasopharynx
F52	40336	40380	45	+	SP0041	SP0042	> > >	−16.8	α γ		No, BOX element		Nasopharynx
F53	588512	588590	79	+	SP0619	SP0620	> > <	−12.62	α		Northern		
F54	1424517	1424756	240	+	SP1516	SP1517	< > <	−64.4	α		No, BOX element	Motif 1,5	
F55	1696066	1696162	97	+	SP1777	SP1778	> > <	−11.97	α		Northern	Motif 1	
F56	158993	159090	98	+	SP0162	SP0163	> > >	−7.7			Northern		
F57	553923	553982	60	+	SP0584	SP0585	> > >	−10.6	α		No Signal		
F58	716443	716473	31	+	SP0757	SP0758	> > >	−11.2	α β γ		No Signal		
F59	869510	869591	82	+	SP0915	SP0916	< > >	−24.7	α	SN20 (a), srn235 (d)	Previous Study		
F60	950115	950165	51	+	SP1004	SP1005	> > >	−2.8	α	trn0485 (d)	No Signal		Lung
F61	972327	972384	58	+	SP1029	SP1030	> > >	−8.6	α		Northern		
F62	995726	995787	62	+	SP1059	SP1060	> > >	−24.5			Northern		Lung
F63	1090060	1090100	41	+	SP1154	SP1155	> > >	−1.5	α γ		No Signal		Nasopharynx
F64	1118416	1118462	47	+	SP1179	SP1180	> > >	−1.7	α γ		No Signal		Nasopharynx
F65	1350946	1351012	67	+	SP1431	SP1432	> > >	−19.2	α		No Signal	Motif 2	Nasopharynx
F66	2086087	2086325	239	+	SP2168	SP2169	> > <	−73.9	α	srn502 (d)	Northern		
F67	2086466	2086584	119	+	SP2168	SP2169	> > <	−25.5	α	srn503 (d)	Northern		
F68	2132994	2133050	57	+	SP2213	SP2214	> > <	−11.3	α		No Signal	Motif 3	

Columns from left to right represent the identification of the sRNA (ID), the coordinates on the TIGR4 genome to which the sRNA mapped (Start, End), the predicted size, the strand that encodes the sRNA, the genes immediately flanking the sRNA, the orientation of the sRNA and flanking genes (> = forward, < = reverse, sRNA is in center), the predicted free energy of the sRNA (delta G), the conservation of the sRNA with α = homologs in *Streptococcus pneumoniae*, β = conserved amongst streptococci, γ = conserved in other gram-positive bacteria, whether sRNA was previously identified by (a) Kumar et al., 2010, (b) Halfman et al, 2007, (c) Livny et al., 2006 or (d) Acebo et al 2012, and the method of confirmation. Conserved motifs or Rfam assignments are indicated in the Motif/Family column. If deletion of the sRNA had an effect on virulence identified by Tn-seq, the host site is indicated in the final column. If the sRNA was attenuated by IN challenge, this is also indicated in the final column.

### Sequence Analysis of sRNAs

We next sought to determine if any of the sRNAs detected by RNA-seq shared any conserved motifs that could facilitate the identification of additional sRNA candidates. Five sequence motifs were conserved across several sets of sRNAs (Figure S3 in Text S1). Each of these motifs was found at additional locations in intergenic regions in the TIGR4 genome, raising the possibility that these motifs could be used to identify additional sRNAs (Table S3 in Text S1). Part of Motif 1 shares homology with a boxA BOX element. The areas around 17 of these motifs had increased signal based on the Illumina reads compared to the nearby flanking region, indicating the possibility of sRNAs being encoded in these domains. Northern Blots using probes against flanking regions immediately outside the conserved motif for these 17 putative sRNAs identified detectable bands between 250–350 bps for each of these new putative sRNAs (Figure S1 in Text S1), indicating that the conserved motifs can be used to predict additional sRNAs. All identified sequences were also analyzed by using Rfam to identify potential RNA families. The R6 and F17 were predicted to be members of the T-box family; F26 and R15 were predicted to be members of Pyr; F27 and F32 were predicted to be members of the TPP and tmRNA families, respectively. Members of these families were found upstream of the class of genes typically regulated by cis-acting riboswitches, namely tRNA synthases and amino acid biosynthesis genes in the case of the T-box, and genes involved in pyrimidine biosynthesis for the Pyr families, indicating these regulatory RNAs may function in a similar manner. The remaining identified sequences had no significant homology to described RNA families.

### Effect of sRNAs on Pneumococcal Pathogenesis

**Figure 3:**
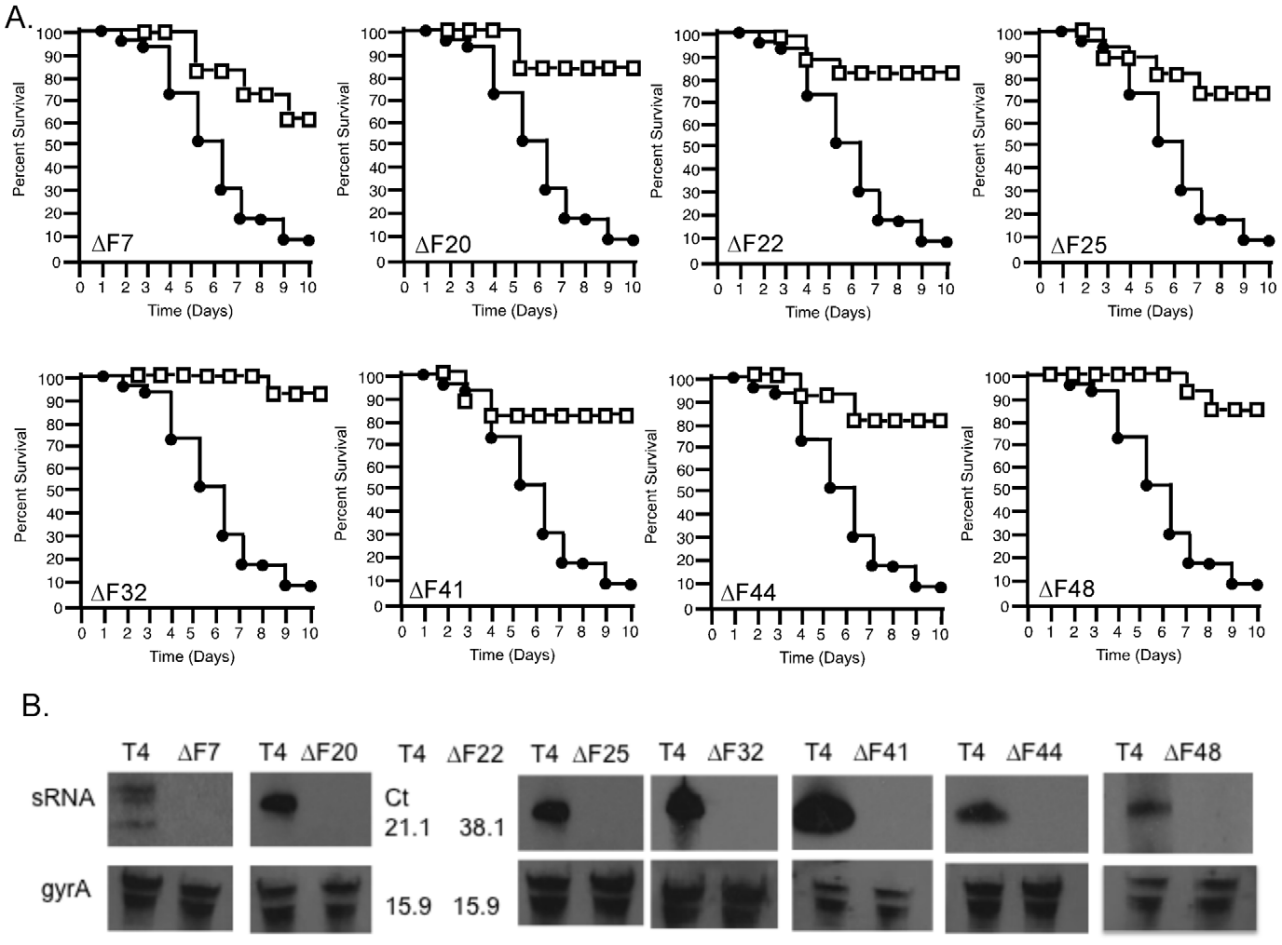
Involvement of many sRNAs in pathogenesis. **A**. Mice were challenged intranasally with the parental TIGR4 strain (filled circles) or the indicated sRNA mutant (open squares). Data represents the overall survival of at least 10 mice from 2 independent experiments. *P*<0.05 for all the mutants shown by Mantel-Cog log rank test. **B**. Northern blots of the sRNAs involved in pathogenesis arranged adjacent to the corresponding deletion mutant (top panels). Loading controls against gyrA are shown in the bottom panels. The F22 sRNA was not detectable by Northern, and hence the Ct values for the qRT-PCR for F22 and gyrA in both the TIGR4 and ΔF22 are presented.

As indicated in Figure 2, the sRNAs were next analyzed for a role in virulence. Fifteen sRNAs were chosen for further study on the basis of favorable predicted free energy for folding into secondary structures and high levels of expression by Northern blot. These included ΔF6, ΔF7, ΔF20, ΔF22, ΔF24, ΔF25, ΔF32/tmRNA, ΔF41, ΔF42, ΔF43, ΔF44, ΔF48, ΔF55, ΔR6, and ΔR12. These sRNAs were deleted with most having no polar effects on flanking genes (Figure S4 in Text S1; note SP0625 is a pseudogene and partially overlapping with ΔF22). One mutant, ΔF48 resulted in approximately 20-fold upregulation of the upstream gene *sp1872*. The mutants were assessed for their ability to establish invasive disease in a murine model of infection in which intranasal challenge progresses to pneumonia, sepsis, and meningitis. All mutants caused equivalent levels of bacteremia 24 hours post challenge (data not shown) but further progression of sepsis was attenuated in 8 of the sRNA knockouts tested (*p<0.05*, Mantel-Cox log rank test): ΔF20, ΔF32/tmRNA, ΔF41, ΔF44, ΔF48, ΔF22, ΔF7, and ΔF25 (Figure 3). These data represent the first report of sRNAs playing a definitive role in pneumococcal pathogenesis whereby deletion of the sRNA results in a significant attenuation of invasive disease.

### Global Pathogenesis Profiling of sRNAs

In order to obtain organ-specific information on the relative contribution of the identified sRNAs to pneumococcal pathogenesis, we next utilized Tn-Seq, an approach that measures the relative fitness of bacterial mutants in different environments (Figure 2, right arm of flowchart). We also included the sequences for the sRNAs identified in TIGR4 by previous studies to obtain the most comprehensive analysis of the contribution of sRNAs to pathogenesis. We analyzed three sites of the host that are vital for the progression of pneumococcal disease- the nasopharynx, lungs, and bloodstream. A comprehensive, large pool of pneumococcal mutants generated by random transposon insertions was administered to these respective host sites and bacteria were harvested subsequent to disease progression. By sequencing the respective mutants in the input and output pools, the relative fitness level of the sRNA mutants was quantified (Table 2, unfiltered data Table S4 in Text S1). A fitness level below 1 means the mutant had decreased fitness whereas a fitness level of 0 indicates that the mutant was attenuated to a degree that no mutants were recovered from the output pools. A number of sRNAs were found to have reduced fitness during colonization of the nasopharynx including F14, F20, F38, F41, F63, and F66. A further 12 sRNAs identified by other groups were also found to have significantly reduced fitness during nasopharyngeal colonization. During lung infection, sRNAs F7 and F32/tmRNA were among the 5 genes identified in our study to be significantly impaired during infection. When the comprehensive list of sRNAs was included, a total of 28 sRNA mutants were predicted to have defects during lung infection. In the sepsis model of infection, a total of 18 sRNA mutants were found to have highly significant reductions in fitness in the bloodstream, including the F25 and F41 that were amongst the knockouts originally tested. These data were in agreement with and further supportive of our data from the targeted genetic knockouts (5 of the 8 attenuated knockouts predicted from RNA-seq were also identified by Tn-seq).

**Table 2:** Tn-seq analysis of the ability of sRNA mutants to survive in different host niches.

sRNA	Host Tissue	Fitness	p-value
F7	Lung	0.49	<0.001
F29	Lung	1.15	0.005
F32	Lung	0.08	<0.001
F60	Lung	0.54	0.016
F62	Lung	0.64	0.004
R14	Lung	0.53	0.02
srn061	Lung	0.49	<0.001
srn157	Lung	0.53	<0.008
srn218	Lung	0.15	<0.001
srn226	Lung	0.08	<0.001
srn235	Lung	0.05	<0.001
srn368	Lung	0.41	<0.008
srn400	Lung	0.31	<0.001
trn0012	Lung	0.53	<0.001
trn0052	Lung	0.64	<0.008
trn0634	Lung	0.17	<0.001
SN1	Lung	0.62	<0.001
SN12	Lung	0.49	<0.001
SN16	Lung	0.09	<0.001
SN2	Lung	0.75	<0.001
SN20	Lung	0.05	<0.001
SN22	Lung	0.26	<0.001
SN26	Lung	0.43	<0.005
SN31	Lung	0.62	<0.001
SN32	Lung	0.08	<0.001
SN46	Lung	0.81	<0.005
SN5	Lung	0.34	<0.001
SN6	Lung	0.66	<0.005
F14	Nasopharynx	0.139	<0.001
F20	Nasopharynx	0.43	0.012
F24	Nasopharynx	0	0.013
F38	Nasopharynx	0	0.013
F41	Nasopharynx	0	0.013
F51	Nasopharynx	0.46	0.056
F52	Nasopharynx	0	0.013
F63	Nasopharynx	1.14	<0.001
F64	Nasopharynx	0	0.013
F66	Nasopharynx	0	0.013
R08	Nasopharynx	0	0.013
R12	Nasopharynx	0	0.013
R16	Nasopharynx	0.42	0.02
srn142	Nasopharynx	0	<0.003
srn254	Nasopharynx	0	<0.003
srn277	Nasopharynx	0	<0.003
srn502	Nasopharynx	0	<0.003
srn502	Nasopharynx	0	<0.003
trn0156	Nasopharynx	0	<0.003
trn0760	Nasopharynx	0	<0.003
trn0830	Nasopharynx	0	<0.003
SN27	Nasopharynx	0.34	<0.01
SN30	Nasopharynx	0	<0.002
SN39	Nasopharynx	0	<0.002
SN46	Nasopharynx	0	<0.002
SN50	Nasopharynx	1.13	<0.002
F2	Blood	0.533	0.188
F5	Blood	0	0.005
F25	Blood	0.824	0.007
F27	Blood	0.895	0.001
F41	Blood	0.334	0.002
F45	Blood	0.836	0.003
R04	Blood	0.273	0.006
R08	Blood	0.747	0.014
R12	Blood	0.742	0.003
srn279	Blood	1.34	<0.003
trn1025	Blood	1.22	<0.003
trn0830	Blood	0.74	<0.005
trn0012	Blood	0	<0.003
trn0052	Blood	0	<0.003
SN11	Blood	0.82	<0.01
SN27	Blood	0.86	<0.01
SN38	Blood	0	<0.002
SN46	Blood	1.19	<0.002

Transposon mutant libraries were used to determine the ability of sRNAs to survive when inoculated into the nasopharynx or lung or to establish bacteremia in the blood following intraperitoneal injection of bacteria. Fitness value of 1.0 indicates no fitness benefit or defect compared to TIGR4. Values <1.0 indicate a fitness defect in the respective model while values >1.0 indicate a relative fitness benefit. When no mutants were present in the input pool, the sRNA was eliminated from the analysis while a fitness value of 0 indicated a mutant which was present in the input sample but was not recovered in the output pool.

**Figure 4:**
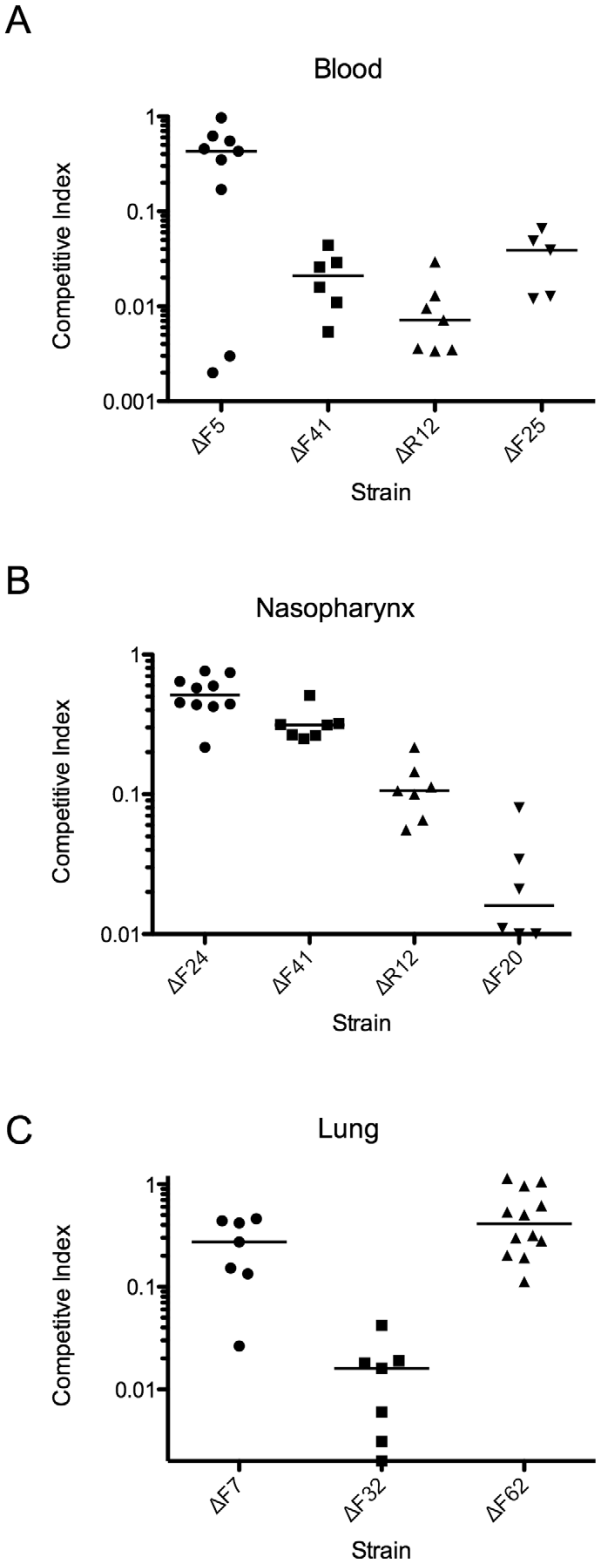
Confirmation of tissue-specific fitness defects of sRNA mutants identified as attenuated by Tn-seq. Mice were challenged with the sRNA mutants and parental TIGR4 in a competitive index model of infection whereby equivalent CFUs of the wild type and each mutant are administered simultaneously to a specific body site and the bacterial density is measured 24 hours post infection by differential plating. The competitive index of the respective mutants in the blood (A), nasophaynx (B), and lungs (C) are indicated. Competitive index of 1 represents equivalent amounts of wild type and mutant bacteria were recovered. Each data point represents a single mouse. All experiments were performed in duplicate. *P*<0.05 by Mann-Whitney for all sRNA mutants.

In order to confirm the Tn-seq analysis, individual sRNA knockouts were tested in a competitive index model of infection in which the sRNA mutant was inoculated together with the TIGR4 wild type into the nasopharynx, lung, or blood and differential bacterial density was determined at 24 hours post infection. The capacity of a subset of sRNA mutants predicted by Tn-seq to colonize the nasopharynx, infect the lungs, and replicate in the bloodstream were analyzed in respect to TIGR4 (Figure 4A–C). The ΔF24 strain which was avirulent in sepsis showed a slight decrease in colonization of the nasophaynx (Figure 4B). In addition, ΔR12, which was not significantly attenuated in our initial model of infection, showed dramatic differences in both nasopharyngeal colonization and in the intraperitoneal bacteremia model (Figure 4). In addition, two new sRNA mutants were generated from the Tn-Seq predictions, ΔF5 and ΔF62, both of which displayed defects in their respective host niches of the bloodstream and lung.

RNA-seq coupled with Tn-seq and validated with targeted knockout mutants proved to be a robust method for determining the contribution of sRNAs to pathogenesis. A total of 28 sRNAs in the lung, 26 in the nasopharynx, and 18 in the blood were predicted to have significantly altered fitness in these respective host niches. While a majority of the Tn-seq sRNA mutants attenuated the bacteria, it should be noted that a small number of mutations actually resulted in a fitness benefit in certain host sites (Table 2). In addition, most of the attenuated sRNAs were predicted to be defective in only one host organ, underscoring the contribution of these sRNAs to these distinct environments. These data indicate that sRNAs contribute to pneumococcal pathogenesis both for systemic infections as well as for tissue specific tropisms.

### Adhesion and Invasion Capacity of Attenuated sRNA Mutants

To identify the step in host-bacterial interactions affected by the attenuated sRNA knockouts, the ability of the mutants to adhere to and invade endothelial and nasopharyngeal cell lines was determined. The sRNA mutant F20 had a significant defect in adhesion and invasion of Detroit nasopharyngeal cells (Figure S5 in Text S1), a finding in agreement with the decreased nasopharyngeal fitness (Table 2). A striking defect in adherence to activated endothelial cells was observed in six of the sRNA mutants, while invasion of endothelial cells was only impaired in F20 and F32/tmRNA. These data indicate that many of the attenuated sRNAs have specific defects in interactions with host cells, an underlying cause for attenuation of disease.

### Identification of Putative Targets

We then hypothesized that sRNAs could target either gene networks or individual genes. To investigate global gene expression, we compared the transcriptome of TIGR4 to that of each of the attenuated sRNA mutants via microarray analysis. Several pathways were significantly different upon deletion of the sRNAs (Table S4 in Text S1). The ΔF25, ΔF41, and ΔF44 mutants upregulated a putative ABC transporter encoded by SP1688–1690 that is predicted to be involved in carbohydrate transport. The SP1721–1725 genes, predicted to play roles in sucrose metabolism, were also highly differentially regulated in several of the sRNA mutants. The ΔF32 mutant substantially downregulated several metabolic networks encompassing the lactose transport system and multiple PTS systems. This highlights the potentially pleiotropic effects that the deletion of the sRNAs could have on pneumococcal biology and pathogenesis in the host.

**Figure 5:**
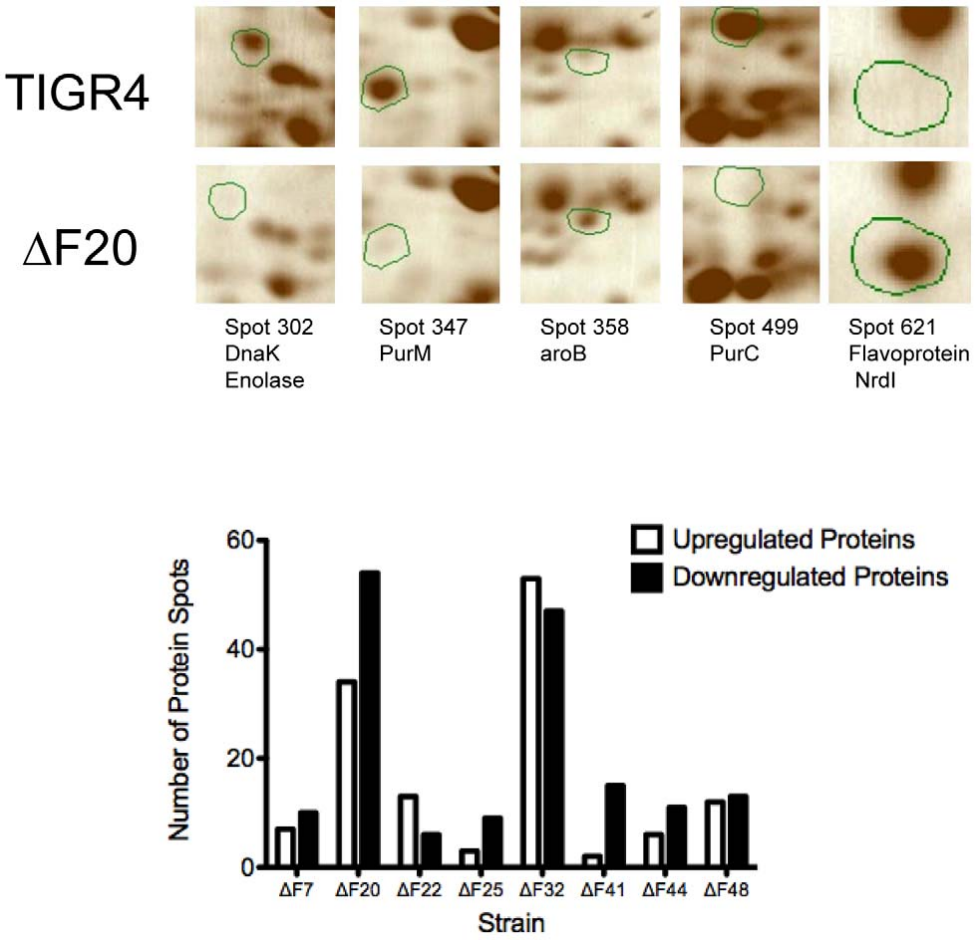
Proteomic analysis of attenuated sRNA mutants. Duplicate 2D gels of protein lysates prepared from the parental TIGR4 strain and the eight attenuated sRNA mutants were run and the individual protein spots were quantified (example shown in Figure S6 in Text S1). A representative subset of identified proteins showing altered expression in the ΔF20 strain compared to TIGR4 is shown in the upper panel (green circles). The number of polypeptide spots altered in each mutant vs TIGR4 by a fold increase of >1.7 and *p* value<0.05 (t-test) or a fold increase of >3.0 are enumerated in the bottom panel.

Many sRNAs function at the post-transcriptional level [43], suggesting that there may be important changes in bacterial physiology that potentially could have been missed by global transcriptional analysis. We next sought to determine the effect of the deletion of sRNAs on the global proteome of the pneumococcus. Replicate two-dimensional gels were analyzed for each attenuated sRNA mutant and compared to the parental TIGR4. Every individual protein spot on the gels was then quantified from these duplicate gels to obtain a comprehensive analysis of changes in protein abundance resulting from the deletion of the respective sRNA. The quantitation of the respective spots for each bacterial strain, along with both the predicted pI and molecular weight of the protein, are listed in Table S6 in Text S1. The image of a TIGR4 gel with the individual spot identifications is provided in Figure S6 in Text S1. A number of proteins spots found in increased or decreased abundance are summarized in Figure 5. Deletions in F20 and F32/tmRNA resulted in dramatic alterations in abundance, of 88 and 100 proteins respectively. Of note is that both the ΔF20 and ΔF32 mutants were the only attenuated sRNA mutants to have significant defects in the invasion of endothelial cells, indicating that a subset of these misregulated proteins are important for cell-cell interactions. Analysis by mass spectrometry (Figure 5) indicated that the ΔF20 mutant had decreased abundance of two proteins involved in purine biosynthesis, PurM and PurC, potentially explaining the defect in virulence. The overexpression of the NrdI flavoprotein, essential for the conversion of nucleotides to deoxynucleotides, suggests defects in DNA synthesis and repair [44]. These data indicate that the deletion of sRNAs can have multiple effects on bacterial pathogenesis by influencing numerous putative targets.

## Discussion

Advances in sequencing technologies have driven an explosion in our knowledge of the non-coding genetic repertoire of bacterial species. This study illustrates the first example of a global approach to both sRNA identification and pathogenesis profiling, an amalgamation of RNA-seq and Tn-seq. The RNA-seq tactic identified 89 putative pneumococcal sRNAs, capturing both sRNAs previously detected by sequencing and tiling arrays and many additional previously unknown sRNAs [11], [12], [37], [39]. Use of RNA-seq has certain advantages for the identification of sRNAs. The mean level of sequence coverage was over 100-fold on both the forward and reverse strands, with each sRNA corresponding to a minimum of 10x coverage allowing for high confidence in the data. It should be noted that low abundance sRNAs identified in other studies from a single read will likely be missed by our analysis [39]. Unlike tiling arrays, RNA-seq identifies the origin of transcription. This permits the precise mapping of sRNAs that contain highly repetitive regions, such as the over 100 BOX elements found in intergenic regions of the pneumococcal genome. BOX elements are short AT-rich repeats that are highly transcribed and were also detected in sRNA searches using tiling arrays, though precise locations could not be mapped [11]. Eighteen BOX element containing sRNAs were mapped, a finding particularly important as the Tn-seq analysis implicated a subset of four BOX-element sRNAs in pathogenesis. Although BOX elements have traditionally been thought to be parasitic sequences mobilized by transposases [45], recent evidence supporting their placement in sRNAs indicates that they can form RNA structures with riboswitches [46]. In addition, BOX elements can stimulate expression of downstream genes by increasing the half-lives of the mRNA [47].

Another important aspect of this study was the identification of five novel shared sRNA sequence motifs that were conserved at multiple locations in the pneumococcal genome. Upon closer examination of the sequence read depth in the areas surrounding these motifs, we identified 17 with increased signal compared to the surrounding region. All 17 of these predicted sRNAs were subsequently validated by expression analysis underscoring the robustness of the predictions. While members of the T-box, Pyr, TPP, and tmRNA sRNA families described in other bacteria were also found in pneumococcus, a majority of the predicted pneumococcal sRNAs could not be assigned to a functional family. These data indicate that the pneumococcus is a rich source of new motifs that can expand sRNA prediction algorithms in Gram-positive bacteria.

Although numerous sRNAs have been identified in the pneumococcus, there have been no sRNAs implicated in pathogenesis and more broadly, there have been no attempts to apply transposon-mediated mutagenesis to determine the role of sRNAs in bacterial virulence in specific host tissues. This study represents the first use of transposon-mediated mutagenesis to address the global role of sRNAs in discrete host tissues during disease. Using a comprehensive list of sRNAs identified in this study together with those found by others, we identified a number of sRNAs that played distinct roles in pathogenesis in the nasophaynx, the lung, or the bloodstream. The lungs provided the most comprehensive analysis of the contribution of sRNAs to virulence, since bottleneck constraints in the nasophaynx and the blood imposed by a limitation of bacterial binding sites and clearance by the spleen, respectively, may have impaired detection in these sites. A number of sRNAs had no inserts in the Tn-seq deletion library (n.i. in Table S4 in Text S1) and it is tempting to speculate that there is a selective pressure against the loss of these sRNAs; however this observation could be random due to their small size. All three body sites had a distinct list of sRNA candidates that were involved in pathogenesis. The Tn-seq analysis proved to be robust, as mutants predicted to be attenuated in their respective host niches were confirmed in *in vivo* competition experiments pitting each sRNA mutant individually against wild type (Figure 4). Thus the multi-organ Tn-Seq approach captured this diversity as exemplified by R12 that did not have a significant virulence defect in overall survival in our initial studies but was attenuated both during colonization of the nasophaynx and in the blood following intraperitoneal infection. The Tn-seq analysis also provides insight into the organ-specific defects of the sRNAs found to have reduced virulence in Figure 3. Both the ΔF41 and ΔF25 strain had greatly reduced fitness in the blood, in agreement with their inability to progress to sepsis. The ΔF7 and ΔF32/tmRNA strains were both defective in the lung infection, indicating that this might be the most crucial site for clearance of these mutants. This comprehensive analysis of the contribution of all the identified sRNAs to pneumococcal pathogenesis in discrete host sites can provide a framework for future investigations elucidating the precise functions of these sRNAs. These data add to the growing understanding of the contribution of sRNA in the virulence of bacterial pathogens [3].

The sRNA mutants displaying defects in virulence exhibited a number of characteristics that could potentially explain an inability to cause disease. Several of the attenuated sRNA mutants had defects in adhesion and invasion of nasopharyngeal or endothelial cells, capabilities important to the progression of invasive disease. ΔF20 and ΔF32/tmRNA showed decreased adhesion/invasion of nasopharyngeal or endothelial cells, respectively, in concert with Tn-seq and competitive index data indicating lack of fitness in the nasopharynx and lung. F32 encodes a tmRNA and these have been implicated in the pathogenesis of other bacteria [48], [49]. The central role of tmRNA in the rescue of ribosomes on stalled mRNA as well as targeting defective mRNA for degradation, is consistent with the strong defect in pathogenesis observed in the ΔF32 strain [50]. In the case of the ΔF20 mutant, proteomic analysis indicated proteins responsible for purine metabolism were strongly down regulated whereas DNA synthesis and repair pathways were greatly increased. Thus deletion of F20 had pleiotropic effects on DNA metabolism that could explain attenuation of the mutant. Taken together, these data provide compelling evidence that sRNAs play important roles in virulence, that their affects can arise at several levels of control of virulence gene/protein expression, and that these roles can be restricted to specific host tissues.

Our study expanded the search for sRNAs and their role in gene regulation to three mutants in TCSs. Control over gene networks by TCSs is typically mediated by a direct interaction of the response regulator with a target sequence shared by many genes dispersed over a genome. However, TCSs have also been found to control the expression of sRNAs in pneumococcus and other bacteria [37], [51]. For example, control of porin expression in *E. coli* involves multiple sRNAs that exert posttranscriptional control over the targets of TCSs [42]. The prospect of sRNA functioning as an intermediary, finely tuning the control of and expanding the regulatory scope by a TCS, would allow for another layer of control for more precise regulation. Our observation that the abundance of sRNAs was altered when each of the three TCSs were disrupted is consistent with TCSs acting through sRNAs to broadly control gene expression. This is further supported by the observed alterations of the global transcriptome as well as the abundance of multiple protein targets upon deletion of an individual sRNA (Tables S5 and S6 in Text S1, Figure 5). These data suggest that the impact of sRNAs on multiple aspects of pneumococcal biology and pathogenesis could potentially be exerted by an additional layer of posttranscriptional control over the gene networks controlled by TCSs.

The widespread utilization of RNA-mediated regulation of diverse processes has a number of potential advantages for bacteria [52]. Protein regulators incur greater metabolic costs to the cell, being encoded by larger segments of the genome and requiring translation. In contrast, sRNAs do not require translation and occupy a very limited amount of the genome. The additional layer of regulation conferred by sRNAs may also allow for more precise control of gene expression, as evidenced by the fact that sRNAs can have multiple targets as well as the fact that multiple sRNAs can regulate a single target under different conditions [4], [53]. Additionally, sRNAs can have dramatically different half-lives in the cell, ranging from under 2 minutes to greater than 30 minutes [54]. Such differences in stability could potentially mediate the duration of control mediated by sRNAs. The challenging task that remains following the identification and characterization of sRNAs in pathogenesis is assigning discrete functional roles to these molecules. We have shown the feasibility of applying Tn-seq to identify changes in bacterial fitness in response to deletion of the corresponding sRNA in various host tissues. The feasibility of this approach to investigate the gene networks and functional roles of sRNAs suggest the combination of RNA-seq and Tn-seq will be a unique and powerful tool for future investigations of the precise functional roles of these sRNAs in the pneumococcus.

## Materials and Methods

### Bacterial Strains and Growth Conditions

The *S. pneumoniae* strains used are listed in Table S1 in Text S1. All experiments were conducted in the sequenced TIGR4 strain [55]. Cultures were grown overnight on tryptic soy agar plates supplemented with 3% sheep blood and were transferred to a defined semisynthetic casein liquid medium supplemented with 0.5% yeast extract (i.e., C+Y) [56].

### Small RNA Purification and Sequencing

To initially identify sRNAs in *Streptococcus pneumoniae*, we designed a method to isolate, enrich, and fully sequence small (<200 nt) transcripts of the TIGR4 strain of pneumococcus. Cultures were grown in triplicate in C+Y (200 mL) until an OD_620_ of 0.5 was reached, corresponding to mid log phase growth. Bacteria were diluted (1∶2) in RNAProtect stabilization buffer (Qiagen) and centrifuged; the resulting bacterial pellets were then frozen at −80°C. The pellets were thawed and resuspended in Lysis Buffer Mirvana miRNA Isolation Kit (Applied Biosystems). To each sample, 200 µL of 0.1 mm glass beads (Sigma) were added before they were lysed using a mini-beadbeater. Samples were incubated for 10 minutes at 70°C and subsequently processed through a Qiashredder column (Qiagen). sRNA was purified using organic extraction and sRNA enrichment procedures as described in the Mirvana protocol. Purified sRNA was DNAse-treated by using Turbo DNAse (Applied Biosystems) according to the manufacturer's instructions. Purified sRNA was prepared for sequencing by using the Small RNA Sample Prep kit (Illumina). Details about the cluster generation, sequencing, and Northern Blot confirmation are provided in the Supplementary Materials section.

### Small RNA Candidate Region Selection

Detection of biologically meaningful sRNA regions was based on the assumption that sequence reads are enriched in such regions. The sequence reads were first mapped to the T4 genome using the program GMAP recursively by quality based trimming. Then the coverage information for both strands was calculated based on high quality matches. When a read mapped to multiple positions on the genome, the highest quality match was selected. For each intergenic region and anti-sense coding region of size greater than 150 bases, a simple method was used to identify a potential read enriched region (peak). Due to the degradation of the sample mRNA, these reads were mapped all over the genome and it was necessary to remove those background signals. Signal noise was not uniformly distributed along the genome, so a baseline detection algorithm (linear interpretation of minimum value) was used. After baseline correction, a cut off value of 20 was utilized to identify potential peaks such that any consecutive region with minimum coverage of 20 is considered as a potential peak. The peak detection methods were applied on both strands separately.

These detected peaks were subjected to further biological constraints. First, a promoter region would be expected on the upstream sequence. We used the Prokaryotic promoter prediction program (http://bioinformatics.biol.rug.nl/websoftware/ppp/ppp_start.php) to search for promoters. Second, a rho-independent terminator would be expected downstream of the sequence. We used the TransTermHP (http://transterm.cbcb.umd.edu/index.php) predicted terminator for the T4 genome. For each potential peak, the promoter must appear between −75 and 20 bases around peak starting position and the terminator must present between −20 and 75 bases around the peak ending position. Those two criteria remove 83% to 98% of potential peaks. These criteria are similar to those used previously to identify candidate sRNAs using RNA-seq data [57], [58].

### sRNA Mutagenesis

Mutants were made by using PCR-based overlap extension [59]. Briefly, regions upstream and downstream of the target region were PCR-amplified and spliced into an antibiotic resistance cassette. The final PCR product was transformed into the pneumococcus by conventional methods, replacing the targeted region with the antibiotic resistance cassette. To confirm transformation, primers outside of the transformed region were used for PCR and subsequent region sequencing. The lists of mutants made and oligonucleotides used are included in Tables S1 and S2 in Text S1, respectively.

### Microarray Analysis

Bacterial RNA was harvested from mid–log phase cultures (OD_600_ = 0.4) grown in C+Y by using the Qiagen RNAeasy minikit. Microarray experiments were performed as described previously [60]. Briefly, whole-genome *S. pneumoniae* version 8.0 cDNA microarrays were obtained from the Pathogen Functional Genomics Resource Center (PFGRC). Microarray experiments were performed by the Functional Genomics laboratory, Hartwell Center for Bioinformatics and Biotechnology, St. Jude Children's Research Hospital using standard protocols provided by PFGRC (http://pfgrc.tigr.org/protocols.shtml) as previously described [61].

### sRNA Sequence Analysis

Secondary structures were predicted using mfold to obtain ΔG values [62]. The MEME program was used to perform the MOTIF search. The meme web server was used with default options although negative training sequences were used to delineate true motifs from the background sequence patterns of *S. pneumoniae*.

### Proteomics

Proteomic profiling was performed by Kendrick Laboratories Inc (Madison, WI). Two-dimensional electrophoresis was performed using the carrier ampholine method of isoelectric focusing. Isoelectric focusing was carried out in glass tubes of inner diameter 2.3 mm using 2% pH 4–8 mix Servalytes (Serva, Heidelberg Germany) for 9,600 volt-hrs. Fifty ng of an IEF internal standard, tropomyosin, was added to each sample prior to loading. After equilibrium in SDS sample buffer (10% glycerol, 50 mM dithiothreitol, 2.3% SDS and 0.0625 M tris, pH 6.8), each tube gel was sealed to the top of a stacking gel that overlays a 10% acrylamide slab gel (0.75 mm thick). SDS slab gel electrophoresis was carried out for about 4 hrs at 15 mA/gel. The following proteins (Sigma Chemical Co, St. Louis, MO) were added as molecular weight markers: myosin (220,000), phosphorylase A (94,000), catalase (60,000), actin (43,000), carbonic anhydrase (29,000), and lysozyme (14,000). The gels were dried between cellophane sheets with the acid end to the left. Duplicate gels were obtained from each sample and were scanned with a laser densitometer (Model PDSI, Molecular Dynamics Inc, Sunnyvale, CA). The scanner was checked for linearity prior to scanning with a calibrated Neutral Density Filter Set (Melles Griot, Irvine, CA). The images were analyzed using Progenesis Same Spots software (version 4.5, 2011, Nonlinear Dynamics, Durham, NC) and Progenesis PG240 software (version 2006, Nonlinear Dynamics, Durham, NC). The general method of computerized analysis for these pairs included image warping followed by spot finding, background subtraction (average on boundary), matching, and quantification in conjunction with detailed manual checking.

Spot % is equal to spot integrated density above background (volume) expressed as a percentage of total density above background of all spots measured. Difference is defined as fold-change of spot percentages. For example, if corresponding protein spots from different samples (e.g. mutant versus wild type) have the same spot %, the difference field will show 1.0; if the spot % from the mutant is twice as large as wild type, the difference field will display 2.0 indicating 2-fold up regulation. If the spot % from the mutant has a value half as large, the difference field will display – 2.0 indicating a 2-fold down regulation.

A subset of proteins were chosen for further analysis. Protein spots were excised from duplate Coomassie stained gels. The protein sample was digested with trypsin and mass spectrometric analysis was performed using an Orbitrap Velos Mass Spectrometer from Thermo Electron (San Jose, CA). This instrument employs electrospray ionization (ESI), in conjunction with an Orbitrap mass analyzer. The digest was introduced into the instrument via on line chromatography using reverse phase (C18) ultra-high pressure liquid chromatography on the nanoAcquity (Waters, MA). The column used was a New Objective C18 with an I.D. of 75 um and bed length of 10 cm. The particle size was 2.7 um. Peptides were then gradient eluted into the linear ion trap through a non-coated spray needle with voltage applied to the liquid by increasing the concentration of acetonitrile. Data acquisition involved acquiring the peptide mass (MS) spectra followed by fragmentation of the peptide to produce MS/MS spectra that provides information about the peptide sequence. Database searches were performed using raw files in combination with the Mascot search engine. Protein/peptide assignments are made on the basis of MS/MS spectra.

### Adhesion and Invasion Assays

Detroit nasopharyngeal cells and rBCEC6 rat brain capillary endothelial cells were grown in 24-well plates at 37°C in 5% CO_2_ to 80% confluency and activated with TNF-α (10 ng/mL) for 2 hours [40]. Pneumococcal cultures were grown until the OD_620_ was 0.5, washed with PBS, and then added to eukaryotic cells (1×10^7^ cfu/well). Three wells were used for each mutant or TIGR4R and the assays were repeated a minimum of 3 times. For adherence assays, cells were incubated 30 minutes with bacteria, a time chosen to minimize internalization of adherent bacteria. After washing 3x in dPBS, the cells were released from the plate with trypsin but not lysed before plating on blood agar plates. Colonies grown overnight were counted as bacteria adherent to cells. For invasion assays, cells were incubated with the bacteria for 2 hours, washed 3 times in dPBS, and subjected to 1 hour of treatment with penicillin (10 µg/mL) and gentamycin (200 µg/mL). The cells were washed, trypsinized, and lysed with 0.025% Triton X-100. The lysates were then incubated overnight on blood agar plates and the resulting colonies were counted.

### Mouse Challenge

All mice were maintained in BSL2 facilities, and all experiments were done while the mice were under inhaled isoflurane (2.5%) anesthesia. For survival studies, bacteria were introduced by intranasal administration of 10^7^ CFU of bacteria in PBS (25 µL), a model which effectively recapitulates the progression of disease from nasopharyngeal colonization, to pneumonia, and finally to the development of sepsis and meningitis [63]. A minimum of 10 mice per group was used in the studies from at least two independent experiments. Mice were monitored daily for signs of infection, and differences in time-to-death among the mice were compared via Mantel-Cox log rank test. For the competitive index studies, equivalent CFUs of the parental TIGR4 and the respective mutants were administered to the mice. For nasopharyngeal colonization, bacteria were administered at a dose of 10^7^ CFU in 25 µL PBS [40]. Bacteria were administered intratracheally at a dose of 10^5^ CFU in 100 µL PBS to model lung infection [40]. For the sepsis model, 2×10^3^ CFU in 100 µL PBS was administered by intraperitoneal injection [63]. Tissues and blood were collected from all animals 24 hours following infection. For lung collection, mice were perfused with saline prior to organ collection to remove contaminating blood from the lung which was then homogenized. The parental TIGR4 and sRNA mutants were enumerated by serial dilution and counting on TSA blood agar plates with and without erythromycin. The CFU counts were then utilized to calculate competitive indexes [64] (1 = equivalent numbers of mutant recovered to TIGR4).

### Tn-seq for Bacterial Fitness

Tn-seq, both the experimental procedure as well as data analysis, was performed essentially as described previously [65], [66]. For two time points (t_1_ and t_2_) the number of reads at each genome location was determined by massively parallel sequencing on an Illumina Genome Analyzer II. Mice were challenged with transposon mutant libraries administered directly to the nasopharynx, lungs, or to the bloodstream. On average, 250 reads were mapped per insertion/time point. Since insertions with a very low number of reads that slightly fluctuate over time can influence the data disproportionately, only insertions with fifteen or more reads at t_1_ are included in the analyses. For each insertion, fitness *W_i_*, is calculated by comparing the fold expansion of the mutant relative to the rest of the population with the following equation [67]:
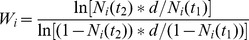
In which *N_i_*(*t_1_*) and *N_i_(t_2_)* are the frequency of the mutant in the population at the start and at the end of the experiment, respectively, and *d* (expansion factor) represents the growth of the bacterial population during library selection. Details regarding the data analysis and methodology are included in the Supplementary material.

### Ethics Statement

All experiments involving animals were performed with prior approval of and in accordance with guidelines of the St. Jude Institutional Animal Care and Use Committee (Protocol #250). The St Jude laboratory animal facilities have been fully accredited by the American Association for Accreditation of Laboratory Animal Care. Laboratory animals are maintained in accordance with the applicable portions of the Animal Welfare Act and the guidelines prescribed in the DHHS publication, Guide for the Care and Use of Laboratory Animals.

## Supporting Information

Text S1 **Supplementary Materials.** This file includes the following: Supplementary Methods; Strains used in this study (Table S1), Primers used in this study (Table S2); Coordinates of the additional regions of the TIGR4 genome containing the conserved motifs identified in Figure S4 (Table S3); Raw data from the Tn-seq screen (Table S4); Microarray analysis of attenuated sRNA mutants (Table S5); Proteins showing differences in abundance in the attenuated sRNA mutants compared to the parental TIGR4 (Table S6); Northern blots of sRNAs identified by RNA-seq (Figure S1); Differential expression of sRNA F13 in TCS response regulator knockouts (Figure S2); Motifs overrepresented in the sRNA sequences identified using the MEME software to identify motifs (Figure S3); qRT-PCR analysis of the genes flanking the sRNA knockouts (Figure S4); Adhesion and invasion of TIGR4R and the attenuated sRNA mutants to activated nasopharyngeal (Detroit) and endothelial (RBCEC6) cells (Figure S5); Representative 2D gel utilized for the proteomic analysis of TIGR4 with the individual spots being identified by circles (Figure S6).
(DOC)
