# Supplementary material for: Control of Virulence by Small RNAs in Streptococcus pneumoniae
Source: PLoS Pathog. 2012 Jul 12;8(7):e1002788. doi: 10.1371/journal.ppat.1002788 (PMC3395615; doi:10.1371/journal.ppat.1002788)

**Supplementary Information**

**Methods**

**Cluster Generation and Sequencing**

Each sample was run in triplicate over the course of two separate sequencing runs. In the initial run 3pM of GRR, vncR and CbpR were run in duplicate in six of the seven lanes that were available to sequence experimental samples. T4R was run individually, as was a 3pM PhiX DNA sample, which was used as a cluster and sequencing control. For the preliminary run, the base calls were analyzed using Illumina's Pipeline version 1.4.0. The number of clusters that passed filter varied from 2.87 Million reads (vncR2) to 11.6 Million reads (cbp1), with an average of 6.9 million reads per lane.

In the secondary run T4R was run in duplicate, while GRR, vncR and CbpR were run singularly. The base calls for this run were analyzed using Illumina's CASAVA (formerly Pipeline) version 1.6.0. The number of clusters that passed filter varied from 10.9 Million reads (GRR2) to 20.3 Million reads (cbp3), with an average of 16 million reads per lane. The reads on the single read flowcell were subjected to 150 cycles of sequencing on the Illumina Genome Analyzer II (initial run) and Genome Analyzer IIx (secondary run) platforms. The CLCGenomic Workbench package was used to trim the reads based on the quality scores with a limit of 0.05 (approximate Phred score = 13). Reads that were shorter than 18 bp in length post-trimming were discarded. Reads were mapped back to the TIGR4 reference genome (AE005672).  The mapped read lengths varied from 18 - 150 bp post trimming, with an average size of approximately 85bp.

**Small RNA candidate region selection**

Detection of biologically meaningful sRNA regions was based on the assumption that sequence reads are enriched in such regions. The sequence reads were first mapped to the T4 genome using the program GMAP recursively by quality based trimming. Then the coverage information for both strands was calculated based on high quality matches. When a read mapped to multiple positions on the genome, the highest quality match was selected. For each intergenic region and anti-sense coding region, a simple method was used to identify a potential read enriched region (peak). Due to the degradation of the sample mRNA, these reads were mapped all over the genome and it was necessary to remove those signals. Signal noise was not uniformly distributed along the genome, so a baseline detection algorithm (linear interpretation of minimum value) was used. A cut off value of 20 was utilized to identify potential peaks.

These detected peaks were subjected to further constraints. First, a promoter region would be expected on the upstream sequence. We used the Prokaryotic promoter prediction program (<http://bioinformatics.biol.rug.nl/websoftware/ppp/ppp_start.php>) to search for promoters. Second, a pho-independent terminator would be expected downstream of the sequence. We used the TransTermHP (<http://transterm.cbcb.umd.edu/index.php>) predicted terminator for the T4 genome. Reads were then uniquely aligned to the T4 genome for pairs of control and experimental strains using Mosaik with the unique parameter set. Ace, wig, Bed files were provided as tracks. The pairwise differences in depth at a base for aligned regions was converted into a log-ratio. A traditional frequency analysis was conducted using the log of the likelihood ratio for the probability of a read covering a base (see below). This analysis is essentially the same as the readBase methodology used in the Illumina Casava package.

**Northern blot analysis**

Total RNA (3 µg) was run on a 10% TBE-urea gel at 200V for 75 minutes. The gel was briefly stained in ethidium bromide (0.5mg/mL) and imaged before its RNA was transferred to a Brightstar Plus nylon membrane (Applied Biosystems) at 35V for 30 minutes and crosslinked at 1200µJ cm^−2^ by using a Stratalinker UV crosslinker (Stratagene). Crosslinked membranes were incubated for 30 minutes at 50°C in DIG EasyHyb prehybridization buffer (Roche) with 2% blocking buffer and poly(A) solution (0.01mg/mL). For each different RNA sample, a single oligonucleotide was synthesized (Integrated DNA Technologies) that was complementary to the first 50 bp of the transcript. Oligonucleotides, listed in Supplementary Table 2, were DIG-labeled by using the DIG Oligonucleotide Tailing Kit (Roche).

DIG-labeled probe (50 pmol) was added to fresh EasyHyb buffer (5 mL) and then the blots were incubated with hybridization buffer overnight at 50°C. After high- and low-stringency washes, blots were further washed with solutions from the DIG Wash and Block Buffer Kit (Roche) and then CDP-Star was added as the substrate (Roche). Blots were exposed to X-ray film for 5–20 minutes. The size of each transcript was determined by comparing its corresponding band on the developed film to the sRNA ladder on the ethidium bromide–stained gel.

**Quantitative RT-PCR**

The amount of purified mRNA was measured by using a NanoDrop spectrophotometer (ThermoScientific) and appropriate RNA standards. Both cDNA synthesis and quantitative PCR was performed by using the Superscript III Platinum SYBR Green Two-Step qRT-PCR kit (Invitrogen, San Diego, CA) and an ABI PRISM 7300 Real-Time PCR system (Applied Biosystems). Each gene’s relative transcript abundance was normalized to the amount of *gyrA* transcript, which served as an internal control. The qRT-PCR experiments were done in triplicate using independently isolated RNA samples.

**Tn-seq**

Plasmid DNA harboring *magellan6,* a derivative of the Himar1 Mariner transposon, was purified from *E. coli* with the Qiagen mini plasmid preparation kit (Qiagen). *S. pneumoniae* DNA was isolated with the Qiagen Blood and Tissue kit from an exponentially growing culture. *In vitro magellan6* transposition reactions were carried out with purified MarC9 transposase, 1 µg of *S. pneumoniae* target DNA and 1 µg of *magellan6* plasmid DNA. Reactions were incubated for 1 hour at 30^o^C, inactivated for 20’ at 72^o^C, ethanol precipitated and resuspended in gap repair buffer [50 mM Tris (pH 7.8), 10 mM MgCl_2_ 1 mM DTT, 100 nM dNTPs and 50 ng BSA]. Repair of transposition product gaps was performed with *E. coli* DNA ligase overnight at 16^o^C after which repaired transposition products were transformed into naturally competent *S. pneumonia TIGR4*. The following day transformants were scraped off of blood plates, pooled into six libraries of approximately 1,500 to 4,000 transformants/library, split up into multiple starter cultures and stored at -20 ^o^C.

**Tn-seq Sample preparation and Illumina sequencing**

DNA from two time points, pre-selection (t_1_) and post-selection (t_2_), was digested for 2.5 hours at 37^o^C with MmeI (NEB), the 5’ phosphate group was removed with Calf intestinal alkaline phosphatase (NEB) after which DNA was phenol/chloroform extracted, ethanol precipitated and dissolved in H_2_O. An adapter was ligated with T4 DNA ligase (NEB) onto the overhang left by MmeI after which a PCR was performed with the adapter-ligated samples as template. Of the two primers used in the reaction one was complementary to the mini-transposon inverted repeat sequence while the other was to the adapter. The resulting PCR product was 120 bp in length and was amplified with the following parameters: 95^o^C for 30 s, followed by 22 cycles of 10 s at 95^o^C, 25 s at 55^o^C and 15 s at 72^o^C, followed by 10 min at 72^o^C. The PCR product was gel purified, dissolved in H_2_O and sequenced on an Illumina Genome Analyzer II according to the manufacturers protocol (Illumina). Libraries harvested from one to three mice were multiplexed and sequenced in a single flow cell lane which is made possible due to a 4-nucleotide variable barcode sequence that was introduced in the adapter. Following 30 sequencing cycles, raw data is extracted, split into different samples based on the 4-nucleotide-barcode sequence and stripped from the barcode and four nucleotides of the adapter sequence. This resulted in 5-15x10^6^ *S. pneumoniae* specific reads per flow cell lane.

**Data analysis and fitness calculation**

Following sequencing, reads are mapped to the *S. pneumoniae* TIGR4 genome with the program Bowtie . Bowtie parameters (-m 1 –n 1 –best) were set such that reads could contain a single mismatch but were only allowed if they mapped to a unique location. If mapping to multiple sites was possible the read was excluded from the analyses. On average 8% of the reads had to be discarded for two reasons: i) 6% of these ‘reads’ could not be mapped to a single location. These reads map to multiple sites such as endogenous transposon related genes or other repeated sequences, ii) the remaining reads (2%) did not map anywhere and were categorized as junk sequences. Insertions that mapped to a location in the first 5% and the last 10% of a gene were removed from the analysis to minimize the influence of truncated functional genes. For two time points (t_1_ and t_2_) the number of reads at each location was recorded. On average 250 reads were mapped per insertion/time point. Since insertions with a very low number of reads that slightly fluctuate over time can influence the data disproportionately, only insertions with fifteen or more reads at t_1_ are included in the analyses. Subsequently the data is normalized by equalizing the total number of sequenced reads per time point (normalization factors for all data sets are small and lie between 0.92 and 1.06). The change over time in the number of reads at a specific location is then used to calculate fitness. Thus for each insertion, fitness *W_i_*, is calculated by comparing the fold expansion of the mutant relative to the rest of the population with the following equation :

$$W_{i}=\frac{\ln[N_{i}(t_{2})*d/N_{i}(t_{1})]}{\ln[(1-N_{i}(t_{2}))*d/(1-N_{i}(t_{2}))]}$$

In which *N_i_*(*t_1_*) and *N_i_(t_2_)* are the frequency of the mutant in the population at the start and at the end of the experiment, respectively, and *d* (expansion factor) represents the growth of the bacterial population during library selection. The growth or expansion factor was directly measured for each library by plating appropriate dilutions of the bacterial library at several time points during infection. After fitness was calculated for each insertion, values are normalized against a set of ‘neutral’ genes; genes that have no fitness effect in *S. pneumoniae*. These genes consist of pseudo genes and degenerate transposon related sequences. The same factor was then used to normalize the rest of the dataset and make all fitness values relative to the wt background. The normalization factors used for all datasets were small and lie between 0.98 and 1.09. Finally all of the insertions in a gene are used to calculate a gene’s average fitness and standard deviation. To further control for deviations in fitness due to insertions with small numbers of reads a weighted average is used. This means that insertions with less than 50 reads receive a proportional lower weight. This slightly increases correlations between replicates and it slightly lowers standard deviation.

Since we are determining *in vivo* fitness it is expected that due to a bottleneck effect not the complete dose and therefore not all insertion mutants contribute to establishing the infection. In order to be able to calculate accurate fitness values this random loss of mutants thus has to be taken into account in the analysis. The bottleneck for each mouse was calculated by determining the proportion of insertion mutants that were lost from the neutral set of genes. Since these genes have no effect on fitness, we assume that all insertions that are lost from this set are lost due to (stochastic) processes that occur during or shortly after the infection procedure. To remove the bottleneck effect from each gene’s fitness value the same proportion of insertions mutants that disappeared during *in vivo* selection are removed from each gene’s total number of insertions. The resulting set of insertions is then reanalyzed and fitness is re-calculated. Finally, the resulting *W_i_* for each gene represents the growth rate per generation and enables direct comparisons between experiments.

To determine whether a gene’s fitness between wt and sc mice significantly differed, three requirements had to be fulfilled: i) fitness had to be composed of at least four data points, ii) fitness had to deviate by at least 20%, and iii) fitness had to be significantly different in a one sample *t*-test with Bonferroni correction.

**Table S**1. Strains used in this study.

| S. pneumoniae strains used | Characteristics | Source |
| --- | --- | --- |
| TIGR4 | wild type encapsulated | [www.tigr.org](http://www.tigr.org/) |
| TIGR4R | wild type unencapsulated:Cm^R^ |  |
| CbpR- | TIGR4R:Erm^R^ | This study |
| RR03-/GRR- | TIGR4R:Erm^R^ | This study |
| VncR- | TIGR4R:Erm^R^ | (Haas, 2004), This study |
| sRNA R6- | TIGR4/TIGR4R:Erm^R^ | This study |
| sRNA R12- | TIGR4/TIGR4R:Erm^R^ | This study |
| sRNA F5- | TIGR4/TIGR4R:Erm^R^ | This study |
| sRNA F6- | TIGR4/TIGR4R:Erm^R^ | This study |
| sRNA F7- | TIGR4/TIGR4R:Erm^R^ | This study |
| sRNA F20- | TIGR4/TIGR4R:Erm^R^ | This study |
| sRNA F22- | TIGR4/TIGR4R:Erm^R^ | This study |
| sRNA F24- | TIGR4/TIGR4R:Erm^R^ | This study |
| sRNA F25- | TIGR4/TIGR4R:Erm^R^ | This study |
| sRNA F32- | TIGR4/TIGR4R:Erm^R^ | This study |
| sRNA F41- | TIGR4/TIGR4R:Erm^R^ | This study |
| sRNA F42- | TIGR4/TIGR4R:Erm^R^ | This study |
| sRNA F43- | TIGR4/TIGR4R:Erm^R^ | This study |
| sRNA F44- | TIGR4/TIGR4R:Erm^R^ | This study |
| sRNA F48- | TIGR4/TIGR4R:Erm^R^ | This study |
| sRNA F55- | TIGR4/TIGR4R:Erm^R^ | This study |
| sRNA F62- | TIGR4/TIGR4R:Erm^R^ | This study |

**Table S2. Primers used in this study.**

| **Primer** | **Sequence (5'-3')** | **Application** |
| --- | --- | --- |
| CbpRupf | CAGATAGAGCTGACGTGG | PCR mediated mutagenesis |
| CbpRupr | GTTTGCTTCTAAGTCTTATTTCCCCTCTTGTCCATCCTTAGC | PCR mediated mutagenesis |
| CbpRdnf | GAGTCGCTTTTGTAAATTTGGGAGAGCAGATATTGGAAG | PCR mediated mutagenesis |
| CbpRdnr | CATACCGTGTTCATAAGG | PCR mediated mutagenesis |
| RR03upf | CAAGAGTAACAAGAGTGTC | PCR mediated mutagenesis |
| RR03upr | GTTTGCTTCTAAGTCTTATTTCCCTACATCGTCTTGGAGGTC | PCR mediated mutagenesis |
| RR03dnf | GTCGCTTTTGTAAATTTGGAGGAGCTAGAAGCAGC | PCR mediated mutagenesis |
| RR03dnr | GCGCTTTTCAATCACGC | PCR mediated mutagenesis |
| VncRupf | TTCACACTGCTGCAAAAC* | PCR mediated mutagenesis |
| VncRdnr | GATGGGTCAAACTGTTGTC* | PCR mediated mutagenesis |
| R6upf | GAGATGTTCATCTCGGTACACC | PCR mediated mutagenesis |
| R6upr | CTAAGTCTTATTTCCGAGGTGGCACCGCGTTACC | PCR mediated mutagenesis |
| R6dnf | CTTTTGTAAATTTGGCATTCGAATATGACCATGTC | PCR mediated mutagenesis |
| R6dnr | GTATAGTAATATTAGAATGTTC | PCR mediated mutagenesis |
| R12upf | CCCTTTCAGTTCTACTTCTAG | PCR mediated mutagenesis |
| R12upr | CTAAGTCTTATTTCCGGACCTAGCTGCGATTGCAG | PCR mediated mutagenesis |
| R12dnf | CTTTTGTAAATTTGGTTTCGTATTAGTCCAGCC | PCR mediated mutagenesis |
| R12dnr | CAGGGAGCCTGTGGTGATTG | PCR mediated mutagenesis |
| F6upf | CTGGCTCAAGAACCATACCG | PCR mediated mutagenesis |
| F6upr | CTAAGTCTTATTTCCCACTACACTTGACAAAGAGCC | PCR mediated mutagenesis |
| F6dnf | CTTTTGTAAATTTGGCAAAGGCTTTGCGCTTGATAATC | PCR mediated mutagenesis |
| F6dnr | GACCGAAAATCCTTGGAGTATC | PCR mediated mutagenesis |
| F7upf | GTTGAGTGTTATGATGATGG | PCR mediated mutagenesis |
| F7upr | CTAAGTCTTATTTCCCGAATTGGTGCTGGGATTTG | PCR mediated mutagenesis |
| F7dnf | CTTTTGTAAATTTGGGAAGCAAACGGAGGACGG | PCR mediated mutagenesis |
| F7dnr | CCACATCAAAAACAGCCG | PCR mediated mutagenesis |
| F20upf | GGTAATATTATAATGGAGG | PCR mediated mutagenesis |
| F20upr | CTAAGTCTTATTTCCCCATTCTTGCAAGACCTATC | PCR mediated mutagenesis |
| F20dnf | CTTTTGTAAATTTGGGGAAATATGTCAACTATTG | PCR mediated mutagenesis |
| F20dnr | CAACACCTCAACATCTGCC | PCR mediated mutagenesis |
| F22upf | GAAGTCTGAGATCTTATGC | PCR mediated mutagenesis |
| F22upr | CTAAGTCTTATTTCCGGGCACCTTCTAAATGAAG | PCR mediated mutagenesis |
| F22dnf | CTTTTGTAAATTTGGGACTCCTAATCGTGGTATAC | PCR mediated mutagenesis |
| F22dnr | CCTATTATAACACATTTATC | PCR mediated mutagenesis |
| F24upf | GTTAAAGACGGTGACATCATG | PCR mediated mutagenesis |
| F24upr | CTAAGTCTTATTTCCCTAACCCTTTCAATCAATTC | PCR mediated mutagenesis |
| F24dnf | CTTTTGTAAATTTGGGGCATTGCGCTTGATAAG | PCR mediated mutagenesis |
| F24dnr | CACGGATAGTTCCTAATCTGG | PCR mediated mutagenesis |
| F25upf | CGATTTGATTGTCCTTATCG | PCR mediated mutagenesis |
| F25upr | CTAAGTCTTATTTCCCCATTTAGAAAGGCTATCCC | PCR mediated mutagenesis |
| F25dnf | CTTTTGTAAATTTGGCCCTAGAAAATGCTTAGGG | PCR mediated mutagenesis |
| F25dnr | GATTTTAATGAAATTAGCATC | PCR mediated mutagenesis |
| F32upf | GATGGACATAAATTCTTGCCC | PCR mediated mutagenesis |
| F32upr | GAGTCGCTTTTGTAAATTTGGGTGGGTTCGACTCCCACCGGC | PCR mediated mutagenesis |
| F32dnf | GTTTGCTTCTAAGTCTTATTTCCGAAATCACATACCTAAGATG | PCR mediated mutagenesis |
| F32dnr | CGATAGCGCGTTTGAGTGGGG | PCR mediated mutagenesis |
| F41upf | GTTAAATTGATGATTTAGGAGG | PCR mediated mutagenesis |
| F41upr | CTAAGTCTTATTTCCGAATATTATACCACACAATC | PCR mediated mutagenesis |
| F41dnf | CTTTTGTAAATTTGGGGTTATAATTGTTATTAGG | PCR mediated mutagenesis |
| F41dnr | CACCTAAACTTTCTAAACCTGC | PCR mediated mutagenesis |
| F42upf | GAAAGGAAATAACATGGAGG | PCR mediated mutagenesis |
| F42upr | CTAAGTCTTATTTCCCATACTACAACAACAAATC | PCR mediated mutagenesis |
| F42dnf | CTTTTGTAAATTTGGGGAGTTTACTGTATCAAATC | PCR mediated mutagenesis |
| F42dnr | CGTAAAATCATAAACTATCC | PCR mediated mutagenesis |
| F43upf | GTTTCAATGTAGCATCTTAC | PCR mediated mutagenesis |
| F43upr | CTAAGTCTTATTTCCCAACAGAAAACTGGAAATTC | PCR mediated mutagenesis |
| F43dnf | CTTTTGTAAATTTGGGACTTTGGGCTCTACTAGG | PCR mediated mutagenesis |
| F43dnr | CTAGCTTCAAACCGTGTTC | PCR mediated mutagenesis |
| F44upf | CCTTTCTGCTTTTAGATTAG | PCR mediated mutagenesis |
| F44upr | CTAAGTCTTATTTCCCCTTTGCTTAGGATAGTATAG | PCR mediated mutagenesis |
| F44dnf | CTTTTGTAAATTTGGCCTATTGCTGTAAAATAATGG | PCR mediated mutagenesis |
| F44dnr | GACAAGAGATTGATGCAAATC | PCR mediated mutagenesis |
| F48upf | CTCTTAGTCAATAAGATTAAG | PCR mediated mutagenesis |
| F48upr | CTAAGTCTTATTTCCGCTCTTTGATTTTCATTGAG | PCR mediated mutagenesis |
| F48dnf | CTTTTGTAAATTTGGCATTTTGAAATCTATTTACC | PCR mediated mutagenesis |
| F48dnr | CAATAAGGACAATCTCCGCCC | PCR mediated mutagenesis |
| F55upf | GAGGGCTTGGCAGAACAAC | PCR mediated mutagenesis |
| F55upr | CTAAGTCTTATTTCCGATCTGACTTCGTCAGTTC | PCR mediated mutagenesis |
| F55dnf | CTTTTGTAAATTTGGGGGCTGCAAGAACTCCACC | PCR mediated mutagenesis |
| F55dnr | CTGTTTCAGGTGCTCACTTG | PCR mediated mutagenesis |
| F5upF | GACTATCAGAAAAGAGGTAAATTTAG | PCR mediated mutagenesis |
| F5upR | GTTGCTTCTAAGTCTTATTTCATGATTTAATTTAATCGGC | PCR mediated mutagenesis |
| F5downF | GAGTCGCTTTTGTAAATTTGGGGAATGAAGATAAGAAGAAGG | PCR mediated mutagenesis |
| F5downR | GTTATTATTGTAGCAAATTGAG | PCR mediated mutagenesis |
| F62upF | GCAAGAAAACGAAAGGAGAGG | PCR mediated mutagenesis |
| F62upR | GTTGCTTCTAAGTCTTATTTCTATTATCCCTAATTGAAAATTG | PCR mediated mutagenesis |
| F62downF | GAGTCGCTTTTGTAAATTTGGGGTGAGACTACTCTCGGATTG | PCR mediated mutagenesis |
| F62downR | GTGTGTCTAAGCGACGCG | PCR mediated mutagenesis |
| R11probe | CAAATTCAAACCACGTCAGCATCGCCTTACCGTAGGTATGGTTACTGACTTC | Northern blot analysis |
| R12probe | GATGAAGAGATGTTCATCTCGGTACACCTTTATACTCTTCGAAAATCTCTTC | Northern blot analysis |
| R13probe | CCGTTGCCTTTTTGGATATAAGAGACTATTGTCCCAGACTC | Northern blot analysis |
| R14probe | CCCTTTTTGATTTTAAATGAGTATGAAAAGAGAATTTTTTGGCTCTTTGTC | Northern blot analysis |
| R16probe | CTATATTATGCGTTCTTTTAATATCAAAGACTTTTTGAC | Northern blot analysis |
| R17probe | CTAGTTTGCTCTTTGATTTTCATTGAGTATTACAGTGAACAATTTTTC | Northern blot analysis |
| R18probe | CAAATGTTCCCGTTTTAATCCCAGTTACAGTTTTTAATCCTCTGTAACCGTTG | Northern blot analysis |
| R19probe | CTTTATTTTGCCCTACTTGGCTCCCACTATCCCAAGCTCGCTTGAAGAAC | Northern blot analysis |
| R20probe | GGGCCATCCTAACCAGACAGCCACGAGAACAAGGGCTGTGCC | Northern blot analysis |
| F1probe | GAGAACGTCCCAAAGTGCTTAATATCAAGGCCTATCTTATTCAATAAAA | Northern blot analysis |
| F3probe | GTTCAAGTCCAAACTATTCTTTATAGAAAAGTCCGATAAATTCGAGC | Northern blot analysis |
| F4probe | CAAAAAACGCAAGCTTAAGCCTGCGGACAATCTTTATAGCCATTTCATTTC | Northern blot analysis |
| F6probe | GAGAGTACGAATTTCGTACTCTCCAGATTGCAGCTTTTCGCCTACCCACTAC | Northern blot analysis |
| F7probe | GGAGTTCATTAAATAAAGATATTAGATGAAAATCAAATTCAAACTAATTC | Northern blot analysis |
| F10probe | CACCATTTTTGATGCGTTTTTCATTATTCTGAATGCAATGTTCTATCTG | Northern blot analysis |
| F11probe | GTTGTAGGTGCTTATTTCACAATTTCAATGTGACCAGTGATAACGAATACC | Northern blot analysis |
| F14probe | CTGTCCAAATCATACCAATTATGAACGGTATTACAAATTTATATGTTTG | Northern blot analysis |
| F15probe | CACGTCAGCGTCGGCTTGTCATGGGTATGGTTACTGACTTCGTCAGTTCTA | Northern blot analysis |
| F18probe | GAAAGGACGAAATTTGTCCTTTCTCGAACTTAGCTTTTCTTCAACCCAC | Northern blot analysis |
| F19probe | GATTAAATAGGTTCCTGCTCACTTGTATTAGTGATAACATCCTTTTATTTC | Northern blot analysis |
| F20probe | CCTGATATACTTCCCTTGGGCTACTAGTCTTTCAGATTCCTATTCAATTAC | Northern blot analysis |
| F22probe | CATTATAACCTTGTTCTAATAGGAATTGGCAAGCACGCGCCGATCTC | Northern blot analysis |
| F24probe | GTCTCATCACCTCTATTAGCCTTTAAATTTTGATAGACTCTATTTTC | Northern blot analysis |
| F25probe | GTCTTGGTAAAATAGAATTGCCCAATAAACCATTTAGAAAGGCTATCCCATGC | Northern blot analysis |
| F26probe | CCACCCTTTCACCGTCTAACCTTAGCTGCCTCTCTGGACTGCTTTAAAGG | Northern blot analysis |
| F35probe | CATTCTTTTATCTGACACCTTGTGAGTCTCTCTGGACTCCCCTTAAAGGTTG | Northern blot analysis |
| F36probe | GTTTTAGAAGTTACCAAAATCTGACTACATCTTGTAATCAGATTTTATCG | Northern blot analysis |
| F39probe | CACAAAATAAGACTCTTTAAAGAATCTTTAAATTCATTAGGTGTGCTGCTTAC | Northern blot analysis |
| F40probe | GCACTAAGGGAAAGCGCCCCAAAGTGCTTATTTCAAAGGCTTTATAG | Northern blot analysis |
| F41probe | GGCTACCTCTATCAAGGTGTACTCCTTCTATACTATCCCTTGTGCTTTAG | Northern blot analysis |
| F42probe | GGTTTTCTAAATAAATCAAACTGATCATTTACATAACATAAATTATGTAAATAC | Northern blot analysis |
| F43probe | CTTTATTTGATAGACTTAGTTGTTCATGTCTCCCTTACCTCCGAGTATTAG | Northern blot analysis |
| F44probe | GATTTTAAAGAGTTTTAAATATTTCTGCGTCTTCGTCACCTCACGGGATGAC | Northern blot analysis |
| F45probe | GGTTTTTGCACCTTACATTATGCGTTTTTGTGATTTTAAGACTTGTTAGC | Northern blot analysis |
| F47probe | GGCCACCGTTTTATGTTTTCATAAAAAACTCAAGAGTAGTATCAGTTTAG | Northern blot analysis |
| F48probe | CAAACCGCGTCAGCTTTCATCTGCAACCTCAAAACAGTGTTTTGAGCAAC | Northern blot analysis |
| F51probe | CAAAAAGCCCTATAATAAGGGCTAAGTGCATTTAGGAGACTATACTTC | Northern blot analysis |
| F53probe | CTTTATCTGCAACCTCAAAGCTGTGCTTTGAGCAACCTACGGCTAGCTTCCT | Northern blot analysis |
| F55probe | CTTCAAACCACGTCAGCTTCATCTTGCCGTAGTATGGTTACTGACTTCGTCA | Northern blot analysis |
| F56probe | CAAACGCTTTATAAACTTATTCAGCTTCAACGCCACTTTTCATACCTATC | Northern blot analysis |
| F57probe | CTTGAAGCAAACGTCTCAAATCCTTTGTAATTCTTACTTTACAGCTAT | Northern blot analysis |
| F58probe | CAAAAAAGGAGATTTTACAATCTCCTCATAA | Northern blot analysis |
| F60probe | CTAACTTTTGAGAGAACTTCATTTTTGATTCAGACTTTTTCTACTGC | Northern blot analysis |
| F61probe | GTTATGACACGAAACCAACAAGCAAGTTCAAGCACCTCCATATCTTT | Northern blot analysis |
| F62probe | GATTAATCAGAAACCTCTGACTAAGATTTCCTAATTAATTCACTTTCTAT | Northern blot analysis |
| F63probe | CCGTCATATTATGAACCAAAAGAACTAAACTACCAACAGC | Northern blot analysis |
| F64probe | GAATTATGTTATGACATAGTAGATTTGATTTTATCAGTGCTGCTTAGG | Northern blot analysis |
| F65probe | CTTTGAAATCAGTGTTTCAGAGCATTTTTTGTTCTTTCTGTAAATAATTTG | Northern blot analysis |
| F66probe | CTCATTAGTTTCACCTCCTCTCACGAACCCATAGGAACGTAATCGGTAAC | Northern blot analysis |
| F67probe | CATCTGCTATAATAGTAAGAGCAAGCTCTGGTATAAGGCTCATTAGTTTCAC | Northern blot analysis |
| F68probe | CAAAAAGCCCTATAATAAGGGCTAAGTGCATTTAGGAGACTATACTTCAAATT | Northern blot analysis |
| F69probe | CAAAGTTTGATAGCTATTTGGATAGCAGTCAGGCTTTGATTTTTTACAGTC | Northern blot analysis |
| F70probe | GAATCTATAGTCTTTGACACAGAGGAAGAGTTTAGCTTGTTGATAATTCT | Northern blot analysis |
| F71probe | GAAAAGAATCCAACCACAGCATGGACTATTATATAGCAGATTGAAATAAG | Northern blot analysis |
| F72probe | CTGACGAAGTCAGCTCAAAACACTGTTTTGAGGTTGTGGATAGAAC | Northern blot analysis |
| F73probe | GTGATAAAGGAGAAATAAAGATGGCAGAAATTTATCTAGCAGGTGGTTG | Northern blot analysis |
| F74probe | GTTTTTAAGATTTTAAAGACTTTTTTCCTTTATCTGGTATTTTGACTAC | Northern blot analysis |
| R22probe | CTAGGAAGCTAGCCGCAGGTTGCTCAAAACACTGTTTTGAGGTTGTAGATG14 | Northern blot analysis |
| R23probe | CAAGCCTGACGATAGCAAGAACTACCCTACTCGATAGGTATCGGCTTTTGC | Northern blot analysis |
| R24probe | GAACTGACGAAGTCAGCTCAAAACACTGTTTTGAGGTTGCAGATAG | Northern blot analysis |
| R25probe | GTTTTATAATTTTAAAGACTTTTTTCTATAGTAGATTGAAATAAGATGCGAAC | Northern blot analysis |
| R26probe | CTAATTGTAAACTGTGATGGATACTTAACTTTGTATAATAGGTGGATAAAAGTCTTCAC | Northern blot analysis |
| R27probe | GATGTTAAAGACTTTCTACCAGGTTTTTTAAAAGCATAATTGTTAGTTG | Northern blot analysis |
| R28probe | GTTAGATTTTTTCTGTCTAACTTTTGGGGGGCAGTACAAAAGCACTC | Northern blot analysis |
| R29probe | GTTTTATTGTGGGAAGATTTACTTCATTTTCTCTTGAAATTGAGTTTTCTCC | Northern blot analysis |
| R30probe | GTTTGATTGTGGGAAGATTGACTTCATTTTCTCCTGAAATTGAAGTTTTTTC | Northern blot analysis |
| R31probe | GTTTTTCTATATCAAAGACTTTTTGCCCAATTCCTCAAAGCTATGCATTGAG | Northern blot analysis |
| R32probe | GTTAGATTTTTTCTGTCTAACTTTTGGGGGCAGTTCATAAGAACCTTGG | Northern blot analysis |
| Sp0239-F | TTGGGGCAGCGACAGATGCG | qRT-PCR |
| Sp0239-R | CCGTCTCAGCCAAAGCGCGA | qRT-PCR |
| Sp0240-F | CGATTGGTCGCGCGCAAGGT | qRT-PCR |
| Sp0240-R | GGGATTTCCCCCTGCAAGCCA | qRT-PCR |
| Sp0578-F | ACCCAAAGGTGGATTAGGAGAAGCA | qRT-PCR |
| Sp0578-R | CAACCCAGCTTCGTTAGGTTCTTCC | qRT-PCR |
| Sp0579-F | AGGCGCGTGCTATGGATGCC | qRT-PCR |
| Sp0579-R | CTGTGGGTCGCATCGTCCGT | qRT-PCR |
| Sp0625-F | AGGTGCCCACAACCTACCGCT | qRT-PCR |
| Sp0625-R | GCAAGCACGCGCCGATCTCA | qRT-PCR |
| Sp0626-F | GGACGCCCTTGCCTCAGTGG | qRT-PCR |
| Sp0626-R | TCGCTTCAGCTGGTACTGGGA | qRT-PCR |
| Sp0649-F | GCCGACGAACCTGAATCAGCCC | qRT-PCR |
| Sp0649-R | TCTGCCCAAAGACAGGTTCCACA | qRT-PCR |
| Sp0650-F | GGACGAAACTTTGCGGATTTGGCT | qRT-PCR |
| Sp0650-R | GGCTAGCCCCAGTCTAGGGAGGA | qRT-PCR |
| Sp0873-F | TGGGGAGCGTCTTGTCAGCTCTT | qRT-PCR |
| Sp0873-R | AGCCTGCAATATGCAAGCGATCC | qRT-PCR |
| Sp0874-F | GTGACTCCCTTTTTAGCAAAAGAATGC | qRT-PCR |
| Sp0874-R | TTATTCCTTTGCATTCTTTTGCATTCC | qRT-PCR |
| Sp1285-F | ATCGGGGCTGGGGCAGGATT | qRT-PCR |
| Sp1285-R | ATCTTCGGCACGTCCGTGGT | qRT-PCR |
| Sp1286-F | GCCATCGCTATCTTGCCAATCGCT | qRT-PCR |
| Sp1286-R | GGCTGGTCCACCAAGGAAGGC | qRT-PCR |
| Sp1142-F | TGTTATCGGAGCTTGTCAGCGGAT | qRT-PCR |
| Sp1142-R | CCCCCATTTTGGGCAAGGGTACA | qRT-PCR |
| Sp1143-F | AGGATAAAAACGGCAATGAGCCTGT | qRT-PCR |
| Sp1143-R | TTCGCCTGCGCGTGTTCCAT | qRT-PCR |
| Sp1872-F | GACCTCGGCGCTGCGGATAC | qRT-PCR |
| Sp1872-R | AATCAGGCTCAAGGGCGGCG | qRT-PCR |
| Sp1873-F | AGCGCCTGTGCGCTTTTACCA | qRT-PCR |
| Sp1873-R | GCGGTCTGGAACGGGGTCCT | qRT-PCR |
| gyrA-F | GTCAAGCGCGACGCCTCAGCC | qRT-PCR |
| gyrA-R | GCGCGCGCTTCCGCTTTTTCCT | qRT-PCR |
| R2-F | TTTTAGAATATCTGATATTATACGTTTTTTG | qRT-PCR |
| R2-R | CACCTAATCCACTCAGACAGCTGAGTGGATTTTTCG | qRT-PCR |
| R14-F | ATATACATATCCGTAAAACGATAAATTCCC | qRT-PCR |
| R14-R | TTGACAAAGAGCCAAAAAATTCTCTTTTCATACTC | qRT-PCR |
| F3-F | TTGCTCGAATTTATCGGACTTTTCTATAAAGAATAG | qRT-PCR |
| F3-R | TCTTTTCAACGACAAGAAAGAGGCTGATAATCTACC | qRT-PCR |
| F39-F | AAGCAGCACACCTAATGAATTTAAAGATTC | qRT-PCR |
| F39-R | AAAAGACGATAGAGTTTTTAATTTCTATCGTCTGAG | qRT-PCR |
| F42-F | TATTTACATAATTTATGTTATGTAAATGATCAG | qRT-PCR |
| F42-R | AAAAAATAACCGATCTCATTCCTGAGAATCGG | qRT-PCR |
| F45-F | GCTAACAAGTCTTAAAATCACAAAAACGC | qRT-PCR |
| F45-R | ACAATAAAACGCATAATATCAAGGTTTTTGC | qRT-PCR |
| F22-F | TGAGATCGGCGCGTGCTTGC | qRT-PCR |
| F22-R | ACTCTCCTACCCAGTCCACACCAAGTAGG | qRT-PCR |

**Table S3.** Coordinates of the additional regions of the TIGR4 genome containing the conserved motifs identified in Supplementary Figure 4. If there was increased RNA signal from the Illumina reads, probes were designed outside of the flanking coding region and the motif and used to probe Northern Blots. All conserved motifs tested (17) revealed a strong signal of approximately 250-300 bp indicating a high probability of the sRNA being encoded in these regions.

| **Motif Coordinates 5'** | **Motif Coordinates 3'** | **Positive Matches** | **Downstream gene** | **Upstream gene** | **Northern Signal** | **sRNA designation** |
| --- | --- | --- | --- | --- | --- | --- |
| **Motif 1** |  |  |  |  |  |  |
| 834960 | 834925 | 36/36 | SP0885 | SP0886 |  |  |
| 1291043 | 1291078 | 36/36 | SP01367 | SP01368 | Yes | F69 |
| 1801258 | 1801293 | 36/36 | SP01894 | SP01895 | Yes | F70 |
| 2026591 | 2026556 | 36/36 | SP2112 | SP2113 | Yes | R22 |
| 2032174 | 2032209 | 36/36 | SP2120 | SP2121 |  |  |
| 82401 | 82436 | 35/36 | SP0075 | SP0076 |  |  |
| 463723 | 463688 | 35/36 | SP0482 | SP0483 |  |  |
| 1161620 | 1161585 | 35/36 | SP1228 | SP1229 |  |  |
| 1599122 | 1599087 | 35/36 | SP1694 | SP1695 | Yes | R23 |
| 1622114 | 1622079 | 35/36 | SP1717 | SP1718 |  |  |
| 1580183 | 1580150 | 33/34 | SP1680 | SP1681 |  |  |
| 144150 | 144182 | 32/33 | SP0145 | SP0146 | Yes | F71 |
| 1082294 | 1082326 | 32/33 | Within Sp1896 (antisense) | NA | Yes | F72 |
| 1386255 | 1386227 | 29/29 | SP1472 | SP1473 |  |  |
| 98049 | 98014 | 34/36 | SP0095 | SP0096 |  |  |
| 1281974 | 1282009 | 34/36 | SP1359 | SP1360 | Yes | F73 |
| 2069947 | 2069912 | 34/36 | SP2155 | SP2156 | Yes | R24 |
| **Motif 2** | Motif 2 |  |  |  |  |  |
| 167762 | 167786 | 24/25 | SP0173 | SP0174 |  |  |
| 1207972 | 1207948 | 24/25 | SP1274 | SP1275 | Yes | R25 |
| 370728 | 370705 | 23/24 | SP0391 | SP0392 | Yes | R26 |
| 13146874 | 1346892 | 22/25 | Sp1427 | SP1428 | Yes | R29 |
| 273645 | 273624 | 20/25 | SP0297 | SP0298 |  |  |
| 843896 | 843873 | 22/24 | SP0892 | SP0893 | Yes | R27 |
| 1151227 | 1151208 | 18/25 | SP1220 | SP1221 | Yes | R28 |
| 1437477 | 1437492 | 15/24 | SP1526 | SP1527 |  |  |
| **Motif 3** | Motif 3 |  |  |  |  |  |
| 766693 | 766674 | 19/20 | SP0812 | SP0813 |  |  |
| 1120679 | 1120698 | 19/20 | SP1182 | SP1183 |  |  |
| 1346863 | 1346844 | 19/20 | SP1427 | SP1428 | Yes | R29 |
| 1437474 | 1437493 | 19/20 | SP1526 | SP1527 |  |  |
| 1501069 | 1501088 | 19/20 | SP1596 | SP1597 |  |  |
| 1836345 | 1836326 | 19/20 | SP1929 | SP1930 | Yes | R30 |
| 1120670 | 1120652 | 18/19 | SP1182 | SP1183 | Yes | R31 |
| 1497991 | 1497973 | 18/19 | SP1592 | SP1593 |  |  |
| 1619402 | 1619384 | 18/19 | SP1715 | SP1716 | Yes | R32 |
| **Motif 4** | Motif 4 |  |  |  |  |  |
| 959154 | 959126 | 29/29 | SP1015 | SP1016 |  |  |
| 1195055 | 1195083 | 29/29 | SP1262 | SP1263 |  |  |
| 1364496 | 1364524 | 29/29 | SP1444 | SP1445 |  |  |
| 1538086 | 1538058 | 29/29 | SP1639 | SP1640 |  |  |
| 1595213 | 1595241 | 29/29 | SP1692 | SP1693 |  |  |
| 1707188 | 1707160 | 29/29 | SP1792 | SP1792 |  |  |
| 1791010 | 1791038 | 29/29 | SP1886 | SP1886 |  |  |
| 1820248 | 1820276 | 29/29 | SP1905 | SP1906 |  |  |

**Table S4. Raw data from the Tn-seq screen**. Mean is the average fitness of the knockout in the respective host site with a score of 1 indicating parity to the wild type. Standard deviation and standard error of the mean are SD and SEM respectively. Total is the total number of insertions, blank is the total insertions that disappear during the challenge, and blank removed is the total insertions that are removed from the analysis based on bottleneck calculations. The tissue represents the host site used for the infectious challenge. Fitness values could not be calculated if there was low abundance in the input pools (n.i.= no insertions; n.d.= not determined). Due to small size of the sRNAs, all insertions within a given predicted sRNA sequence were included in the analysis.

| **Locus** | **Mean** | **SD** | **SEM** | **Total** | **Blank** | **Blank Removed** | **Tissue** |
| --- | --- | --- | --- | --- | --- | --- | --- |
| F01-SP0085-SP0086 | 0.88 | 0.30 | 0.07 | 26 | 7 | 7 | Lung |
| F02-SP0103-SP0104 | 0.85 | 0.44 | 0.11 | 22 | 9 | 6 | Lung |
| F03-SP0115-SP0116 | n.d. |  |  | n.i. |  |  | Lung |
| F04-SP0116-SP0117 | 0.99 | 0.31 | 0.11 | 9 | 1 | 1 | Lung |
| F05-SP0117-SP0118 | 0.89 | 0.26 | 0.05 | 41 | 13 | 12 | Lung |
| F06-SP0129-SP0130 | n.d. |  |  | n.i. |  |  | Lung |
| F07-SP0239-SP0240 | 0.49 | 0.45 | 0.11 | 22 | 13 | 6 | Lung |
| F08-SP0256-SP0257 | n.d. |  |  | n.i. |  |  | Lung |
| F09-SP0257-SP0258 | n.d. |  |  | n.i. |  |  | Lung |
| F10-SP0311-SP0312 | 1.21 | 0.49 | 0.16 | 9 | 0 | 0 | Lung |
| F11-SP0312-SP0313 | 1.35 | 0.44 | 0.16 | 9 | 2 | 2 | Lung |
| F12-SP0464-SP0465 | n.d. |  |  | n.i. |  |  | Lung |
| F13-SP0493-SP0494 | 0.78 | 0.46 | 0.10 | 32 | 14 | 9 | Lung |
| F14-SP0518-SP0519 | 0.96 | 0.28 | 0.03 | 96 | 28 | 28 | Lung |
| F15-SP0519-SP0520 | 0.97 | 0.26 | 0.04 | 48 | 8 | 8 | Lung |
| F16-SP0560-SP0561 | 1.08 | 0.48 | 0.09 | 34 | 4 | 4 | Lung |
| F17-SP0564-SP0565 | 0.82 | 0.28 | 0.10 | 10 | 4 | 3 | Lung |
| F18-SP0571-SP0572 | n.d. |  |  | n.i. |  |  | Lung |
| F19-SP0575-SP0576 | 0.80 | 0.27 | 0.10 | 9 | 2 | 2 | Lung |
| F20-SP0578-SP0579 | 0.97 | 0.26 | 0.04 | 64 | 15 | 15 | Lung |
| F21-SP0586-SP0587 | n.d. |  |  | n.i. |  |  | Lung |
| F22-SP0625-SP0626 | n.d. |  |  | n.i. |  |  | Lung |
| F23-SP0640-SP0641 | 0.98 | 0.33 | 0.06 | 49 | 15 | 15 | Lung |
| F24-SP0641-SP0642 | 1.04 | 0.33 | 0.08 | 26 | 8 | 8 | Lung |
| F25-SP0649-SP0650 | 1.02 | 0.61 | 0.20 | 9 | 0 | 0 | Lung |
| F26-SP0700-SP0701 | 0.66 | 0.59 | 0.20 | 13 | 9 | 4 | Lung |
| F27-SP0718-SP0719 | 0.94 | 0.34 | 0.13 | 9 | 2 | 2 | Lung |
| F28-SP0749-SP0750 | n.d. |  |  | n.i. |  |  | Lung |
| F29-SP0788-SP0789 | 1.15 | 0.12 | 0.04 | 9 | 0 | 0 | Lung |
| F30-SP0788-SP0789 | n.d. |  |  | n.i. |  |  | Lung |
| F31-SP0863-SP0864 | n.d. |  |  | n.i. |  |  | Lung |
| F32-SP0873-SP0874 | 0.08 | 0.25 | 0.07 | 18 | 17 | 5 | Lung |
| F33-SP0910-SP0911 | n.d. |  |  | n.i. |  |  | Lung |
| F34-SP0958-SP0959 | n.d. |  |  | n.i. |  |  | Lung |
| F35-SP0962-SP0963 | n.d. |  |  | n.i. |  |  | Lung |
| F36-SP1000-SP1001 | n.d. |  |  | n.i. |  |  | Lung |
| F38-SP1012-SP1013 | 0.88 | 0.33 | 0.07 | 31 | 10 | 9 | Lung |
| F39-SP1029-SP1030 | n.d. |  |  | n.i. |  |  | Lung |
| F40-SP1128-SP1129 | n.d. |  |  | n.i. |  |  | Lung |
| F41-SP1142-SP1143 | 1.08 | 0.33 | 0.06 | 47 | 12 | 12 | Lung |
| F42-SP1157-SP1158 | 0.89 | 0.32 | 0.10 | 13 | 3 | 3 | Lung |
| F43-SP1281-SP1282 | n.d. |  |  | n.i. |  |  | Lung |
| F44-SP1285-SP1286 | n.d. |  |  | n.i. |  |  | Lung |
| F45-SP1499-SP1500 | 0.81 | 0.48 | 0.18 | 9 | 3 | 2 | Lung |
| F46-SP1546-SP1547 | 0.90 | 0.52 | 0.20 | 9 | 3 | 2 | Lung |
| F47-SP1629-SP1630 | n.d. |  |  | n.i. |  |  | Lung |
| F48-SP1872-SP1873 | n.d. |  |  | n.i. |  |  | Lung |
| F49-SP2112-SP2113 | 0.70 | 0.50 | 0.19 | 9 | 4 | 2 | Lung |
| F50-SP2168-SP2169 | n.d. |  |  | n.i. |  |  | Lung |
| F51-SP2213-SP2214 | 0.80 | 0.37 | 0.13 | 9 | 1 | 1 | Lung |
| F52-SP0041-SP0042 | 0.61 | 0.38 | 0.14 | 9 | 3 | 2 | Lung |
| F53-SP0619-SP0620 | n.d. |  |  | n.i. |  |  | Lung |
| F54-SP1516-SP1517 | 1.61 |  |  | 1 | 0 | 0 | Lung |
| F55-SP1777-SP1778 | n.d. |  |  | n.i. |  |  | Lung |
| F56-SP0162-SP0163 | 0.57 | 0.57 | 0.22 | 9 | 5 | 2 | Lung |
| F57-SP0584-SP0585 | n.d. |  |  | n.i. |  |  | Lung |
| F58-SP0757-SP0758 | n.d. |  |  | n.i. |  |  | Lung |
| F59-SP0915-SP0916 | n.d. |  |  | n.i. |  |  | Lung |
| F60-SP1004-SP1005 | 0.54 | 0.50 | 0.17 | 13 | 8 | 4 | Lung |
| F61-SP1029-SP1030 | 0.99 | 0.33 | 0.10 | 13 | 2 | 2 | Lung |
| F62-SP1059-SP1060 | 0.54 | 0.39 | 0.13 | 13 | 7 | 4 | Lung |
| F63-SP1154-SP1155 | 0.90 | 0.30 | 0.11 | 9 | 2 | 2 | Lung |
| F64-SP1179-SP1180 | 0.80 | 0.40 | 0.12 | 17 | 8 | 5 | Lung |
| F65-SP1431-SP1432 | n.d. |  |  | n.i. |  |  | Lung |
| F66-SP2168-SP2169 | 0.93 | 0.46 | 0.10 | 32 | 16 | 9 | Lung |
| F67-SP2168-SP2169 | 0.82 | 0.50 | 0.11 | 27 | 14 | 8 | Lung |
| F68-SP2213-SP2214 | n.d. |  |  | n.i. |  |  | Lung |
| R01-SP0178-SP0179 | 0.89 | 0.42 | 0.10 | 27 | 10 | 8 | Lung |
| R02-SP1286-SP1287 | n.d. |  |  | n.i. |  |  | Lung |
| R03-SP1355-SP1356 | n.d. |  |  | n.i. |  |  | Lung |
| R04-SP1431-SP1432 | 0.98 | 0.57 | 0.13 | 30 | 13 | 9 | Lung |
| R06-SP1820-SP1821 | 0.67 | 0.55 | 0.15 | 18 | 9 | 5 | Lung |
| R07-SP1886-SP1887 | n.d. |  |  | n.i. |  |  | Lung |
| R08-SP1988-SP1989 | 0.93 | 0.35 | 0.07 | 31 | 6 | 6 | Lung |
| R09-SP1999-SP2000 | n.d. |  |  | n.i. |  |  | Lung |
| R10-SP2076-SP2077 | n.d. |  |  | n.i. |  |  | Lung |
| R11-SP2093-SP2094 | 1.07 | 0.41 | 0.11 | 18 | 3 | 3 | Lung |
| R12-SP1818-SP1819 | 0.81 | 0.32 | 0.08 | 18 | 4 | 4 | Lung |
| R13-SP0649-SP0650 | n.d. |  |  | n.i. |  |  | Lung |
| R14-SP1100-SP1101 | 0.53 | 0.44 | 0.17 | 9 | 5 | 2 | Lung |
| R15-SP1278-SP1279 | n.d. |  |  | n.i. |  |  | Lung |
| R16-SP1292-SP1293 | 0.99 | 0.34 | 0.06 | 48 | 13 | 13 | Lung |
| R17-SP1356-SP1357 | n.d. |  |  | n.i. |  |  | Lung |
| R18-SP1444-SP1445 | n.d. |  |  | n.i. |  |  | Lung |
| R19-SP1477-SP1478 | n.d. |  |  | n.i. |  |  | Lung |
| R20-SP1547-SP1548 | n.d. |  |  | n.i. |  |  | Lung |
| R21-SP1551-SP1552 | n.d. |  |  | n.i. |  |  | Lung |
| srn026-SP0145-SP0146 | n.i. |  |  |  |  |  | Lung |
| srn029-SP0178-SP0179 | 0.99 | 0.43 | 0.08 | 35 | 6 | 6 | Lung |
| srn049-rpmD-rplO | n.i. |  |  |  |  |  | Lung |
| srn061-SP0239-SP0240 | 0.49 | 0.45 | 0.11 | 22 | 13 | 6 | Lung |
| srn068-SP0272-SP0273 | 0.83 | 0.53 | 0.15 | 18 | 7 | 5 | Lung |
| srn081-SP0297-SP0298 | 0.95 | 0.32 | 0.07 | 23 | 5 | 5 | Lung |
| srn098-SP0372-SP0373 | n.i. |  |  |  |  |  | Lung |
| srn135-SP0461-SP0462 | 1.09 | 0.50 | 0.13 | 23 | 8 | 7 | Lung |
| srn141-SP0482-SP0483 | n.i. |  |  |  |  |  | Lung |
| srn145-SP0493-SP0494 | 0.94 | 0.21 | 0.05 | 22 | 5 | 5 | Lung |
| srn151-SP0519-SP0520 | 0.93 | 0.41 | 0.16 | 9 | 3 | 2 | Lung |
| srn157-SP0578-SP0579 | 0.53 | 0.33 | 0.13 | 9 | 3 | 2 | Lung |
| srn164-SP0629-SP0630 | 1.11 | 0.68 | 0.26 | 9 | 4 | 2 | Lung |
| srn176-SP0715-SP0716 | n.i. |  |  |  |  |  | Lung |
| srn206-SP0831-SP0832 | 1.08 | 0.18 | 0.03 | 47 | 12 | 12 | Lung |
| srn218-tRNAGln-rpsA | 0.15 | 0.34 | 0.13 | 9 | 8 | 2 | Lung |
| srn226-SP0873-SP0874 | 0.08 | 0.25 | 0.07 | 18 | 17 | 5 | Lung |
| srn231-SP0897-SP0899 | 1.12 | 0.22 | 0.06 | 13 | 0 | 0 | Lung |
| srn235-SP0915-SP0916 | 0.05 | 0.19 | 0.05 | 18 | 17 | 5 | Lung |
| srn239-SP0958-SP0959 | n.i. |  |  |  |  |  | Lung |
| srn241-SP0962-SP0963 | n.i. |  |  |  |  |  | Lung |
| srn249-SP0983-SP0984 | 0.98 | 0.16 | 0.08 | 4 | 0 | 0 | Lung |
| srn254-SP1012-SP1013 | 0.94 | 0.27 | 0.06 | 22 | 4 | 4 | Lung |
| srn266-SP1101-SP1102 | 1.11 | 0.48 | 0.13 | 18 | 9 | 5 | Lung |
| srn267-SP1104-rplU | n.i. |  |  |  |  |  | Lung |
| srn270-SP1107-SP1108 | n.i. |  |  |  |  |  | Lung |
| srn277-SP1142-SP1143 | 1.07 | 0.34 | 0.05 | 73 | 21 | 21 | Lung |
| srn279-SP1166-SP1167 | 0.74 | 0.49 | 0.10 | 31 | 16 | 9 | Lung |
| srn299-SP1278-SP1279 | n.i. |  |  |  |  |  | Lung |
| srn308-SP1355-SP1356 | n.i. |  |  |  |  |  | Lung |
| srn317-SP1415-SP1416 | 0.91 | 0.35 | 0.08 | 31 | 10 | 9 | Lung |
| srn319-SP1440-SP1441 | n.i. |  |  |  |  |  | Lung |
| srn351-SP1551-SP1552 | n.i. |  |  |  |  |  | Lung |
| srn395-SP1790-SP1791 | n.i. |  |  |  |  |  | Lung |
| srn400-SP1820-SP1821 | 0.31 | 0.37 | 0.14 | 9 | 6 | 2 | Lung |
| srn435-SP1963-SP1964 | 0.80 | 0.49 | 0.18 | 9 | 3 | 2 | Lung |
| srn448-SP1998-SP1999 | n.i. |  |  |  |  |  | Lung |
| srn477-SP2075-SP2076 | n.i. |  |  |  |  |  | Lung |
| srn478-SP2075-SP2077 | n.i. |  |  |  |  |  | Lung |
| srn491-SP2097-SP2098 | n.i. |  |  |  |  |  | Lung |
| srn502-SP2168-SP2169 | 0.93 | 0.46 | 0.10 | 32 | 16 | 9 | Lung |
| srn503-SP2168-SP2169 | 0.81 | 0.50 | 0.12 | 27 | 14 | 8 | Lung |
| srn142-SP0486-SP0487 | 0.68 | 0.50 | 0.12 | 22 | 11 | 6 | Lung |
| srn368-SP1629-SP1630 | 0.41 | 0.44 | 0.17 | 9 | 5 | 2 | Lung |
| trn0012-SP0019-SP0020 | 0.53 | 0.48 | 0.12 | 22 | 13 | 6 | Lung |
| trn0027-SP0071-SP0072 | 0.83 | 0.33 | 0.08 | 27 | 10 | 8 | Lung |
| trn0036-SP0086-SP0087 | 0.87 | 0.30 | 0.07 | 26 | 7 | 7 | Lung |
| trn0052-SP0117-SP0118 | 0.64 | 0.37 | 0.12 | 14 | 6 | 4 | Lung |
| trn0057-SP0120-SP0121 | n.i. |  |  |  |  |  | Lung |
| trn0093-SP0223-SP0224 | n.i. |  |  |  |  |  | Lung |
| trn0156-SP0311-SP0312 | 0.72 | 0.28 | 0.11 | 9 | 2 | 2 | Lung |
| trn0157-SP0311-SP0312 | 1.07 | 0.40 | 0.07 | 36 | 5 | 5 | Lung |
| trn0218-SP0411-SP0412 | n.i. |  |  |  |  |  | Lung |
| trn0256-SP0460-SP0461 | 1.22 | 0.37 | 0.10 | 15 | 2 | 2 | Lung |
| trn0273-SP0484-SP0486 | n.i. |  |  |  |  |  | Lung |
| trn0290-SP0516-SP0517 | n.i. |  |  |  |  |  | Lung |
| trn0329-SP0640-SP0641 | 0.87 | 0.36 | 0.07 | 40 | 17 | 12 | Lung |
| trn0332-SP0648-SP0650 | 1.02 | 0.61 | 0.20 | 9 | 0 | 0 | Lung |
| trn0335-SP0707-SP0709 | n.i. |  |  |  |  |  | Lung |
| trn0358-SP0718-SP0719 | n.i. |  |  |  |  |  | Lung |
| trn0423-SP0854-SP0855 | n.i. |  |  |  |  |  | Lung |
| trn0485-SP1004-SP1006 | 0.54 | 0.50 | 0.17 | 13 | 8 | 4 | Lung |
| trn0489-dapA-trmE | n.i. |  |  |  |  |  | Lung |
| trn0498-SP1045-SP1047 | n.i. |  |  |  |  |  | Lung |
| trn0500-SP1065-SP1067 | n.i. |  |  |  |  |  | Lung |
| trn0510-SP1080-SP1081 | n.i. |  |  |  |  |  | Lung |
| trn0573-SP1220-SP1221 | n.i. |  |  |  |  |  | Lung |
| trn0591-SP1256-SP1258 | n.i. |  |  |  |  |  | Lung |
| trn0634-SP1384-SP1385 | 0.17 | 0.29 | 0.11 | 9 | 7 | 2 | Lung |
| trn0663-SP1477-SP1478 | n.i. |  |  |  |  |  | Lung |
| trn0674-SP1490-SP1491 | 1.05 | 0.34 | 0.13 | 9 | 2 | 2 | Lung |
| trn0696-SP1514-SP1516 | n.i. |  |  |  |  |  | Lung |
| trn0723-SP1552-SP1553 | n.i. |  |  |  |  |  | Lung |
| trn0757-SP1612-SP1613 | 0.67 | 0.39 | 0.15 | 9 | 3 | 2 | Lung |
| trn0760-SP1625-SP1626 | 0.92 | 0.29 | 0.06 | 23 | 3 | 3 | Lung |
| trn0761-SP1625-SP1626 | 0.88 | 0.34 | 0.11 | 13 | 4 | 4 | Lung |
| trn0788-SP1692-SP1694 | n.i. |  |  |  |  |  | Lung |
| trn0830-SP1818-SP1819 | 0.81 | 0.32 | 0.09 | 18 | 4 | 4 | Lung |
| trn0866-SP1900-tRNASer |  |  |  |  |  |  | Lung |
| trn0883-SP1922-SP1923 | 0.93 | 0.44 | 0.16 | 9 | 2 | 2 | Lung |
| trn0884-SP1922-SP1923 | n.i. |  |  |  |  |  | Lung |
| trn0909-rpoB-tRNAcys | n.i. |  |  |  |  |  | Lung |
| trn0935-SP1999-SP2000 | n.i. |  |  |  |  |  | Lung |
| trn0978-SP2075-SP2077 | n.i. |  |  |  |  |  | Lung |
| trn1009-SP2103-SP2104 | 0.92 | 0.37 | 0.12 | 13 | 3 | 3 | Lung |
| trn1025-SP2136-SP2138 | 0.91 | 0.38 | 0.08 | 35 | 14 | 10 | Lung |
| trn1031-SP2153-SP2156 | n.i. |  |  |  |  |  | Lung |
| trn1052-SP2210-tsf | n.i. |  |  |  |  |  | Lung |
| SN1-SP0019-SP0020 | 0.62 | 0.47 | 0.08 | 49 | 24 | 15 | Lung |
| SN2-SP0041-SP0042 | 0.75 | 0.43 | 0.07 | 48 | 16 | 14 | Lung |
| SN3-SP0114-SP0115 | 0.93 | 0.35 | 0.03 | 144 | 43 | 43 | Lung |
| SN4-SP0178-SP0179 | 0.92 | 0.31 | 0.04 | 66 | 19 | 19 | Lung |
| SN5-SP0256-SP0257 | 0.34 | 0.35 | 0.08 | 27 | 17 | 8 | Lung |
| SN6-SP0257-SP0258 | 0.66 | 0.46 | 0.11 | 26 | 13 | 8 | Lung |
| SN7-SP0260-SP0261 | 0.79 | 0.45 | 0.10 | 31 | 14 | 9 | Lung |
| SN8-SP0372-SP0373 | 1.13 | 0.29 | 0.08 | 13 | 0 | 0 | Lung |
| SN9-SP0439-SP0440 | n.i. |  |  |  |  |  | Lung |
| SN10-SP0486-SP0487 | 0.68 | 0.50 | 0.12 | 22 | 11 | 6 | Lung |
| SN11-SP0649-SP0650 | 0.98 | 0.64 | 0.21 | 9 | 0 | 0 | Lung |
| SN12-SP0700-SP0701 | 0.49 | 0.48 | 0.12 | 22 | 14 | 6 | Lung |
| SN13-SP0715-SP0716 | 0.77 | 0.37 | 0.14 | 9 | 3 | 2 | Lung |
| SN14-SP0834-SP0835 | 0.93 | 0.74 | 0.28 | 9 | 4 | 2 | Lung |
| SN15-SP0873-SP0874 | n.i. |  |  |  |  |  | Lung |
| SN16-SP0873-SP0874 | 0.09 | 0.26 | 0.06 | 27 | 25 | 8 | Lung |
| SN17-SP0897-SP0898 | 1.11 | 0.34 | 0.04 | 113 | 30 | 30 | Lung |
| SN18-SP0898-SP0899 | 0.96 | 0.32 | 0.09 | 13 | 1 | 1 | Lung |
| SN19-SP0899-SP0900 | 0.97 | 0.26 | 0.07 | 18 | 4 | 4 | Lung |
| SN20-SP0915-SP0916 | 0.05 | 0.19 | 0.05 | 18 | 17 | 5 | Lung |
| SN21-SP1068-SP1069 | 1.11 | 0.22 | 0.05 | 27 | 6 | 6 | Lung |
| SN22-SP1100-SP1101 | 0.26 | 0.38 | 0.14 | 9 | 7 | 2 | Lung |
| SN23-SP1400-SP1401 | 0.95 | 0.28 | 0.05 | 45 | 13 | 13 | Lung |
| SN24-SP1629-SP1630 | 0.41 | 0.44 | 0.17 | 9 | 5 | 2 | Lung |
| SN25-SP1691-SP1692 | 1.00 | 0.33 | 0.04 | 107 | 24 | 24 | Lung |
| SN26-SP2078-SP2079 | 0.43 | 0.39 | 0.15 | 9 | 5 | 2 | Lung |
| SN27-SP2168-SP2169 | 0.88 | 0.48 | 0.07 | 59 | 27 | 18 | Lung |
| SN28-SP0502-SP0503 | n.i. |  |  |  |  |  | Lung |
| SN29-SP0516-SP0517 | 0.84 | 0.43 | 0.12 | 18 | 9 | 5 | Lung |
| SN30-SP0518-SP0519 | 0.70 | 0.45 | 0.10 | 27 | 15 | 8 | Lung |
| SN31-SP2092-SP2093 | 0.62 | 0.49 | 0.08 | 53 | 29 | 16 | Lung |
| SN32-SP1086-SP1087 | 0.08 | 0.18 | 0.07 | 9 | 8 | 2 | Lung |
| SN33-SP0411-SP0412 | n.i. |  |  |  |  |  | Lung |
| SN34-SP1790-SP1791 | n.i. |  |  |  |  |  | Lung |
| SN35-SP0239-SP0240 | 0.86 | 0.36 | 0.05 | 66 | 23 | 20 | Lung |
| SN36-SP0451-SP0452 | 0.81 | 0.31 | 0.10 | 9 | 0 | 0 | Lung |
| SN37-SP0587-SP0588 | n.i. |  |  |  |  |  | Lung |
| SN38-SP0502-SP0503 | 0.82 | 0.63 | 0.13 | 31 | 15 | 9 | Lung |
| SN39-SP0761-SP0762 | 1.18 | 0.34 | 0.08 | 18 | 0 | 0 | Lung |
| SN40-SP0958-SP0959 | n.i. |  |  |  |  |  | Lung |
| SN41-SP1104-SP1105 | n.i. |  |  |  |  |  | Lung |
| SN42-SP1278-SP1279 | n.i. |  |  |  |  |  | Lung |
| SN43-SP1355-SP1356 | 0.59 | 0.36 | 0.14 | 9 | 3 | 2 | Lung |
| SN44-SP1551-SP1552 | n.i. |  |  |  |  |  | Lung |
| SN45-SP2097-SP2098 | 1.30 | 0.30 | 0.15 | 4 | 0 | 0 | Lung |
| SN46-SP2136-SP2137 | 0.81 | 0.40 | 0.06 | 57 | 24 | 17 | Lung |
| SN47-SP0051-SP0052 | n.i. |  |  |  |  |  | Lung |
| SN48-SP1166-SP1167 | 0.69 | 0.51 | 0.12 | 27 | 15 | 8 | Lung |
| SN49-SP1547-SP1548 | 0.75 | 0.55 | 0.11 | 33 | 16 | 10 | Lung |
| SN50-SP1966-SP1967 | 1.04 | 0.32 | 0.05 | 49 | 15 | 15 | Lung |
| F01-SP0085-SP0086 | 0.918 | 0.319 | 0.184 | 9 | 7 | 6 | Nasopharynx |
| F02-SP0103-SP0104 | 0 |  |  | 3 | 3 | 2 | Nasopharynx |
| F03-SP0115-SP0116 | n.d. |  |  | n.i. |  |  | Nasopharynx |
| F04-SP0116-SP0117 | n.d. |  |  | n.i. |  |  | Nasopharynx |
| F05-SP0117-SP0118 | 0 |  |  | 2 | 2 | 1 | Nasopharynx |
| F06-SP0129-SP0130 | n.d. |  |  | n.i. |  |  | Nasopharynx |
| F07-SP0239-SP0240 | 0 |  |  | 3 | 3 | 2 | Nasopharynx |
| F08-SP0256-SP0257 | n.d. |  |  | n.i. |  |  | Nasopharynx |
| F09-SP0257-SP0258 | n.d. |  |  | n.i. |  |  | Nasopharynx |
| F10-SP0311-SP0312 | n.d. |  |  | n.i. |  |  | Nasopharynx |
| F11-SP0312-SP0313 | 0.995 | 0.075 | 0.043 | 5 | 2 | 2 | Nasopharynx |
| F12-SP0464-SP0465 | n.d. |  |  | n.i. |  |  | Nasopharynx |
| F13-SP0493-SP0494 | 0 |  |  | 2 | 2 | 1 | Nasopharynx |
| F14-SP0518-SP0519 | 0.139 | 0.341 | 0.129 | 24 | 23 | 17 | Nasopharynx |
| F15-SP0519-SP0520 | 1.047 | 0.042 | 0.016 | 16 | 9 | 9 | Nasopharynx |
| F16-SP0560-SP0561 | 0.992 | 0.291 | 0.130 | 16 | 12 | 11 | Nasopharynx |
| F17-SP0564-SP0565 | 0 |  |  | 3 | 3 | 2 | Nasopharynx |
| F18-SP0571-SP0572 | n.d. |  |  | n.i. |  |  | Nasopharynx |
| F19-SP0575-SP0576 | n.d. |  |  | n.i. |  |  | Nasopharynx |
| F20-SP0578-SP0579 | 0.430 | 0.456 | 0.204 | 18 | 16 | 13 | Nasopharynx |
| F21-SP0586-SP0587 | n.d. |  |  | n.i. |  |  | Nasopharynx |
| F22-SP0625-SP0626 | n.d. |  |  | n.i. |  |  | Nasopharynx |
| F23-SP0640-SP0641 | 0.706 | 0.507 | 0.293 | 9 | 7 | 6 | Nasopharynx |
| F24-SP0641-SP0642 | 0 |  |  | 6 | 6 | 4 | Nasopharynx |
| F25-SP0649-SP0650 | 0.966 | 0.043 | 0.030 | 6 | 4 | 4 | Nasopharynx |
| F26-SP0700-SP0701 | 0 |  |  | 3 | 3 | 2 | Nasopharynx |
| F27-SP0718-SP0719 | 0.937 | 0 | 0 | 2 | 1 | 1 | Nasopharynx |
| F28-SP0749-SP0750 | n.d. |  |  | n.i. |  |  | Nasopharynx |
| F29-SP0788-SP0789 | n.d. |  |  | n.i. |  |  | Nasopharynx |
| F30-SP0788-SP0789 | n.d. |  |  | n.i. |  |  | Nasopharynx |
| F31-SP0863-SP0864 | n.d. |  |  | n.i. |  |  | Nasopharynx |
| F32-SP0873-SP0874 | 0 |  |  | 2 | 2 | 1 | Nasopharynx |
| F33-SP0910-SP0911 | n.d. |  |  | n.i. |  |  | Nasopharynx |
| F34-SP0958-SP0959 | n.d. |  |  | n.i. |  |  | Nasopharynx |
| F35-SP0962-SP0963 | n.d. |  |  | n.i. |  |  | Nasopharynx |
| F36-SP1000-SP1001 | n.d. |  |  | n.i. |  |  | Nasopharynx |
| F38-SP1012-SP1013 | 0 |  |  | 12 | 12 | 8 | Nasopharynx |
| F39-SP1029-SP1030 | n.d. |  |  | n.i. |  |  | Nasopharynx |
| F40-SP1128-SP1129 | n.d. |  |  | n.i. |  |  | Nasopharynx |
| F41-SP1142-SP1143 | 0 |  |  | 14 | 14 | 10 | Nasopharynx |
| F42-SP1157-SP1158 | 1.026 | 0 | 0 | 3 | 2 | 2 | Nasopharynx |
| F43-SP1281-SP1282 | n.d. |  |  | n.i. |  |  | Nasopharynx |
| F44-SP1285-SP1286 | n.d. |  |  | n.i. |  |  | Nasopharynx |
| F45-SP1499-SP1500 | 1.006 | 0 | 0 | 2 | 1 | 1 | Nasopharynx |
| F46-SP1546-SP1547 | 0 |  |  | 2 | 2 | 1 | Nasopharynx |
| F47-SP1629-SP1630 | n.d. |  |  | n.i. |  |  | Nasopharynx |
| F48-SP1872-SP1873 | n.d. |  |  | n.i. |  |  | Nasopharynx |
| F49-SP2112-SP2113 | n.d. |  |  | n.i. |  |  | Nasopharynx |
| F50-SP2168-SP2169 | n.d. |  |  | n.i. |  |  | Nasopharynx |
| F51-SP2213-SP2214 | 0.466 | 0.422 | 0.243 | 9 | 8 | 6 | Nasopharynx |
| F52-SP0041-SP0042 | 0 |  |  | 6 | 6 | 4 | Nasopharynx |
| F53-SP0619-SP0620 | n.d. |  |  | n.i. |  |  | Nasopharynx |
| F54-SP1516-SP1517 | n.d. |  |  | n.i. |  |  | Nasopharynx |
| F55-SP1777-SP1778 | n.d. |  |  | n.i. |  |  | Nasopharynx |
| F56-SP0162-SP0163 | n.d. |  |  | n.i. |  |  | Nasopharynx |
| F57-SP0584-SP0585 | n.d. |  |  | n.i. |  |  | Nasopharynx |
| F58-SP0757-SP0758 | n.d. |  |  | n.i. |  |  | Nasopharynx |
| F59-SP0915-SP0916 | n.d. |  |  | n.i. |  |  | Nasopharynx |
| F60-SP1004-SP1005 | 0 |  |  | 2 | 2 | 1 | Nasopharynx |
| F61-SP1029-SP1030 | 1.026 | 0.097 | 0.068 | 3 | 1 | 1 | Nasopharynx |
| F62-SP1059-SP1060 | 0 |  |  | 3 | 3 | 2 | Nasopharynx |
| F63-SP1154-SP1155 | 1.144 | 0.034 | 0.015 | 5 | 0 | 0 | Nasopharynx |
| F64-SP1179-SP1180 | 0 |  |  | 6 | 6 | 4 | Nasopharynx |
| F65-SP1431-SP1432 | n.d. |  |  | n.i. |  |  | Nasopharynx |
| F66-SP2168-SP2169 | 0 |  |  | 8 | 8 | 5 | Nasopharynx |
| F67-SP2168-SP2169 | n.d. |  |  | n.i. |  |  | Nasopharynx |
| F68-SP2213-SP2214 | n.d. |  |  | n.i. |  |  | Nasopharynx |
| R01-SP0178-SP0179 | n.d. |  |  | n.i. |  |  | Nasopharynx |
| R02-SP1286-SP1287 | n.d. |  |  | n.i. |  |  | Nasopharynx |
| R03-SP1355-SP1356 | n.d. |  |  | n.i. |  |  | Nasopharynx |
| R04-SP1431-SP1432 | 0.933 | 0.087 | 0.050 | 8 | 5 | 5 | Nasopharynx |
| R06-SP1820-SP1821 | 0 |  |  | 3 | 3 | 2 | Nasopharynx |
| R07-SP1886-SP1887 | n.d. |  |  | n.i. |  |  | Nasopharynx |
| R08-SP1988-SP1989 | 0 |  |  | 6 | 6 | 4 | Nasopharynx |
| R09-SP1999-SP2000 | n.d. |  |  | n.i. |  |  | Nasopharynx |
| R10-SP2076-SP2077 | n.d. |  |  | n.i. |  |  | Nasopharynx |
| R11-SP2093-SP2094 | n.d. |  |  | n.i. |  |  | Nasopharynx |
| R12-SP1818-SP1819 | 0 |  |  | 5 | 5 | 3 | Nasopharynx |
| R13-SP0649-SP0650 | n.d. |  |  | n.i. |  |  | Nasopharynx |
| R14-SP1100-SP1101 | n.d. |  |  | n.i. |  |  | Nasopharynx |
| R15-SP1278-SP1279 | n.d. |  |  | n.i. |  |  | Nasopharynx |
| R16-SP1292-SP1293 | 0.430 | 0.430 | 0.215 | 13 | 11 | 9 | Nasopharynx |
| R17-SP1356-SP1357 | n.d. |  |  | n.i. |  |  | Nasopharynx |
| R18-SP1444-SP1445 | n.d. |  |  | n.i. |  |  | Nasopharynx |
| R19-SP1477-SP1478 | n.d. |  |  | n.i. |  |  | Nasopharynx |
| R20-SP1547-SP1548 | n.d. |  |  | n.i. |  |  | Nasopharynx |
| R21-SP1551-SP1552 | n.d. |  |  | n.i. |  |  | Nasopharynx |
| srn026-SP0145-SP0146 | n.i. |  |  |  |  |  | Nasopharynx |
| srn029-SP0178-SP0179 | 0.97 | 0.06 | 0.03 | 7 | 2 | 2 | Nasopharynx |
| srn049-rpmD-rplO | n.i. |  |  |  |  |  | Nasopharynx |
| srn061-SP0239-SP0240 | 0.00 |  |  | 3 | 3 | 2 | Nasopharynx |
| srn068-SP0272-SP0273 | 0.00 |  |  | 3 | 3 | 2 | Nasopharynx |
| srn081-SP0297-SP0298 | 0.00 |  |  | 2 | 2 | 1 | Nasopharynx |
| srn098-SP0372-SP0373 | n.i. |  |  |  |  |  | Nasopharynx |
| srn135-SP0461-SP0462 | 0.30 | 0.43 | 0.25 | 8 | 7 | 5 | Nasopharynx |
| srn141-SP0482-SP0483 | n.i. |  |  |  |  |  | Nasopharynx |
| srn145-SP0493-SP0494 | 0.00 |  |  | 3 | 3 | 2 | Nasopharynx |
| srn151-SP0519-SP0520 | 0.96 | 0.00 | 0.00 | 2 | 1 | 1 | Nasopharynx |
| srn157-SP0578-SP0579 | 0.73 | 0.37 | 0.21 | 9 | 7 | 6 | Nasopharynx |
| srn164-SP0629-SP0630 | 1.10 | 0.00 | 0.00 | 3 | 2 | 2 | Nasopharynx |
| srn176-SP0715-SP0716 | n.i. |  |  |  |  |  | Nasopharynx |
| srn206-SP0831-SP0832 | 0.65 | 0.44 | 0.22 | 15 | 12 | 11 | Nasopharynx |
| srn218-tRNAGln-rpsA | n.i. |  |  |  |  |  | Nasopharynx |
| srn226-SP0873-SP0874 | 0.00 |  |  | 2 | 2 | 1 | Nasopharynx |
| srn231-SP0897-SP0899 | 1.05 | 0.05 | 0.02 | 9 | 4 | 4 | Nasopharynx |
| srn235-SP0915-SP0916 | 0.00 |  |  | 2 | 2 | 1 | Nasopharynx |
| srn239-SP0958-SP0959 | n.i. |  |  |  |  |  | Nasopharynx |
| srn241-SP0962-SP0963 | n.i. |  |  |  |  |  | Nasopharynx |
| srn249-SP0983-SP0984 | 0.00 |  |  | 2 | 2 | 1 | Nasopharynx |
| srn254-SP1012-SP1013 | 0.00 |  |  | 10 | 10 | 7 | Nasopharynx |
| srn266-SP1101-SP1102 | 0.00 |  |  | 2 | 2 | 1 | Nasopharynx |
| srn267-SP1104-rplU | n.i. |  |  |  |  |  | Nasopharynx |
| srn270-SP1107-SP1108 | n.i. |  |  |  |  |  | Nasopharynx |
| srn277-SP1142-SP1143 | 0.00 |  |  | 20 | 20 | 14 | Nasopharynx |
| srn279-SP1166-SP1167 | 1.02 | 0.05 | 0.03 | 5 | 3 | 3 | Nasopharynx |
| srn299-SP1278-SP1279 | n.i. |  |  |  |  |  | Nasopharynx |
| srn308-SP1355-SP1356 | n.i. |  |  |  |  |  | Nasopharynx |
| srn317-SP1415-SP1416 | 0.90 | 0.05 | 0.03 | 11 | 8 | 8 | Nasopharynx |
| srn319-SP1440-SP1441 | n.i. |  |  |  |  |  | Nasopharynx |
| srn351-SP1551-SP1552 | n.i. |  |  |  |  |  | Nasopharynx |
| srn395-SP1790-SP1791 | n.i. |  |  |  |  |  | Nasopharynx |
| srn400-SP1820-SP1821 | n.i. |  |  |  |  |  | Nasopharynx |
| srn435-SP1963-SP1964 | n.i. |  |  |  |  |  | Nasopharynx |
| srn448-SP1998-SP1999 | n.i. |  |  |  |  |  | Nasopharynx |
| srn477-SP2075-SP2076 | n.i. |  |  |  |  |  | Nasopharynx |
| srn478-SP2075-SP2077 | n.i. |  |  |  |  |  | Nasopharynx |
| srn491-SP2097-SP2098 | n.i. |  |  |  |  |  | Nasopharynx |
| srn502-SP2168-SP2169 | 0.00 |  |  | 8 | 8 | 5 | Nasopharynx |
| srn503-SP2168-SP2169 | n.i. |  |  |  |  |  | Nasopharynx |
| srn142-SP0486-SP0487 | 0.00 |  |  | 5 | 5 | 3 | Nasopharynx |
| srn368-SP1629-SP1630 | n.i. |  |  |  |  |  | Nasopharynx |
| trn0012-SP0019-SP0020 | 1.18 | 0.04 | 0.03 | 5 | 3 | 3 | Nasopharynx |
| trn0027-SP0071-SP0072 | 0.54 | 0.54 | 0.38 | 4 | 3 | 2 | Nasopharynx |
| trn0036-SP0086-SP0087 | 0.92 | 0.32 | 0.18 | 9 | 7 | 6 | Nasopharynx |
| trn0052-SP0117-SP0118 | 0.00 |  |  | 2 | 2 | 1 | Nasopharynx |
| trn0057-SP0120-SP0121 | n.i. |  |  |  |  |  | Nasopharynx |
| trn0093-SP0223-SP0224 | n.i. |  |  |  |  |  | Nasopharynx |
| trn0156-SP0311-SP0312 | 0.00 |  |  | 5 | 5 | 3 | Nasopharynx |
| trn0157-SP0311-SP0312 | 1.05 | 0.04 | 0.02 | 12 | 8 | 8 | Nasopharynx |
| trn0218-SP0411-SP0412 | n.i. |  |  |  |  |  | Nasopharynx |
| trn0256-SP0460-SP0461 | 1.02 | 0.09 | 0.05 | 6 | 3 | 3 | Nasopharynx |
| trn0273-SP0484-SP0486 | n.i. |  |  |  |  |  | Nasopharynx |
| trn0290-SP0516-SP0517 | n.i. |  |  |  |  |  | Nasopharynx |
| trn0329-SP0640-SP0641 | 0.99 | 0.07 | 0.05 | 6 | 4 | 4 | Nasopharynx |
| trn0332-SP0648-SP0650 | 0.97 | 0.04 | 0.03 | 6 | 4 | 4 | Nasopharynx |
| trn0335-SP0707-SP0709 | n.i. |  |  |  |  |  | Nasopharynx |
| trn0358-SP0718-SP0719 | n.i. |  |  |  |  |  | Nasopharynx |
| trn0423-SP0854-SP0855 | n.i. |  |  |  |  |  | Nasopharynx |
| trn0485-SP1004-SP1006 | 0.00 |  |  | 2 | 2 | 1 | Nasopharynx |
| trn0489-dapA-trmE | n.i. |  |  |  |  |  | Nasopharynx |
| trn0498-SP1045-SP1047 | n.i. |  |  |  |  |  | Nasopharynx |
| trn0500-SP1065-SP1067 | n.i. |  |  |  |  |  | Nasopharynx |
| trn0510-SP1080-SP1081 | n.i. |  |  |  |  |  | Nasopharynx |
| trn0573-SP1220-SP1221 | n.i. |  |  |  |  |  | Nasopharynx |
| trn0591-SP1256-SP1258 | n.i. |  |  |  |  |  | Nasopharynx |
| trn0634-SP1384-SP1385 | n.i. |  |  |  |  |  | Nasopharynx |
| trn0663-SP1477-SP1478 | n.i. |  |  |  |  |  | Nasopharynx |
| trn0674-SP1490-SP1491 | n.i. |  |  |  |  |  | Nasopharynx |
| trn0696-SP1514-SP1516 | n.i. |  |  |  |  |  | Nasopharynx |
| trn0723-SP1552-SP1553 | n.i. |  |  |  |  |  | Nasopharynx |
| trn0757-SP1612-SP1613 | n.i. |  |  |  |  |  | Nasopharynx |
| trn0760-SP1625-SP1626 | 0.00 |  |  | 8 | 8 | 5 | Nasopharynx |
| trn0761-SP1625-SP1626 | 0.90 | 0.00 | 0.00 | 3 | 2 | 2 | Nasopharynx |
| trn0788-SP1692-SP1694 | n.i. |  |  |  |  |  | Nasopharynx |
| trn0830-SP1818-SP1819 | 0.00 |  |  | 5 | 5 | 3 | Nasopharynx |
| trn0866-SP1900-tRNASer | n.i. |  |  |  |  |  | Nasopharynx |
| trn0883-SP1922-SP1923 | n.i. |  |  |  |  |  | Nasopharynx |
| trn0884-SP1922-SP1923 | n.i. |  |  |  |  |  | Nasopharynx |
| trn0909-rpoB-tRNAcys | n.i. |  |  |  |  |  | Nasopharynx |
| trn0935-SP1999-SP2000 | n.i. |  |  |  |  |  | Nasopharynx |
| trn0978-SP2075-SP2077 | n.i. |  |  |  |  |  | Nasopharynx |
| trn1009-SP2103-SP2104 | 0.00 |  |  | 3 | 3 | 2 | Nasopharynx |
| trn1025-SP2136-SP2138 | 0.00 |  |  | 5 | 5 | 3 | Nasopharynx |
| trn1031-SP2153-SP2156 | n.i. |  |  |  |  |  | Nasopharynx |
| trn1052-SP2210-tsf | n.i. |  |  |  |  |  | Nasopharynx |
| SN1-SP0019-SP0020 | 1.11 | 0.11 | 0.06 | 7 | 4 | 4 | Nasopharynx |
| SN2-SP0041-SP0042 | 1.03 | 0.10 | 0.05 | 18 | 13 | 13 | Nasopharynx |
| SN3-SP0114-SP0115 | 1.06 | 0.12 | 0.03 | 43 | 29 | 29 | Nasopharynx |
| SN4-SP0178-SP0179 | 0.94 | 0.05 | 0.02 | 18 | 13 | 13 | Nasopharynx |
| SN5-SP0256-SP0257 | 0.99 | 0.11 | 0.06 | 4 | 0 | 0 | Nasopharynx |
| SN6-SP0257-SP0258 | 1.04 | 0.11 | 0.05 | 9 | 5 | 5 | Nasopharynx |
| SN7-SP0260-SP0261 | 0.00 |  |  | 3 | 3 | 2 | Nasopharynx |
| SN8-SP0372-SP0373 | 0.00 |  |  | 3 | 3 | 2 | Nasopharynx |
| SN9-SP0439-SP0440 | n.i. |  |  |  |  |  | Nasopharynx |
| SN10-SP0486-SP0487 | 0.00 |  |  | 5 | 5 | 3 | Nasopharynx |
| SN11-SP0649-SP0650 | 0.97 | 0.04 | 0.03 | 6 | 4 | 4 | Nasopharynx |
| SN12-SP0700-SP0701 | 0.00 |  |  | 3 | 3 | 2 | Nasopharynx |
| SN13-SP0715-SP0716 | n.i. |  |  |  |  |  | Nasopharynx |
| SN14-SP0834-SP0835 | n.i. |  |  |  |  |  | Nasopharynx |
| SN15-SP0873-SP0874 | n.i. |  |  |  |  |  | Nasopharynx |
| SN16-SP0873-SP0874 | 0.00 |  |  | 2 | 2 | 1 | Nasopharynx |
| SN17-SP0897-SP0898 | 1.03 | 0.07 | 0.02 | 35 | 12 | 12 | Nasopharynx |
| SN18-SP0898-SP0899 | 0.00 |  |  | 3 | 3 | 2 | Nasopharynx |
| SN19-SP0899-SP0900 | 0.00 |  |  | 6 | 6 | 4 | Nasopharynx |
| SN20-SP0915-SP0916 | 0.00 |  |  | 2 | 2 | 1 | Nasopharynx |
| SN21-SP1068-SP1069 | n.i. |  |  |  |  |  | Nasopharynx |
| SN22-SP1100-SP1101 | n.i. |  |  |  |  |  | Nasopharynx |
| SN23-SP1400-SP1401 | 0.29 | 0.42 | 0.24 | 9 | 8 | 6 | Nasopharynx |
| SN24-SP1629-SP1630 | n.i. |  |  |  |  |  | Nasopharynx |
| SN25-SP1691-SP1692 | 1.03 | 0.06 | 0.02 | 36 | 23 | 23 | Nasopharynx |
| SN26-SP2078-SP2079 | n.i. |  |  |  |  |  | Nasopharynx |
| SN27-SP2168-SP2169 | 0.34 | 0.46 | 0.21 | 16 | 14 | 11 | Nasopharynx |
| SN28-SP0502-SP0503 | n.i. |  |  |  |  |  | Nasopharynx |
| SN29-SP0516-SP0517 | 0.00 |  |  | 2 | 2 | 1 | Nasopharynx |
| SN30-SP0518-SP0519 | 0.00 |  |  | 11 | 11 | 8 | Nasopharynx |
| SN31-SP2092-SP2093 | 1.16 | 0.12 | 0.09 | 4 | 2 | 2 | Nasopharynx |
| SN32-SP1086-SP1087 | 0.00 |  |  | 3 | 3 | 2 | Nasopharynx |
| SN33-SP0411-SP0412 | n.i. |  |  |  |  |  | Nasopharynx |
| SN34-SP1790-SP1791 | n.i. |  |  |  |  |  | Nasopharynx |
| SN35-SP0239-SP0240 | 1.02 | 0.04 | 0.02 | 20 | 13 | 13 | Nasopharynx |
| SN36-SP0451-SP0452 | 0.34 | 0.48 | 0.28 | 9 | 8 | 6 | Nasopharynx |
| SN37-SP0587-SP0588 | n.i. |  |  |  |  |  | Nasopharynx |
| SN38-SP0502-SP0503 | 0.00 |  |  | 6 | 6 | 4 | Nasopharynx |
| SN39-SP0761-SP0762 | 0.00 |  |  | 8 | 8 | 5 | Nasopharynx |
| SN40-SP0958-SP0959 | n.i. |  |  |  |  |  | Nasopharynx |
| SN41-SP1104-SP1105 | 0.00 |  |  | 3 | 3 | 2 | Nasopharynx |
| SN42-SP1278-SP1279 | n.i. |  |  |  |  |  | Nasopharynx |
| SN43-SP1355-SP1356 | 0.95 | 0.08 | 0.06 | 3 | 1 | 1 | Nasopharynx |
| SN44-SP1551-SP1552 | n.i. |  |  |  |  |  | Nasopharynx |
| SN45-SP2097-SP2098 | 0.00 |  |  | 2 | 2 | 1 | Nasopharynx |
| SN46-SP2136-SP2137 | 0.00 |  |  | 8 | 8 | 5 | Nasopharynx |
| SN47-SP0051-SP0052 | n.i. |  |  |  |  |  | Nasopharynx |
| SN48-SP1166-SP1167 | n.i. |  |  |  |  |  | Nasopharynx |
| SN49-SP1547-SP1548 | 0.54 | 0.54 | 0.38 | 5 | 4 | 3 | Nasopharynx |
| SN50-SP1966-SP1967 | 1.13 | 0.05 | 0.02 | 13 | 3 | 3 | Nasopharynx |
| F01-SP0085-SP0086 | 1.037 | 0.449 | 0.224 | 6 | 3 | 2 | Blood |
| F02-SP0103-SP0104 | 0.533 | 0.614 | 0.307 | 5 | 4 | 1 | Blood |
| F03-SP0115-SP0116 | n.d. |  |  | n.i. |  |  | Blood |
| F04-SP0116-SP0117 | n.d. |  |  | n.i. |  |  | Blood |
| F05-SP0117-SP0118 | 0 |  |  | 9 | 9 | 3 | Blood |
| F06-SP0129-SP0130 | n.d. |  |  | n.i. |  |  | Blood |
| F07-SP0239-SP0240 | n.d. |  |  | n.i. |  |  | Blood |
| F08-SP0256-SP0257 | n.d. |  |  | n.i. |  |  | Blood |
| F09-SP0257-SP0258 | n.d. |  |  | n.i. |  |  | Blood |
| F10-SP0311-SP0312 | n.d. |  |  | n.i. |  |  | Blood |
| F11-SP0312-SP0313 | 0.986 | 0.098 | 0.049 | 4 | 0 | 0 | Blood |
| F12-SP0464-SP0465 | n.d. |  |  | n.i. |  |  | Blood |
| F13-SP0493-SP0494 | 0 |  |  | 2 | 2 | 0 | Blood |
| F14-SP0518-SP0519 | 0.902 | 0.264 | 0.052 | 41 | 17 | 15 | Blood |
| F15-SP0519-SP0520 | 0.943 | 0.106 | 0.020 | 32 | 4 | 4 | Blood |
| F16-SP0560-SP0561 | 1.025 | 0.259 | 0.078 | 11 | 0 | 0 | Blood |
| F17-SP0564-SP0565 | 0 |  |  | 1 | 1 | 0 | Blood |
| F18-SP0571-SP0572 | n.d. |  |  | n.i. |  |  | Blood |
| F19-SP0575-SP0576 | n.d. |  |  | n.i. |  |  | Blood |
| F20-SP0578-SP0579 | 0.969 | 0.489 | 0.109 | 31 | 15 | 11 | Blood |
| F21-SP0586-SP0587 | n.d. |  |  | n.i. |  |  | Blood |
| F22-SP0625-SP0626 | n.d. |  |  | n.i. |  |  | Blood |
| F23-SP0640-SP0641 | 1.005 | 0.116 | 0.033 | 13 | 1 | 1 | Blood |
| F24-SP0641-SP0642 | 1.113 |  |  | 1 | 0 | 0 | Blood |
| F25-SP0649-SP0650 | 0.824 | 0.069 | 0.034 | 4 | 0 | 0 | Blood |
| F26-SP0700-SP0701 | n.d. |  |  | n.i. |  |  | Blood |
| F27-SP0718-SP0719 | 0.895 | 0.046 | 0.017 | 7 | 0 | 0 | Blood |
| F28-SP0749-SP0750 | n.d. |  |  | n.i. |  |  | Blood |
| F29-SP0788-SP0789 | n.d. |  |  | n.i. |  |  | Blood |
| F30-SP0788-SP0789 | n.d. |  |  | n.i. |  |  | Blood |
| F31-SP0863-SP0864 | n.d. |  |  | n.i. |  |  | Blood |
| F32-SP0873-SP0874 | n.d. |  |  | n.i. |  |  | Blood |
| F33-SP0910-SP0911 | n.d. |  |  | n.i. |  |  | Blood |
| F34-SP0958-SP0959 | n.d. |  |  | n.i. |  |  | Blood |
| F35-SP0962-SP0963 | n.d. |  |  | n.i. |  |  | Blood |
| F36-SP1000-SP1001 | n.d. |  |  | n.i. |  |  | Blood |
| F38-SP1012-SP1013 | 1.060 | 0.204 | 0.072 | 12 | 4 | 4 | Blood |
| F39-SP1029-SP1030 | n.d. |  |  | n.i. |  |  | Blood |
| F40-SP1128-SP1129 | n.d. |  |  | n.i. |  |  | Blood |
| F41-SP1142-SP1143 | 0.334 | 0.510 | 0.170 | 13 | 11 | 4 | Blood |
| F42-SP1157-SP1158 | 0.912 | 0.332 | 0.136 | 9 | 4 | 3 | Blood |
| F43-SP1281-SP1282 | n.d. |  |  | n.i. |  |  | Blood |
| F44-SP1285-SP1286 | n.d. |  |  | n.i. |  |  | Blood |
| F45-SP1499-SP1500 | 0.836 | 0.099 | 0.038 | 7 | 0 | 0 | Blood |
| F46-SP1546-SP1547 | 1.004 | 0.076 | 0.029 | 7 | 0 | 0 | Blood |
| F47-SP1629-SP1630 | n.d. |  |  | n.i. |  |  | Blood |
| F48-SP1872-SP1873 | n.d. |  |  | n.i. |  |  | Blood |
| F49-SP2112-SP2113 | n.d. |  |  | n.i. |  |  | Blood |
| F50-SP2168-SP2169 | n.d. |  |  | n.i. |  |  | Blood |
| F51-SP2213-SP2214 | 1.001 | 0.094 | 0.038 | 6 | 0 | 0 | Blood |
| F52-SP0041-SP0042 | 1.037 | 0.042 | 0.017 | 6 | 0 | 0 | Blood |
| F53-SP0619-SP0620 | n.d. |  |  | n.i. |  |  | Blood |
| F54-SP1516-SP1517 | n.d. |  |  | n.i. |  |  | Blood |
| F55-SP1777-SP1778 | n.d. |  |  | n.i. |  |  | Blood |
| F56-SP0162-SP0163 | n.d. |  |  | n.i. |  |  | Blood |
| F57-SP0584-SP0585 | n.d. |  |  | n.i. |  |  | Blood |
| F58-SP0757-SP0758 | n.d. |  |  | n.i. |  |  | Blood |
| F59-SP0915-SP0916 | n.d. |  |  | n.i. |  |  | Blood |
| F60-SP1004-SP1005 | 0 |  |  | 1 | 1 | 0 | Blood |
| F61-SP1029-SP1030 | 0.895 | 0.248 | 0.088 | 9 | 1 | 1 | Blood |
| F62-SP1059-SP1060 | n.d. |  |  | n.i. |  |  | Blood |
| F63-SP1154-SP1155 | 1.092 | 0.035 | 0.013 | 8 | 0 | 0 | Blood |
| F64-SP1179-SP1180 | 1.219 | 0.047 | 0.033 | 2 | 0 | 0 | Blood |
| F65-SP1431-SP1432 | n.d. |  |  | n.i. |  |  | Blood |
| F66-SP2168-SP2169 | n.d. |  |  | n.i. |  |  | Blood |
| F67-SP2168-SP2169 | n.d. |  |  | n.i. |  |  | Blood |
| F68-SP2213-SP2214 | n.d. |  |  | n.i. |  |  | Blood |
| R01-SP0178-SP0179 | n.d. |  |  | n.i. |  |  | Blood |
| R02-SP1286-SP1287 | n.d. |  |  | n.i. |  |  | Blood |
| R03-SP1355-SP1356 | n.d. |  |  | n.i. |  |  | Blood |
| R04-SP1431-SP1432 | 0.273 | 0.416 | 0.186 | 7 | 6 | 2 | Blood |
| R06-SP1820-SP1821 | 0.954 | 0.060 | 0.023 | 7 | 0 | 0 | Blood |
| R07-SP1886-SP1887 | n.d. |  |  | n.i. |  |  | Blood |
| R08-SP1988-SP1989 | 0.747 | 0.137 | 0.069 | 5 | 1 | 1 | Blood |
| R09-SP1999-SP2000 | n.d. |  |  | n.i. |  |  | Blood |
| R10-SP2076-SP2077 | n.d. |  |  | n.i. |  |  | Blood |
| R11-SP2093-SP2094 | n.d. |  |  | n.i. |  |  | Blood |
| R12-SP1818-SP1819 | 0.742 | 0.121 | 0.054 | 6 | 1 | 1 | Blood |
| R13-SP0649-SP0650 | n.d. |  |  | n.i. |  |  | Blood |
| R14-SP1100-SP1101 | n.d. |  |  | n.i. |  |  | Blood |
| R15-SP1278-SP1279 | n.d. |  |  | n.i. |  |  | Blood |
| R16-SP1292-SP1293 | 0.995 | 0.180 | 0.050 | 20 | 7 | 7 | Blood |
| R17-SP1356-SP1357 | n.d. |  |  | n.i. |  |  | Blood |
| R18-SP1444-SP1445 | n.d. |  |  | n.i. |  |  | Blood |
| R19-SP1477-SP1478 | n.d. |  |  | n.i. |  |  | Blood |
| R20-SP1547-SP1548 | n.d. |  |  | n.i. |  |  | Blood |
| R21-SP1551-SP1552 | n.d. |  |  | n.i. |  |  | Blood |
| srn026-SP0145-SP0146 | n.i. |  |  |  |  |  | Blood |
| srn029-SP0178-SP0179 | 0.87 | 0.13 | 0.04 | 10 | 0 | 0 | Blood |
| srn049-rpmD-rplO | n.i. |  |  |  |  |  | Blood |
| srn061-SP0239-SP0240 | n.i. |  |  |  |  |  | Blood |
| srn068-SP0272-SP0273 | 0.94 | 0.11 | 0.05 | 4 | 0 | 0 | Blood |
| srn081-SP0297-SP0298 | 0.54 | 0.55 | 0.22 | 9 | 7 | 3 | Blood |
| srn098-SP0372-SP0373 | n.i. |  |  |  |  |  | Blood |
| srn135-SP0461-SP0462 | 0.92 | 0.13 | 0.05 | 9 | 1 | 1 | Blood |
| srn141-SP0482-SP0483 | n.i. |  |  |  |  |  | Blood |
| srn145-SP0493-SP0494 | 0.00 |  |  | 1 | 1 | 0 | Blood |
| srn151-SP0519-SP0520 | 0.80 | 0.43 | 0.19 | 7 | 3 | 2 | Blood |
| srn157-SP0578-SP0579 | 0.80 | 0.33 | 0.13 | 6 | 0 | 0 | Blood |
| srn164-SP0629-SP0630 | 0.86 | 0.51 | 0.25 | 6 | 4 | 2 | Blood |
| srn176-SP0715-SP0716 | n.i. |  |  |  |  |  | Blood |
| srn206-SP0831-SP0832 | 1.04 | 0.29 | 0.08 | 20 | 7 | 7 | Blood |
| srn218-tRNAGln-rpsA | n.i. |  |  |  |  |  | Blood |
| srn226-SP0873-SP0874 | n.i. |  |  |  |  |  | Blood |
| srn231-SP0897-SP0899 | 0.99 | 0.16 | 0.07 | 5 | 0 | 0 | Blood |
| srn235-SP0915-SP0916 | n.i. |  |  |  |  |  | Blood |
| srn239-SP0958-SP0959 | n.i. |  |  |  |  |  | Blood |
| srn241-SP0962-SP0963 | n.i. |  |  |  |  |  | Blood |
| srn249-SP0983-SP0984 | n.i. |  |  |  |  |  | Blood |
| srn254-SP1012-SP1013 | 1.09 | 0.13 | 0.07 | 5 | 1 | 1 | Blood |
| srn266-SP1101-SP1102 | 0.87 | 0.10 | 0.04 | 7 | 1 | 1 | Blood |
| srn267-SP1104-rplU | n.i. |  |  |  |  |  | Blood |
| srn270-SP1107-SP1108 | n.i. |  |  |  |  |  | Blood |
| srn277-SP1142-SP1143 | 0.59 | 0.55 | 0.17 | 16 | 12 | 6 | Blood |
| srn279-SP1166-SP1167 | 1.34 | 0.05 | 0.02 | 7 | 2 | 2 | Blood |
| srn299-SP1278-SP1279 | n.i. |  |  |  |  |  | Blood |
| srn308-SP1355-SP1356 | n.i. |  |  |  |  |  | Blood |
| srn317-SP1415-SP1416 | 0.91 | 0.31 | 0.08 | 21 | 9 | 7 | Blood |
| srn319-SP1440-SP1441 | n.i. |  |  |  |  |  | Blood |
| srn351-SP1551-SP1552 | n.i. |  |  |  |  |  | Blood |
| srn395-SP1790-SP1791 | n.i. |  |  |  |  |  | Blood |
| srn400-SP1820-SP1821 | n.i. |  |  |  |  |  | Blood |
| srn435-SP1963-SP1964 | n.i. |  |  |  |  |  | Blood |
| srn448-SP1998-SP1999 | n.i. |  |  |  |  |  | Blood |
| srn477-SP2075-SP2076 | n.i. |  |  |  |  |  | Blood |
| srn478-SP2075-SP2077 | n.i. |  |  |  |  |  | Blood |
| srn491-SP2097-SP2098 | n.i. |  |  |  |  |  | Blood |
| srn502-SP2168-SP2169 | n.i. |  |  |  |  |  | Blood |
| srn503-SP2168-SP2169 | n.i. |  |  |  |  |  | Blood |
| srn142-SP0486-SP0487 | 1.05 | 0.09 | 0.04 | 8 | 2 | 2 | Blood |
| srn368-SP1629-SP1630 | n.i. |  |  |  |  |  | Blood |
| trn0012-SP0019-SP0020 | 0.00 |  |  | 9 | 9 | 3 | Blood |
| trn0027-SP0071-SP0072 | 0.59 | 0.65 | 0.23 | 12 | 8 | 4 | Blood |
| trn0036-SP0086-SP0087 | 1.04 | 0.45 | 0.22 | 6 | 3 | 2 | Blood |
| trn0052-SP0117-SP0118 | 0.00 |  |  | 9 | 9 | 3 | Blood |
| trn0057-SP0120-SP0121 | n.i. |  |  |  |  |  | Blood |
| trn0093-SP0223-SP0224 | n.i. |  |  |  |  |  | Blood |
| trn0156-SP0311-SP0312 | 0.85 | 0.28 | 0.11 | 6 | 0 | 0 | Blood |
| trn0157-SP0311-SP0312 | 0.84 | 0.21 | 0.07 | 8 | 0 | 0 | Blood |
| trn0218-SP0411-SP0412 | n.i. |  |  |  |  |  | Blood |
| trn0256-SP0460-SP0461 | 0.54 | 0.54 | 0.31 | 4 | 2 | 1 | Blood |
| trn0273-SP0484-SP0486 | n.i. |  |  |  |  |  | Blood |
| trn0290-SP0516-SP0517 | n.i. |  |  |  |  |  | Blood |
| trn0329-SP0640-SP0641 | 0.64 | 0.45 | 0.19 | 9 | 5 | 3 | Blood |
| trn0332-SP0648-SP0650 | 0.82 | 0.07 | 0.03 | 4 | 0 | 0 | Blood |
| trn0335-SP0707-SP0709 | n.i. |  |  |  |  |  | Blood |
| trn0358-SP0718-SP0719 | n.i. |  |  |  |  |  | Blood |
| trn0423-SP0854-SP0855 | n.i. |  |  |  |  |  | Blood |
| trn0485-SP1004-SP1006 | 0.00 |  |  | 1 | 1 | 0 | Blood |
| trn0489-dapA-trmE | n.i. |  |  |  |  |  | Blood |
| trn0498-SP1045-SP1047 | n.i. |  |  |  |  |  | Blood |
| trn0500-SP1065-SP1067 | n.i. |  |  |  |  |  | Blood |
| trn0510-SP1080-SP1081 | n.i. |  |  |  |  |  | Blood |
| trn0573-SP1220-SP1221 | n.i. |  |  |  |  |  | Blood |
| trn0591-SP1256-SP1258 | n.i. |  |  |  |  |  | Blood |
| trn0634-SP1384-SP1385 | n.i. |  |  |  |  |  | Blood |
| trn0663-SP1477-SP1478 | n.i. |  |  |  |  |  | Blood |
| trn0674-SP1490-SP1491 | n.i. |  |  |  |  |  | Blood |
| trn0696-SP1514-SP1516 | n.i. |  |  |  |  |  | Blood |
| trn0723-SP1552-SP1553 | n.i. |  |  |  |  |  | Blood |
| trn0757-SP1612-SP1613 | n.i. |  |  |  |  |  | Blood |
| trn0760-SP1625-SP1626 | 0.97 | 0.13 | 0.04 | 13 | 1 | 1 | Blood |
| trn0761-SP1625-SP1626 | 0.44 | 0.53 | 0.22 | 9 | 8 | 3 | Blood |
| trn0788-SP1692-SP1694 | n.i. |  |  |  |  |  | Blood |
| trn0830-SP1818-SP1819 | 0.74 | 0.12 | 0.05 | 6 | 1 | 1 | Blood |
| trn0866-SP1900-tRNASer | n.i. |  |  |  |  |  | Blood |
| trn0883-SP1922-SP1923 | n.i. |  |  |  |  |  | Blood |
| trn0884-SP1922-SP1923 | n.i. |  |  |  |  |  | Blood |
| trn0909-rpoB-tRNAcys | n.i. |  |  |  |  |  | Blood |
| trn0935-SP1999-SP2000 | n.i. |  |  |  |  |  | Blood |
| trn0978-SP2075-SP2077 | n.i. |  |  |  |  |  | Blood |
| trn1009-SP2103-SP2104 | 0.97 | 0.53 | 0.26 | 5 | 2 | 1 | Blood |
| trn1025-SP2136-SP2138 | 1.22 | 0.03 | 0.02 | 5 | 1 | 1 | Blood |
| trn1031-SP2153-SP2156 | n.i. |  |  |  |  |  | Blood |
| trn1052-SP2210-tsf | n.i. |  |  |  |  |  | Blood |
| SN1-SP0019-SP0020 | 0.74 | 0.48 | 0.15 | 16 | 11 | 6 | Blood |
| SN2-SP0041-SP0042 | 0.89 | 0.31 | 0.08 | 19 | 5 | 5 | Blood |
| SN3-SP0114-SP0115 | 0.92 | 0.31 | 0.05 | 52 | 21 | 19 | Blood |
| SN4-SP0178-SP0179 | 1.00 | 0.13 | 0.03 | 25 | 4 | 4 | Blood |
| SN5-SP0256-SP0257 | 0.52 | 0.50 | 0.29 | 4 | 3 | 1 | Blood |
| SN6-SP0257-SP0258 | 1.26 | 0.09 | 0.06 | 3 | 1 | 1 | Blood |
| SN7-SP0260-SP0261 | 0.54 | 0.55 | 0.23 | 9 | 7 | 3 | Blood |
| SN8-SP0372-SP0373 | 1.39 | 0.41 | 0.24 | 3 | 0 | 0 | Blood |
| SN9-SP0439-SP0440 | n.i. |  |  |  |  |  | Blood |
| SN10-SP0486-SP0487 | 1.05 | 0.09 | 0.04 | 8 | 2 | 2 | Blood |
| SN11-SP0649-SP0650 | 0.82 | 0.07 | 0.03 | 4 | 0 | 0 | Blood |
| SN12-SP0700-SP0701 | n.i. |  |  |  |  |  | Blood |
| SN13-SP0715-SP0716 | n.i. |  |  |  |  |  | Blood |
| SN14-SP0834-SP0835 | n.i. |  |  |  |  |  | Blood |
| SN15-SP0873-SP0874 | n.i. |  |  |  |  |  | Blood |
| SN16-SP0873-SP0874 | n.i. |  |  |  |  |  | Blood |
| SN17-SP0897-SP0898 | 1.02 | 0.27 | 0.08 | 17 | 5 | 5 | Blood |
| SN18-SP0898-SP0899 | 0.36 | 0.53 | 0.26 | 5 | 4 | 1 | Blood |
| SN19-SP0899-SP0900 | 0.92 | 0.13 | 0.05 | 6 | 0 | 0 | Blood |
| SN20-SP0915-SP0916 | n.i. |  |  |  |  |  | Blood |
| SN21-SP1068-SP1069 | n.i. |  |  |  |  |  | Blood |
| SN22-SP1100-SP1101 | n.i. |  |  |  |  |  | Blood |
| SN23-SP1400-SP1401 | 1.02 | 0.12 | 0.05 | 8 | 2 | 2 | Blood |
| SN24-SP1629-SP1630 | n.i. |  |  |  |  |  | Blood |
| SN25-SP1691-SP1692 | 0.95 | 0.37 | 0.07 | 43 | 21 | 16 | Blood |
| SN26-SP2078-SP2079 | n.i. |  |  |  |  |  | Blood |
| SN27-SP2168-SP2169 | 0.86 | 0.14 | 0.04 | 11 | 0 | 0 | Blood |
| SN28-SP0502-SP0503 | n.i. |  |  |  |  |  | Blood |
| SN29-SP0516-SP0517 | n.i. |  |  |  |  |  | Blood |
| SN30-SP0518-SP0519 | 0.93 | 0.05 | 0.02 | 6 | 0 | 0 | Blood |
| SN31-SP2092-SP2093 | 0.77 | 0.51 | 0.19 | 11 | 7 | 4 | Blood |
| SN32-SP1086-SP1087 | n.i. |  |  |  |  |  | Blood |
| SN33-SP0411-SP0412 | n.i. |  |  |  |  |  | Blood |
| SN34-SP1790-SP1791 | n.i. |  |  |  |  |  | Blood |
| SN35-SP0239-SP0240 | 1.06 | 0.14 | 0.04 | 18 | 3 | 3 | Blood |
| SN36-SP0451-SP0452 | 0.99 | 0.03 | 0.01 | 6 | 0 | 0 | Blood |
| SN37-SP0587-SP0588 | n.i. |  |  |  |  |  | Blood |
| SN38-SP0502-SP0503 | 0.00 |  |  | 5 | 5 | 1 | Blood |
| SN39-SP0761-SP0762 | 1.01 | 0.03 | 0.01 | 6 | 1 | 1 | Blood |
| SN40-SP0958-SP0959 | n.i. |  |  |  |  |  | Blood |
| SN41-SP1104-SP1105 | n.i. |  |  |  |  |  | Blood |
| SN42-SP1278-SP1279 | n.i. |  |  |  |  |  | Blood |
| SN43-SP1355-SP1356 | 1.02 | 0.04 | 0.02 | 6 | 0 | 0 | Blood |
| SN44-SP1551-SP1552 | n.i. |  |  |  |  |  | Blood |
| SN45-SP2097-SP2098 | n.i. |  |  |  |  |  | Blood |
| SN46-SP2136-SP2137 | 1.19 | 0.09 | 0.03 | 12 | 2 | 2 | Blood |
| SN47-SP0051-SP0052 | n.i. |  |  |  |  |  | Blood |
| SN48-SP1166-SP1167 | n.i. |  |  |  |  |  | Blood |
| SN49-SP1547-SP1548 | 0.55 | 0.56 | 0.28 | 5 | 4 | 1 | Blood |
| SN50-SP1966-SP1967 | 1.10 | 0.15 | 0.04 | 20 | 6 | 6 | Blood |

**Table S5. Microarray analysis of attenuated sRNA mutants.** Log (2) changes compared to TIGR4. All samples were compared to the parental TIGR4 grown under identical conditions. All differences were considered significant with a *p<0.05* by t-distribution test.

| **Gene** | **Description** | **F7-** | **F20-** | **F22-** | **F25-** | **F32-** | **F41-** | **F44-** | **F48-** |
| --- | --- | --- | --- | --- | --- | --- | --- | --- | --- |
| SP0018 | hypothetical protein |  |  | **-1.4563** | **-2.095** | **-2.141** |  |  |  |
| SP0021 | deoxyuridine 5triphosphate nucleotidohydrolase, putative |  |  |  | **-1.435** | **-1.249** |  |  |  |
| SP0022 | conserved hypothetical protein |  |  |  | **-1.304** | **-1.184** |  |  |  |
| SP0023 | DNA repair protein RadA, authentic point mutation |  |  |  | **-1.363** | **-1.116** |  |  |  |
| SP0024 | conserved hypothetical protein |  |  |  | **-1.498** |  |  |  |  |
| SP0025 | hypothetical protein |  |  |  | **-1.583** |  |  |  |  |
| SP0026 | hypothetical protein |  |  |  | **-1.84** |  |  |  |  |
| SP0033 | conserved hypothetical protein |  |  |  |  |  |  |  | **1.545** |
| SP0042 | competence factor transporting ATP-binding permease protein ComA |  |  |  |  | **-1.236** |  |  |  |
| SP0043 | competence factor transport protein ComB |  | **1.8135** | **-1.259** |  | **-1.21** |  |  |  |
| SP0044 | phosphoribosylaminoimidazole-succinocarboxamide synthase |  |  | **4.265** | **3.378** | **2.73** |  |  |  |
| SP0046 | amidophosphoribosyltransferase |  |  | **4.3305** | **3.821** |  |  |  | **-2.2455** |
| SP0049 | vanZ protein, putative |  |  | **4.392** | **3.87** |  |  |  | **-2.3955** |
| SP0050 | phosphoribosylaminoimidazolecarboxamide formyltransferase-IMP cyclohydrolase | **1.066** |  | **3.7905** | **4.138** |  |  |  | **-1.7375** |
| SP0051 | PHOSPHORIBOSYLAMINE--GLYCINE LIGASE (EC 6.3.4.13) (GARS) ] |  |  |  |  | **1.939** |  |  |  |
| SP0052 | hypothetical protein | **1.3087** |  | **2.6903** | **3.046** | **1.917** |  |  | **-2.3395** |
| SP0053 | phosphoribosylaminoimidazole carboxylase, catalytic subunit | **1.482** |  | **2.551** | **2.731** | **1.784** |  |  | **-2.266** |
| SP0054 | phosphoribosylaminoimidazole carboxylase, ATPase subunit |  |  |  | **1.691** |  |  |  |  |
| SP0055 | hypothetical protein |  |  |  | **1.638** |  |  |  |  |
| SP0056 | adenylosuccinate lyase |  |  |  | **1.614** |  |  |  |  |
| SP0058 | transcriptional regulator, GntR family |  |  | **1.0313** |  |  |  |  |  |
| SP0060 | beta-galactosidase |  | **2.1535** |  | **1.259** | **-3.299** |  | **3.013** | **3.407** |
| SP0061 | PTS system, IIB component |  |  |  | **1.339** | **-2.819** | **1.326** | **2.973** | **2.418** |
| SP0062 | Phosphotransferase system sugar-specific EIIC component |  |  |  |  | **-1.914** |  |  |  |
| SP0063 | Phosphotransferase system sugar-specific EIID component |  |  |  |  | **-1.963** |  |  |  |
| SP0064 | PTS system, IIA component |  |  |  |  | **-1.79** | **1.016** | **2.418** | **1.6945** |
| SP0065 | sugar isomerase domain protein AgaS |  |  |  | **1.084** | **-1.619** | **1.038** | **2.469** |  |
| SP0066 | Aldose-1-epimerase (mutarotase) |  |  |  |  | **-1.396** |  |  |  |
| SP0067 | hypothetical protein |  |  |  |  | **-1.268** |  | **2.298** |  |
| SP0068 | HYPOTHETICAL PROTEIN HOMOLOGOUS TO SPT01510 |  |  |  |  | **-1.185** |  |  |  |
| SP0069 | choline binding protein I |  |  |  |  | **-1.216** |  | **2.167** | **2.207** |
| SP0070 | hypothetical protein |  |  |  |  | **-1.113** |  |  |  |
| SP0072 | hypothetical protein |  |  |  | **-1.195** |  |  |  |  |
| SP0082 | cell wall surface anchor family protein |  | **2.243** | **-2.0807** | **-1.304** | **-2.025** |  |  |  |
| SP0085 | ribosomal protein S4 |  |  |  | **-1.144** |  |  |  |  |
| SP0090 | SUGAR-BINDING TRANSPORT PROTEIN. |  |  |  |  | **-1.138** |  |  |  |
| SP0091 | ABC transporter, permease protein | **-1.334** |  |  |  | **-2.35** | **1.661** | **2.324** |  |
| SP0092 | ABC transporter, substrate-binding protein |  |  |  | **1.499** |  | **1.21** | **2.168** |  |
| SP0095 | conserved hypothetical protein |  | **-2.388** | **1.207** |  | **1.029** |  |  | **-1.7905** |
| SP0096 | hypothetical protein | **1.401** |  |  | **1.401** |  |  | **1.216** |  |
| SP0097 | conserved domain protein | **2.452** |  |  | **2.452** |  |  |  |  |
| SP0108 | hypothetical protein |  | **2.037** |  |  |  |  |  | **2.446** |
| SP0109 | bacteriocin, putative |  |  |  |  |  |  | **2.124** |  |
| SP0111 | amino acid ABC transporter, ATP-binding protein, putative |  |  |  |  |  |  | **1.841** |  |
| SP0112 | amino acid ABC transporter, periplasmic amino acid-binding protein, putative |  | **2.3365** | **-1.686** |  |  |  |  | **2.315** |
| SP0113 | argininosuccinate synthase, truncation |  | **2.119** | **-1.1527** |  |  |  |  | **1.8975** |
| SP0114 | hypothetical protein |  |  |  |  |  |  |  | **1.792** |
| SP0116 | hypothetical protein |  |  |  | **-1.974** | **-2.017** |  |  |  |
| SP0119 | MutT-nudix family protein |  |  |  | **-1.118** |  |  |  |  |
| SP0125 | hypothetical protein |  |  |  |  | **-1.156** |  |  |  |
| SP0160 | conserved domain protein | **-1.214** | **2.2035** |  |  |  |  |  |  |
| SP0162 | hypothetical protein | **1.8296** | **5.53** | **2.535** | **4.497** | **-1.438** | **2.989** | **1.433** |  |
| SP0163 | transcriptional regulator PlcR, putative |  |  | **1.132** | **1.505** |  |  |  |  |
| SP0164 | hypothetical protein | **1.9923** | **5.0545** | **4.1417** | **5.548** |  | **4.002** | **2.826** |  |
| SP0165 | flavoprotein | **1.7773** | **5.05** | **4.0297** | **5.667** |  | **4.03** | **2.83** |  |
| SP0166 | pyridoxal-dependent decarboxylase, Orn-Lys-Arg family | **2.109** | **5.31** | **2.972** | **5.054** | **-1.622** | **3.191** | **1.842** |  |
| SP0167 | hypothetical protein | **1.426** | **5.3525** | **2.8017** | **5.207** | **-1.649** | **3.285** | **1.795** |  |
| SP0168 | macrolide efflux protein, putative | **2.2926** | **5.3915** | **3.5535** | **5.225** | **-1.585** | **3.509** | **1.856** |  |
| SP0169 | lactose phosphotransferase system repressor, degenerate | **2.4713** | **5.6545** | **2.977** | **4.837** | **-1.445** | **3.225** | **1.723** |  |
| SP0170 | hypothetical protein | **1.5573** | **5.348** | **2.542** | **4.566** | **-1.594** | **3.111** | **1.593** |  |
| SP0171 | ROK family protein | **2.2907** | **5.7055** | **2.8253** | **4.576** | **-1.595** | **2.939** | **1.479** |  |
| SP0172 | hypothetical protein | **1.7073** | **5.252** | **2.654** | **4.342** | **-1.41** | **2.785** | **1.444** |  |
| SP0173 | DNA mismatch repair protein HexB |  | **3.4595** | **1.4583** | **2.812** |  | **1.14** |  |  |
| SP0175 | 6,7-dimethyl-8-ribityllumazine synthase |  | **-3.7755** |  |  |  |  |  | **-3.9725** |
| SP0183 | hypothetical protein |  |  |  | **-1.141** | **-1.076** |  |  | **1.7067** |
| SP0190 | hypothetical protein |  |  |  |  | **-1.121** |  |  |  |
| SP0191 | hypothetical protein |  |  |  |  | **-1** |  |  |  |
| SP0197 | dihydrofolate synthetase, putative |  |  |  |  | **-1.03** |  |  |  |
| SP0203 | hypothetical protein | **1.0757** |  | **1.22** |  |  |  |  |  |
| SP0211 | ribosomal protein L23 |  |  |  | **-1.007** |  |  |  |  |
| SP0218 | ribosomal protein S17 |  |  |  | **-1.039** |  |  |  |  |
| SP0223 | hypothetical protein |  |  |  | **-1.535** |  |  | **-1.091** | **1.975** |
| SP0231 | adenylate kinase |  |  |  | **-2.214** |  |  |  |  |
| SP0235 | ribosomal protein S11 |  |  |  | **-1.269** |  |  |  |  |
| SP0237 | ribosomal protein L17 |  |  |  | **-1.217** | **-1.27** | **-1.283** |  |  |
| SP0238 | ACT domain protein |  |  |  | **1.434** | **1.338** |  |  |  |
| SP0239 | conserved hypothetical protein |  |  |  | **1.505** | **1.044** |  |  |  |
| SP0240 | phosphoglycerate mutase family protein | **1.4877** |  |  |  |  |  |  |  |
| SP0245 | PYRUVATE FORMATE-LYASE 2 ACTIVATING ENZYME (PFLC). |  |  |  |  | **1.648** |  |  |  |
| SP0266 | glucosamine--fructose-6-phosphate aminotransferase, isomerizing |  |  |  | **-1.915** |  |  |  |  |
| SP0267 | oxidoreductase, putative |  |  |  |  |  |  |  | **-1.539** |
| SP0268 | alkaline amylopullulanase, putative |  |  | **-1.2903** |  |  |  |  | **2.3375** |
| SP0284 | PTS system, mannose-specific IIAB components |  |  |  | **-1.221** |  |  |  |  |
| SP0285 | alcohol dehydrogenase, zinc-containing |  |  |  | **2.437** |  |  | **1.804** | **1.7515** |
| SP0286 | Cof family protein |  |  |  |  |  |  |  |  |
| SP0287 | xanthine-uracil permease family protein |  |  | **1.6973** |  | **1.573** |  |  | **-2.152** |
| SP0288 | conserved hypothetical protein |  |  |  |  |  |  |  | **-2.018** |
| SP0298 | conserved hypothetical protein |  |  |  | **2.036** |  |  |  |  |
| SP0300 | IS630-Spn1, transposase Orf2 |  |  |  |  |  |  |  |  |
| SP0301 | glycosyl hydrolase, family 1, truncation | **-1.034** |  |  |  |  |  |  |  |
| SP0307 | PTS system, IIA component |  |  |  | **1.09** |  |  |  |  |
| SP0309 | hypothetical protein |  | **1.5746** |  |  |  |  |  | **1.7015** |
| SP0313 | BSAA. |  |  |  |  | **1.959** |  |  |  |
| SP0314 | hyaluronidase |  |  |  |  | **-1.578** |  |  |  |
| SP0322 | UNSATURATED GLUCURONYL HYDROLASE. |  |  |  |  | **1.054** |  |  |  |
| SP0323 | PTS system, IIB component |  |  |  |  |  |  | **1.166** |  |
| SP0324 | PTS system, IIC component |  |  |  |  |  |  | **1.16** |  |
| SP0332 | hypothetical protein |  |  |  |  | **1.045** |  |  |  |
| SP0333 | YORF[A,B,C,D,E], FTSL, PBPX AND REGR GENES. |  |  |  |  |  |  |  |  |
| SP0346 | capsular polysaccharide biosynthesis protein Cps4A | **-1.313** | **2.998** | **-1.793** | **-1.825** |  |  |  |  |
| SP0347 | capsular polysaccharide biosynthesis protein Cps4B |  | **2.5105** |  |  |  |  |  |  |
| SP0348 | capsular polysaccharide biosynthesis protein Cps4C |  | **2.5795** | **-1.2377** | **-1.711** | **-1.046** |  |  |  |
| SP0349 | capsular polysaccharide biosynthesis protein Cps4D |  | **2.3355** | **-1.886** | **-2.097** |  |  |  |  |
| SP0350 | capsular polysaccharide biosynthesis protein Cps4E | **-1.5377** |  | **-1.225** | **-1.544** |  |  | **-1.029** |  |
| SP0351 | capsular polysaccharide biosynthesis protein Cps4F | **-1.509** | **2.3675** | **-1.0535** | **-1.57** |  |  |  |  |
| SP0364 | IS66 family element, Orf2, interruption |  |  |  | **-1.262** | **-1.322** |  |  |  |
| SP0365 | IS66 family element, Orf1, authentic frameshift |  | **1.909** |  |  | **1.058** |  |  |  |
| SP0368 | cell wall surface anchor family protein, authentic frameshift |  |  |  |  |  |  | **1.661** |  |
| SP0380 | hypothetical protein |  | **1.6775** |  |  |  |  |  |  |
| SP0385 | conserved hypothetical protein |  |  |  | **1.157** |  |  |  |  |
| SP0386 | sensor histidine kinase, putative |  |  |  | **1.046** |  |  |  |  |
| SP0387 | DNA-binding response regulator |  |  |  | **1.156** |  |  |  |  |
| SP0388 | hypothetical protein, authentic frameshift |  |  |  | **1.117** |  |  |  |  |
| SP0389 | hypothetical protein |  |  |  | **1.021** |  |  |  |  |
| SP0390 | choline binding protein G |  |  |  | **1.009** |  |  |  |  |
| SP0394 | PTS system, mannitol-specific IIBC components |  |  |  | **2.209** |  |  | **3.931** |  |
| SP0395 | transcriptional regulator, putative | **-1.227** |  |  |  |  |  | **2.713** |  |
| SP0396 | PTS system, mannitol-specific IIA component |  |  |  | **2.21** |  |  | **3.833** |  |
| SP0397 | mannitol-1-phosphate 5-dehydrogenase |  |  |  | **1.417** |  |  | **2.808** |  |
| SP0398 | hypothetical protein |  |  |  |  |  |  | **1.397** |  |
| SP0399 | glutamine ABC transporter, ATP-binding protein, truncation |  |  |  |  |  |  | **3.34** |  |
| SP0408 | sodium:alanine symporter family protein |  |  |  | **1.183** |  |  |  |  |
| SP0413 | PRECURSOR MONOFUNCTIONAL ASPARTOKINASE. |  |  |  |  | **1.856** |  |  |  |
| SP0418 | acyl carrier protein |  |  |  | **-1.825** |  |  |  |  |
| SP0419 | enoyl-(acyl-carrier-protein) reductase |  | **-1.7161** |  | **-3.758** |  |  |  |  |
| SP0420 | malonyl CoA-acyl carrier protein transacylase |  | **-1.6641** |  | **-3.857** |  |  |  |  |
| SP0422 | 3-oxoacyl-(acyl-carrier-protein) synthase II |  | **-1.5134** |  | **-3.772** |  |  |  |  |
| SP0424 | (3R)-hydroxymyristoyl-(acyl-carrier-protein) dehydratase |  |  |  | **-3.354** |  |  |  |  |
| SP0426 | acetyl-CoA carboxylase, carboxyl transferase subunit beta |  | **-1.5965** |  | **-3.813** |  |  |  |  |
| SP0427 | acetyl-CoA carboxylase, carboxyl transferase subunit alpha |  | **-1.8468** |  | **-3.454** |  |  |  |  |
| SP0428 | hypothetical protein |  |  |  | **-3.443** |  |  |  |  |
| SP0429 | hypothetical protein |  |  |  | **-3.205** |  |  |  |  |
| SP0430 | hypothetical protein |  |  |  | **-3.094** |  |  |  |  |
| SP0431 | conserved domain protein |  |  |  | **-2.631** |  |  |  |  |
| SP0451 | hypothetical protein |  |  |  |  | **1.508** |  |  |  |
| SP0455 | hypothetical protein |  |  |  |  |  |  |  | **2.0565** |
| SP0459 | formate acetyltransferase |  |  |  | **2.157** | **-1.08** |  | **1.958** |  |
| SP0461 | transcriptional regulator, putative | **2.396** | **1.8545** | **3.167** | **2.986** | **1.381** | **2.592** |  |  |
| SP0462 | cell wall surface anchor family protein | **3.2747** | **2.435** | **2.9047** | **2.914** | **1.3** | **3.133** | **1.255** | **2.0005** |
| SP0463 | cell wall surface anchor family protein | **3.502** | **1.6755** | **3.8203** | **2.999** | **1.3** | **3.291** | **1.438** | **1.687** |
| SP0464 | cell wall surface anchor family protein | **2.9923** | **1.7875** | **3.4883** | **2.794** | **1.063** | **3.088** | **1.354** | **1.822** |
| SP0466 | sortase, putative | **2.062** | **3.442** | **1.9607** | **1.326** |  | **2.033** |  | **2.584** |
| SP0467 | sortase, putative | **1.718** | **2.3835** | **1.952** | **1.405** |  | **2.053** |  | **1.766** |
| SP0468 | sortase, putative | **1.6676** | **2.0145** | **1.673** |  |  | **1.398** |  |  |
| SP0474 | PTS system, cellobiose-specific IIC component |  | **1.571** |  |  |  |  |  |  |
| SP0475 | hypothetical protein |  | **2.087** |  |  |  |  |  |  |
| SP0486 | RNA methyltransferase, TrmH family |  |  |  | **-1.128** |  |  |  |  |
| SP0497 | hypothetical protein |  | **2.3765** | **1.04** |  |  |  |  |  |
| SP0498 | endo-beta-N-acetylglucosaminidase, putative |  | **2.6625** |  |  |  |  |  | **2.549** |
| SP0502 | glutamine synthetase, type I |  | **-1.5205** |  |  |  |  |  | **-1.7615** |
| SP0504 | hypothetical protein |  | **-1.6705** |  | **-1.215** | **-1.055** |  |  | **-2.254** |
| SP0505 | type I restriction-modification system, S subunit, putative |  |  |  | **-1.047** | **-1.136** |  |  |  |
| SP0507 | type I restriction-modification system, S subunit, putative |  |  |  |  |  |  | **-1.131** |  |
| SP0514 | hypothetical protein | **-1.12** |  |  | **-1.228** |  |  |  |  |
| SP0515 | heat-inducible transcription repressor HrcA |  |  | **1.221** |  |  |  |  |  |
| SP0520 | hypothetical protein |  |  |  | **1.316** |  |  |  |  |
| SP0521 | HIT family protein |  |  | **1.0637** | **1.179** | **1.799** |  |  |  |
| SP0528 | peptide pheromone BlpC |  |  |  | **1.103** |  |  |  |  |
| SP0531 | bacteriocin BlpI | **-1.11** | **2.198** |  |  | **-2.211** |  |  | **1.7145** |
| SP0532 | bacteriocin BlpJ |  |  |  |  | **-1.944** |  |  |  |
| SP0534 | hypothetical protein | **-1.311** |  |  |  | **-1.88** |  |  |  |
| SP0535 | hypothetical protein |  | **2.151** | **1.5115** |  | **-1.105** |  |  |  |
| SP0536 | immunity protein BlpL |  | **2.3505** |  |  |  |  |  |  |
| SP0540 | blpN protein |  |  |  | **1.092** |  |  |  |  |
| SP0542 | hypothetical protein |  |  | **1.514** |  |  |  |  |  |
| SP0555 | ribosomal protein L7A family |  |  |  | **-1.03** |  |  |  |  |
| SP0557 | ribosome-binding factor A |  |  |  | **-1.089** |  |  |  |  |
| SP0573 | hypothetical protein |  |  | **1.5215** |  |  |  |  |  |
| SP0575 | helicase, putative |  |  |  |  | **1.141** |  |  |  |
| SP0576 | transcription antiterminator Lict | **-1.086** |  | **-1.263** |  |  |  | **7.413** |  |
| SP0578 | 6-phospho-beta-glucosidase |  | **1.6424** |  |  | **-1.121** |  | **6.588** |  |
| SP0583 | IS1239, transposase, putative, degenerate |  |  |  | **-1.105** |  |  |  |  |
| SP0584 | transcriptional regulator, putative |  |  |  | **-1.462** |  |  |  |  |
| SP0585 | 5-methyltetrahydropteroyltriglutamate--homocysteine methyltransferase |  | **2.11** |  |  |  |  |  | **2.625** |
| SP0587 | hypothetical protein | **-1.038** |  |  |  |  |  |  |  |
| SP0594 | hypothetical protein, fusion |  |  |  | **-1.52** |  |  |  |  |
| SP0595 | hypothetical protein |  |  |  | **-1.641** |  |  |  |  |
| SP0596 | hypothetical protein |  |  |  | **-1.452** |  |  |  |  |
| SP0598 | hypothetical protein |  |  |  | **-1.292** |  |  |  |  |
| SP0599 | transmembrane protein Vexp1 |  |  |  | **-1.181** |  |  |  |  |
| SP0602 | pep27 protein |  | **1.3145** |  |  |  |  |  |  |
| SP0620 | amino acid ABC transporter, amino acid-binding protein, putative |  |  |  |  |  |  |  | **2.078** |
| SP0623 | dipeptidase |  |  |  | **1.058** |  |  |  |  |
| SP0625 | conserved hypothetical protein, authentic point mutation |  |  | **-2.0063** |  |  |  |  |  |
| SP0633 | hypothetical protein |  |  | **-1.1657** |  |  |  |  |  |
| SP0647 | PTS system, IIC component, putative |  |  |  |  |  |  | **2.42** |  |
| SP0648 | beta-galactosidase |  |  |  |  |  |  | **2.802** | **2.232** |
| SP0649 | conserved hypothetical protein, degenerate |  |  |  | **1.652** |  |  |  |  |
| SP0651 | conserved hypothetical protein |  |  |  | **1.026** |  |  |  |  |
| SP0658 | cytochrome c-type biogenesis protein CcdA |  | **2..205** |  |  |  |  |  | **1.5327** |
| SP0660 | peptide methionine sulfoxide reductase |  | **1.9925** |  |  |  |  |  |  |
| SP0676 | transcriptional regulator, putative |  |  |  |  |  |  |  | **2.1935** |
| SP0677 | conserved hypothetical protein |  |  |  |  | **1.318** |  |  |  |
| SP0678 | conserved hypothetical protein |  |  |  |  | **1.21** |  |  |  |
| SP0683 | hypothetical protein |  |  |  |  |  |  | **1.215** |  |
| SP0686 | conserved hypothetical protein | **-1.205** |  |  |  |  |  |  |  |
| SP0692 | hypothetical protein |  |  |  |  | **-1.173** | **-1.496** |  |  |
| SP0693 | hypothetical protein |  |  |  |  |  | **-1.291** |  |  |
| SP0701 | OROTIDINE 5-PHOSPHATE DECARBOXYLASE (EC 4.1.1.23) |  |  |  |  | **1.488** |  |  |  |
| SP0702 | OROTATE PHOSPHORIBOSYLTRANSFERASE PYRE. |  |  |  |  | **1.211** |  |  |  |
| SP0703 | hypothetical protein |  |  |  | **2.238** |  |  | **2.047** |  |
| SP0704 | hypothetical protein |  |  |  | **1.424** |  |  |  |  |
| SP0706 | hypothetical protein | **-1.3757** |  |  | **1.056** |  |  | **1.995** |  |
| SP0709 | amino acid ABC transporter, ATP-binding protein |  |  |  | **1.12** |  |  |  |  |
| SP0710 | amino acid ABC transporter, permease protein |  |  |  | **1.065** |  |  |  |  |
| SP0711 | amino acid ABC transporter, permease protein | **-1.456** |  |  | **1.482** |  |  |  |  |
| SP0712 | lactate oxidase, truncation |  |  |  | **2.305** |  |  | **2.063** |  |
| SP0715 | lactate oxidase |  |  |  | **2.267** |  |  | **2.076** |  |
| SP0717 | hydroxyethylthiazole kinase |  | **-2.1215** | **-1.472** | **-1.652** | **-1.009** |  |  | **-2.29** |
| SP0718 | thiamine-phosphate pyrophosphorylase |  | **-2.214** |  | **-1.653** |  |  |  | **-2.3205** |
| SP0719 | conserved hypothetical protein |  |  |  |  |  |  |  | **-1.727** |
| SP0720 | ABC transporter, ATP-binding protein |  |  |  |  |  |  |  | **-1.7285** |
| SP0721 | conserved hypothetical protein |  |  |  |  |  |  |  | **-1.887** |
| SP0722 | transcriptional activator TenA |  |  |  |  |  |  |  | **-2.1465** |
| SP0723 | conserved domain protein |  |  |  | **-1.056** |  |  |  | **-1.7455** |
| SP0726 | THID |  |  |  |  | **-1.142** |  |  |  |
| SP0727 | transcriptional repressor, putative |  |  |  |  |  |  |  | **2.2205** |
| SP0728 | hypothetical protein |  |  |  |  | **-2.053** |  |  |  |
| SP0729 | P-TYPE ATPASE. |  |  |  |  | **-1.374** |  |  |  |
| SP0730 | pyruvate oxidase |  |  |  | **1.518** |  |  |  |  |
| SP0735 | 6-phospho-beta-glucosidase, truncation | **-1.0227** | **2.012** |  |  |  |  |  |  |
| SP0738 | conserved domain protein |  |  |  | **-1.356** |  |  |  |  |
| SP0742 | conserved hypothetical protein |  | **-1.5145** |  | **-2.873** | **1.161** |  |  |  |
| SP0743 | HYPOTHETICAL PROTEIN HOMOLOGOUS TO SPT02287 |  |  |  |  | **1.68** |  |  |  |
| SP0759 | hypothetical protein |  |  |  | **-1.046** |  |  |  |  |
| SP0760 | hypothetical protein |  |  |  | **-1.061** |  |  |  |  |
| SP0772 | hypothetical protein |  | **1.848** |  |  |  |  |  |  |
| SP0774 | HYPOTHETICAL PROTEIN HOMOLOGOUS TO SPT02325 |  |  |  |  | **1.119** |  |  |  |
| SP0776 | KH domain protein |  |  |  | **-1.692** |  |  |  |  |
| SP0781 | HYPOTHETICAL PROTEIN HOMOLOGOUS TO SPT02375 |  |  |  |  | **1.393** |  |  |  |
| SP0783 | conserved hypothetical protein |  | **-1.5275** |  | **-3.187** | **-1.554** |  |  |  |
| SP0798 | DNA-binding response regulator CiaR |  |  |  | **2.104** | **-3.102** |  |  |  |
| SP0799 | sensor histidine kinase CiaH |  |  |  | **2.014** | **-3.237** |  |  |  |
| SP0816 | HYPOTHETICAL PROTEIN HOMOLOGOUS TO SPT02375 |  |  |  |  | **1.217** |  |  |  |
| SP0820 | ATP-dependent Clp protease, ATP-binding subunit ClpE | **1.0567** |  |  |  |  |  |  |  |
| SP0822 | conserved hypothetical protein |  |  | **1.6103** |  |  |  |  |  |
| SP0825 | methylenetetrahydrofolate dehydrogenase-methenyltetrahydrofolate cyclohydrolase |  |  |  | **-1.238** |  | **-1.21** | **-1.153** |  |
| SP0842 | pyrimidine-nucleoside phosphorylase | **-1.1413** |  |  |  |  |  |  |  |
| SP0851 | conserved hypothetical protein |  |  |  | **-2.003** |  |  |  |  |
| SP0861 | hypothetical protein |  |  | **1.6483** |  |  |  |  |  |
| SP0867 | ABC transporter, ATP-binding protein |  | **-1.6055** |  |  |  |  |  |  |
| SP0875 | TRANSCRIPTIONAL REGULATOR (DEOR FAMILY). |  |  |  |  | **1.052** |  |  |  |
| SP0879 | hypothetical protein |  |  |  | **2.964** |  |  |  |  |
| SP0883 | hypothetical protein |  |  | **-1.2093** |  |  |  |  |  |
| SP0884 | hypothetical protein |  | **2.624** | **-1.2463** |  |  |  |  |  |
| SP0885 | hypothetical protein |  | **2.6445** | **-1.721** |  |  |  |  | **2.566** |
| SP0911 | hypothetical protein |  |  |  | **2.051** |  | **1.404** | **1.905** |  |
| SP0912 | ABC transporter, ATP-binding protein |  | **1.807** |  |  |  |  |  |  |
| SP0913 | ABC transporter, permease protein, putative |  | **1.807** |  |  |  |  |  |  |
| SP0914 | nodulin-related protein, truncation |  | **1.513** |  |  |  |  |  |  |
| SP0915 | IS1239, transposase, putative |  |  |  | **-1.383** |  |  |  |  |
| SP0916 | lysine decarboxylase |  |  |  | **-2.61** |  |  |  |  |
| SP0917 | pilin gene inverting-related protein |  |  |  | **-1.102** |  |  | **-1.05** |  |
| SP0918 | spermidine synthase |  |  |  | **-2.29** |  |  |  |  |
| SP0919 | conserved hypothetical protein |  |  |  | **-2.264** |  |  |  |  |
| SP0920 | carboxynorspermidine decarboxylase |  |  |  | **-2.237** |  |  |  |  |
| SP0921 | conserved hypothetical protein |  |  |  | **-2.39** |  |  |  |  |
| SP0923 | Cof family protein |  |  |  |  | **1.003** |  |  |  |
| SP0934 | hypothetical protein |  |  |  | **-1.149** |  |  |  |  |
| SP0952 | alanine dehydrogenase, authentic frameshift |  | **2.0985** |  |  |  |  |  |  |
| SP0953 | acetyltransferase, GNAT family |  |  |  | **-1.284** |  |  |  |  |
| SP0954 | competence protein CelA |  | **3.31** |  |  |  |  |  |  |
| SP0955 | competence protein CelB | **-1.046** | **3.273** | **-1.9** | **-1.075** | **-1.077** |  |  |  |
| SP0956 | hypothetical protein | **-1.23** |  | **-1.4283** |  |  |  | **1.982** |  |
| SP0957 | ABC transporter, ATP-binding protein | **-1.322** |  | **-1.0477** | **1.034** |  |  | **2.122** |  |
| SP0958 | hypothetical protein | **-1.2777** |  | **-1.4947** |  |  |  | **2.018** | **1.6535** |
| SP0959 | translation initiation factor IF-3 |  |  |  | **-1.603** |  |  |  |  |
| SP0960 | ribosomal protein L35 |  |  |  | **-1.048** |  |  |  |  |
| SP0963 | dihydroorotate dehydrogenase, electron transfer subunit |  |  |  | **1.371** |  |  | **1.6** |  |
| SP0973 | ribosomal protein L33 |  |  |  | **-1.129** |  |  |  |  |
| SP0978 | competence protein CoiA |  |  |  |  | **-1.87** |  |  |  |
| SP0981 | protease maturation protein, putative |  |  |  | **1.363** |  |  |  |  |
| SP0983 | IS630-Spn1, transposase Orf2, degenerate |  |  |  | **-1.664** | **-1.455** |  |  |  |
| SP0987 | hypothetical protein |  |  |  |  | **1.127** |  |  |  |
| SP1002 | adhesion lipoprotein |  | **1.4426** |  | **-2.892** |  |  |  | **1.699** |
| SP1003 | conserved hypothetical protein |  |  |  | **-2.714** |  |  | **1.048** |  |
| SP1005 | HYPOTHETICAL PROTEIN HOMOLOGOUS TO SPT02608 |  |  |  |  | **-1.044** |  |  |  |
| SP1009 | ferrochelatase | **-2.1255** |  |  |  | **1.128** |  |  |  |
| SP1011 | GtrA family protein |  |  | **1.1063** |  | **1.513** |  |  |  |
| SP1012 | conserved hypothetical protein |  |  |  | **-1.387** |  |  |  |  |
| SP1017 | 4-oxalocrotonate tautomerase |  |  | **1.0733** |  | **1.507** |  |  |  |
| SP1027 | conserved hypothetical protein |  |  |  |  | **-3.473** |  |  |  |
| SP1028 | hypothetical protein |  |  |  |  | **-1.852** |  |  |  |
| SP1029 | RNA methyltransferase, TrmA family |  |  |  |  | **-1.404** |  |  |  |
| SP1030 | hypothetical protein |  |  |  |  | **20566** |  |  |  |
| SP1031 | hypothetical protein |  |  |  |  | **1.198** |  |  |  |
| SP1035 | iron-compound ABC transporter, ATP-binding protein |  |  |  | **-1.609** |  |  |  |  |
| SP1036 | hypothetical protein |  |  |  | **-1.161** |  |  | **-1.12** |  |
| SP1037 | type II restriction endonuclease, putative |  |  | **-1.3627** | **-2.6** | **-1.228** |  |  |  |
| SP1038 | hypothetical protein |  |  | **-1.407** | **-2.157** | **-1.269** |  |  |  |
| SP1039 | hypothetical protein |  |  | **-1.5783** | **-2.125** | **-2.153** |  |  |  |
| SP1040 | site-specific recombinase, resolvase family |  |  | **-1.2777** |  |  |  |  |  |
| SP1043 | hypothetical protein |  | **1.5576** |  |  |  |  |  |  |
| SP1049 | hypothetical protein |  |  | **1.1155** |  |  |  |  |  |
| SP1050 | transcriptional regulator, putative |  |  | **1.2457** |  | **2.13** |  |  |  |
| SP1057 | transcriptional regulator PlcR, putative | **2.5703** | **3.151** | **4.055** | **4.095** |  | **2.483** | **1.731** |  |
| SP1058 | hypothetical protein | **2.4137** | **4.804** | **4.3737** | **4.871** |  | **4.101** | **3.42** |  |
| SP1059 | hypothetical protein | **2.973** | **4.144** | **4.6083** | **5.236** | **-1.273** | **4.381** | **4.179** |  |
| SP1060 | hypothetical protein | **2.3627** | **5.562** | **3.5973** | **6.122** |  | **4.249** | **3.439** |  |
| SP1061 | protein kinase, putative | **2.2757** | **5.4175** | **3.3457** | **6.066** |  | **4.054** | **3.336** |  |
| SP1062 | ABC transporter, ATP-binding protein | **2.344** | **5.6935** | **3.3873** | **5.938** |  | **4.111** | **3.507** |  |
| SP1063 | ABC-2 transporter, permease protein, putative | **2.2853** | **5.389** | **3.5983** | **5.827** | **-1.08** | **3.823** | **3.478** |  |
| SP1066 | IS1381, transposase OrfA, truncation |  | **3.0235** |  | **1.539** |  |  |  |  |
| SP1069 | conserved hypothetical protein |  |  |  | **-1.23** |  |  |  |  |
| SP1072 | DNA primase |  |  | **-1.048** |  |  |  |  |  |
| SP1097 | conserved hypothetical protein |  |  | **1.073** |  |  |  |  |  |
| SP1102 | conserved hypothetical protein TIGR00103 |  |  | **1.5303** |  |  |  |  |  |
| SP1103 | hypothetical protein |  |  | **1.3973** |  |  |  |  |  |
| SP1105 | ribosomal protein L21 |  |  | **1.0823** |  |  |  |  |  |
| SP1112 | degV family protein |  |  | **1.0527** |  |  |  |  |  |
| SP1113 | DNA-binding protein HU |  |  | **2.6546** |  |  |  |  |  |
| SP1116 | transporter, putative |  |  | **-1.392** |  |  |  |  |  |
| SP1119 | NADP-DEPENDENT GLYCERALDEHYDE-3-PHOSPHATE DEHYDROGENASE (EC 1.2.1.9) |  |  |  |  | **1.128** |  |  |  |
| SP1121 | 1,4-alpha-glucan branching enzyme |  |  |  | **1.733** |  |  | **2.385** | **1.796** |
| SP1124 | glycogen synthase |  |  |  | **1.171** |  |  | **2.578** |  |
| SP1125 | phosphoserine phosphatase SerB, degenerate |  |  |  |  |  |  | **1.578** | **2.318** |
| SP1126 | conserved hypothetical protein TIGR00045 |  |  | **-1.215** |  |  |  | **1.279** |  |
| SP1127 | hypothetical protein |  |  |  | **1.068** |  |  |  |  |
| SP1142 | hypothetical protein |  |  | **1.0173** |  |  |  |  |  |
| SP1143 | conserved hypothetical protein |  |  |  |  |  | **1.005** |  |  |
| SP1148 | IS630-Spn1, transposase Orf2 |  |  |  | **-1.534** | **-1.136** |  |  |  |
| SP1158 | hypothetical protein |  |  |  | **-1.34** | **-1.68** |  | **-1.222** |  |
| SP1162 | acetoin dehydrogenase complex, E2 component, dihydrolipoamide acetyltransferase, putative |  |  |  | **-1.052** |  |  |  |  |
| SP1163 | acetoin dehydrogenase, E1 component, beta subunit, putative |  |  |  | **-1.056** |  |  | **-1.014** |  |
| SP1165 | hypothetical protein |  |  |  | **-1.115** |  |  |  |  |
| SP1170 | hypothetical protein |  |  |  |  |  |  |  |  |
| SP1171 | hydrolase, haloacid dehalogenase-like family |  |  | **1.1043** |  |  |  |  |  |
| SP1185 | PTS system, lactose-specific IIBC components | **-1.573** | **2.9685** | **-2.3145** |  | **-3.936** |  | **3.61** |  |
| SP1186 | PTS system, lactose-specific IIA component | **-1.3163** |  |  |  | **-2.679** |  | **3.854** |  |
| SP1187 | transcription antiterminator LacT | **-1.4477** |  | **-1.553** |  | **-4.549** |  | **3.2** |  |
| SP1188 | hypothetical protein |  |  |  |  |  |  | **1.182** |  |
| SP1189 | hypothetical protein |  |  |  | **1.025** |  |  | **1.367** |  |
| SP1197 | conserved hypothetical protein |  |  |  |  |  |  | **1.868** |  |
| SP1198 | hypothetical protein |  |  |  |  |  |  | **1.635** |  |
| SP1199 | hypothetical protein |  |  |  |  |  |  | **1.821** | **1.524** |
| SP1214 | O-acetylhomoserine sulfhydrylase, truncation |  |  |  |  | **1.255** |  |  |  |
| SP1215 | transporter, FNT family, putative |  |  | **-1.0163** | **1.566** |  |  | **1.128** | **1.846** |
| SP1216 | hypothetical protein |  |  |  | **1.417** |  |  |  | **1.723** |
| SP1220 | L-lactate dehydrogenase |  |  |  | **1.021** |  |  |  |  |
| SP1229 | formate--tetrahydrofolate ligase |  |  | **1.145** | **1.594** | **1.824** |  |  |  |
| SP1230 | HYPOTHETICAL PROTEIN HOMOLOGOUS TO SPT02857 |  |  |  |  | **1.204** |  |  |  |
| SP1231 | flavoprotein |  |  |  | **1.608** |  |  |  |  |
| SP1242 | amino acid ABC transporter, ATP-binding protein |  |  |  | **-1.458** |  |  |  |  |
| SP1249 | conserved hypothetical protein |  |  | **1.778** | **1.655** | **1.003** |  |  |  |
| SP1251 | endonuclease, putative |  | **1.978** |  |  |  |  |  |  |
| SP1256 | conserved hypothetical protein |  |  | **-1.045** |  |  |  |  |  |
| SP1264 | conserved domain protein |  |  |  | **-1.066** |  |  |  |  |
| SP1266 | DNA processing protein DprA, putative |  | **2.3265** | **-1.7163** |  | **-1.542** |  |  |  |
| SP1267 | licC protein |  |  |  | **2.212** |  |  |  |  |
| SP1268 | licB protein |  |  |  | **2.372** |  |  |  |  |
| SP1270 | alcohol dehydrogenase, zinc-containing |  |  |  | **2.284** | **-1.184** |  |  |  |
| SP1271 | HYPOTHETICAL PROTEIN HOMOLOGOUS TO SPT02904 |  |  |  |  | **-1.386** |  |  |  |
| SP1275 | carbamoyl-phosphate synthase, large subunit |  |  | **1.439** | **1.718** |  |  | **1.468** |  |
| SP1278 | pyrimidine operon regulatory protein |  |  | **1.8913** | **1.139** | **1.675** |  | **1.523** |  |
| SP1281 | hypothetical protein |  |  |  | **1.108** |  |  |  |  |
| SP1282 | ABC transporter, ATP-binding protein |  |  |  |  | **2.492** |  |  |  |
| SP1284 | lemA protein |  |  |  |  | **1.071** |  |  |  |
| SP1286 | uracil permease |  |  |  |  | **1.52** |  |  |  |
| SP1296 | HYPOTHETICAL CHORISMATE MUTASE PROTEIN HOMOLOGOUS TO SPT02933 |  |  |  |  | **1.088** |  |  |  |
| SP1300 | HYPOTHETICAL PROTEIN HOMOLOGOUS TO SPT02939 |  |  |  |  | **1.146** |  |  |  |
| SP1322 | v-type sodium ATP synthase, subunit I |  |  |  | **1.497** |  |  |  |  |
| SP1323 | hypothetical protein |  | **2.6385** |  |  |  |  |  |  |
| SP1326 | neuraminidase, putative |  |  |  | **1.018** |  |  |  |  |
| SP1327 | conserved hypothetical protein |  |  |  | **1.239** |  |  |  |  |
| SP1339 | hypothetical protein |  |  | **1.5613** | **1.918** | **1.018** |  | **2.132** |  |
| SP1340 | hypothetical protein |  |  |  | **1.809** |  |  | **1.49** |  |
| SP1341 | ABC transporter, ATP-binding protein |  |  |  | **1.875** |  |  | **1.474** |  |
| SP1342 | toxin secretion ABC transporter, ATP-binding-permease protein |  |  |  | **2.248** |  |  | **1.622** |  |
| SP1343 | prolyl oligopeptidase family protein |  |  |  | **2.714** |  |  | **1.903** |  |
| SP1345 | hypothetical protein |  |  | **1.7203** | **2.046** | **1.113** |  | **2.001** |  |
| SP1353 | hypothetical protein |  |  | **1.0233** | **-1.629** | **-2.081** |  | **-1.007** |  |
| SP1401 | hypothetical protein |  |  |  |  | **1.079** |  | **-1.387** |  |
| SP1405 | conserved hypothetical protein |  |  | **1.0843** |  | **1.232** |  |  |  |
| SP1414 | ribosomal protein S21 |  |  | **1.528** |  |  |  |  |  |
| SP1425 | hypothetical protein |  |  |  |  | **1.261** |  |  |  |
| SP1426 | ABC transporter, ATP-binding protein |  |  |  |  | **1.936** |  |  |  |
| SP1427 | peptidase, U32 family |  |  |  | **-1.563** |  |  |  |  |
| SP1430 | type II restriction endonuclease, putative, authentic point mutation |  | **-1.732** |  |  |  |  |  |  |
| SP1435 | ABC transporter, ATP-binding protein |  | **1.915** |  |  |  |  |  |  |
| SP1436 | hypothetical protein |  | **2.2975** |  |  |  |  |  |  |
| SP1437 | conserved domain protein |  | **1.6258** |  |  |  |  |  |  |
| SP1442 | IS66 family element, Orf2 | **-1.545** | **2.0185** |  |  |  |  |  |  |
| SP1445 | GMP synthase |  |  | **1.173** |  |  |  |  |  |
| SP1446 | transcriptional regulator, GntR family |  |  |  |  | **1.052** |  |  |  |
| SP1455 | hypothetical protein |  |  |  |  | **1.755** |  |  | **1.756** |
| SP1464 | acetyltransferase, GNAT family |  |  |  |  | **1.067** |  |  |  |
| SP1465 | HYPOTHETICAL PROTEIN HOMOLOGOUS TO SPT03378 |  |  |  |  | **2.259** |  |  |  |
| SP1466 | hemolysin |  |  |  | **-2.281** | **1.593** |  |  |  |
| SP1467 | conserved hypothetical protein |  |  |  | **1.047** |  |  |  |  |
| SP1468 | pyridoxine biosynthesis protein |  |  |  | **1.13** |  |  |  |  |
| SP1470 | thiamine biosynthesis protein ApbE, putative |  |  |  |  |  |  | **1.633** | **2.656** |
| SP1471 | oxidoreductase, putative |  |  |  | **1.095** |  |  | **1.675** | **2.1515** |
| SP1472 | oxidoreductase, putative |  |  |  |  |  |  | **1.878** | **2.1565** |
| SP1502 | amino acid ABC transporter, permease protein |  |  |  |  | **1.414** |  |  |  |
| SP1515 | IS1239, transposase, putative, degenerate |  |  |  | **-1.442** |  |  |  |  |
| SP1527 | oligopeptide ABC transporter, oligopeptide-binding protein AliB |  |  | **1.648** | **1.299** | **1.992** |  |  |  |
| SP1528 | hypothetical protein | **1.4543** |  | **2.783** |  | **2.328** |  |  |  |
| SP1535 | conserved hypothetical protein |  |  |  | **-1.172** |  |  |  |  |
| SP1572 | non-heme iron-containing ferritin |  |  | **1.157** |  |  |  |  |  |
| SP1580 | sugar ABC transporter, ATP-binding protein |  |  |  | **1.069** |  |  | **1.328** |  |
| SP1585 | hypothetical protein |  |  |  |  | **1.693** |  |  |  |
| SP1587 | oxalate:formate antiporter |  | **-1.53** |  | **1.353** |  |  |  | **-2.9795** |
| SP1588 | oxidoreductase, pyridine nucleotide-disulfide, class I |  |  |  | **1.331** |  |  |  |  |
| SP1601 | conserved hypothetical protein |  |  |  | **-1.326** |  |  |  |  |
| SP1605 | ferredoxin |  |  | **1.435** |  |  |  |  |  |
| SP1612 | conserved domain protein | **1.491** |  | **1.049** |  |  |  |  |  |
| SP1613 | IS3-Spn1, transposase, authentic point mutation |  | **1.5715** |  |  |  |  |  |  |
| SP1615 | transketolase, authentic frameshift |  | **1.831** |  |  |  |  |  |  |
| SP1616 | ribulose-phosphate 3-epimerase family protein |  | **2.2675** |  |  |  |  |  |  |
| SP1620 | PTS system, nitrogen regulatory component IIA, putative |  | **2.3135** |  | **-1.003** |  |  |  |  |
| SP1621 | transcription antiterminator BglG family protein, authentic frameshift | **-1.1123** |  | **-1.6745** |  |  |  |  |  |
| SP1622 | transposase, IS200 family | **1.694** | **4.57** | **2.6913** | **3.317** |  | **2.271** | **1.818** |  |
| SP1623 | cation-transporting ATPase, E1-E2 family |  |  |  | **-1.128** |  |  |  |  |
| SP1624 | acyltransferase family protein |  |  |  |  | **1.91** |  |  |  |
| SP1625 | cadmium resistance transporter, putative |  |  | **1.3407** |  |  |  |  |  |
| SP1632 | Histidine kinase |  |  |  | **1.052** |  |  |  |  |
| SP1633 | DNA-binding response regulator |  | **1.3825** |  | **1.033** |  |  |  |  |
| SP1635 | hypothetical protein |  |  |  |  | **1.434** |  |  |  |
| SP1640 | hypothetical protein |  | **1.257** |  |  | **1.037** |  |  | **1.08525** |
| SP1651 | thiol peroxidase |  |  | **1.036** |  | **1.344** |  |  | **-1.9205** |
| SP1655 | PHOSPHOGLYCEROMUTASE. |  |  |  |  | **1.098** |  |  |  |
| SP1676 | N-acetylneuraminate lyase, putative |  |  |  |  |  |  | **1.845** |  |
| SP1677 | hypothetical protein |  |  | **-1.4477** |  | **-1.094** |  | **2.084** | **1.519** |
| SP1678 | hypothetical protein | **-1.059** |  | **-1.4403** |  | **-1.324** |  | **2.211** | **1.6357** |
| SP1679 | hypothetical protein | **-1.0817** |  |  |  |  |  | **2.794** |  |
| SP1680 | conserved hypothetical protein |  |  | **-1.0587** |  | **-1.214** |  | **2.257** | **2.32** |
| SP1681 | sugar ABC transporter, permease protein |  |  |  |  |  |  | **2.085** | **1.653** |
| SP1682 | SUGAR ABC TRANSPORTER, PERMEASE PROTEIN. |  |  |  |  | **-1.001** |  |  |  |
| SP1683 | sugar ABC transporter, sugar-binding protein |  |  |  | **1.662** |  | **1.04** | **3.005** |  |
| SP1686 | HYPOTHETICAL 41.4 KDA PROTEIN IN LEUX-FECE INTERGENIC REGION. |  |  |  |  | **-2.488** |  |  |  |
| SP1687 | SIALIDASE B PRECURSOR (EC 3.2.1.18) (NEURAMINIDASE B). [3.2.1.18] |  |  |  |  | **-2.812** |  |  |  |
| SP1688 | ABC transporter, permease protein |  | **1.955** |  | **3.98** | **-2.349** | **3.229** | **5.209** | **3.16** |
| SP1689 | ABC transporter, permease protein | **-1.426** |  |  | **4.52** | **-2.925** | **4.043** | **5.564** |  |
| SP1690 | ABC transporter, substrate-binding protein | **-1.4227** |  |  | **4.7** | **-2.58** | **3.865** | **5.071** |  |
| SP1691 | conserved hypothetical protein |  |  |  | **3.048** |  | **2.562** | **3.41** |  |
| SP1693 | neuraminidase A, authentic frameshift |  |  |  | **1.774** | **-1.416** |  | **4.121** |  |
| SP1694 | hypothetical protein |  |  |  | **1.115** |  |  | **2.091** | **2.873** |
| SP1695 | acetyl xylan esterase, putative |  | **2.347** |  | **1.603** |  |  |  | **2.444** |
| SP1703 | conserved domain protein | **-1.1323** |  | **-1.3797** |  |  |  | **1.662** |  |
| SP1704 | ABC transporter, ATP-binding protein | **-1.243** |  | **-1.62** |  |  |  | **1.789** |  |
| SP1705 | hypothetical protein |  |  | **-1.408** |  |  |  | **1.671** |  |
| SP1706 | hypothetical protein |  |  | **-1.0253** |  |  |  | **1.488** |  |
| SP1707 | hypothetical protein | **-1.1153** |  | **-1.03** |  |  |  | **1.425** |  |
| SP1708 | hypothetical protein | **-1.1187** |  |  |  |  |  | **1.355** |  |
| SP1714 | hypothetical protein |  |  |  |  | **-2.426** |  |  |  |
| SP1715 | ABC transporter, ATP-binding protein |  |  | **-1.2787** | **-1.765** | **-2.322** |  |  |  |
| SP1717 | ABC transporter, ATP-binding protein |  |  |  | **1.028** |  |  |  |  |
| SP1719 | IS1239, transposase, putative, degenerate |  |  | **-1.877** | **-1.878** | **-1.698** |  |  |  |
| SP1721 | fructokinase | **-1.669** | **1.5525** | **-1.3327** | **-2.365** | **1.578** | **-2.116** | **-2.193** |  |
| SP1722 | PTS system IIABC components | **-3.325** | **2.2585** | **-2.8577** | **-4.371** | **-1.263** | **-3.102** | **-3.324** |  |
| SP1723 | hypothetical protein | **-1.633** |  |  |  |  |  |  |  |
| SP1724 | sucrose-6-phosphate hydrolase | **-1.8463** | **2.822** | **-1.4547** | **-3.189** | **1.657** | **-3.721** | **-3.777** |  |
| SP1741 | conserved hypothetical protein |  |  |  |  | **1.534** |  |  |  |
| SP1755 | hypothetical protein |  |  |  | **-1.137** |  |  |  |  |
| SP1756 | conserved domain protein |  |  |  | **-1.086** |  |  |  |  |
| SP1758 | glycosyl transferase, group 1 |  |  |  | **-1.108** |  |  |  |  |
| SP1759 | preprotein translocase, SecA subunit |  |  |  | **-1.162** |  |  |  |  |
| SP1760 | conserved domain protein |  |  |  | **-1.090** |  |  |  |  |
| SP1762 | hypothetical protein |  |  |  | **-1.053** |  |  |  |  |
| SP1768 | conserved hypothetical protein |  |  |  | **-1.008** |  |  |  |  |
| SP1770 | glycosyl transferase, family 8 |  |  |  | **-1.202** |  |  |  |  |
| SP1771 | glycosyl transferase, family 2-glycosyl transferase family 8 |  |  |  | **-1.172** |  |  |  |  |
| SP1774 | transcriptional regulator, putative |  |  |  |  | **1.014** |  |  |  |
| SP1788 | hypothetical protein | **-1.214** | **1.6115** |  |  |  |  |  |  |
| SP1791 | integrase-related protein |  | **2.398** | **2.603** |  |  |  |  |  |
| SP1793 | hypothetical protein |  |  | **1.4653** |  |  |  |  |  |
| SP1794 | hypothetical protein |  |  | **1.391** |  | **1.788** |  |  |  |
| SP1795 | sucrose-6-phosphate hydrolase, putative |  |  |  |  | **-1.295** |  |  |  |
| SP1796 | ABC transporter, substrate-binding protein |  |  |  |  | **-1.58** |  |  |  |
| SP1797 | ABC transporter, permease protein |  |  |  |  | **-1.677** |  |  |  |
| SP1798 | ABC transporter, permease protein |  |  |  |  | **-1.288** |  |  |  |
| SP1799 | sugar-binding transcriptional regulator, LacI family |  |  |  |  | **1.609** |  |  |  |
| SP1800 | transcriptional activator, putative |  | **-1.669** |  | **-1.233** | **-1.223** |  |  |  |
| SP1801 | HYPOTHETICAL PROTEIN HOMOLOGOUS TO SPT011133 |  |  |  |  | **-1.045** |  |  |  |
| SP1802 | hypothetical protein |  |  |  |  | **-1.349** |  |  |  |
| SP1803 | conserved hypothetical protein |  |  |  |  | **-1.157** |  |  |  |
| SP1804 | general stress protein 24, putative |  |  |  |  | **-1.306** |  |  |  |
| SP1805 | hypothetical protein |  |  |  |  | **-1.316** |  |  |  |
| SP1806 | conserved domain protein |  |  |  |  |  |  |  |  |
| SP1807 | acetyltransferase, GNAT family |  | **2.0615** |  | **1.019** |  |  |  |  |
| SP1808 | type IV prepilin peptidase, putative | **-1.141** |  |  |  | **-1.803** |  |  |  |
| SP1831 | hypothetical protein |  |  |  | **1.022** | **1.264** |  |  |  |
| SP1832 | hypothetical protein |  |  |  | **1.026** |  |  |  |  |
| SP1836 | HYPOTHETICAL PROTEIN HOMOLOGOUS TO SPT011095 |  |  |  |  | **1.064** |  |  |  |
| SP1847 | XANTHINE PHOSPHORIBOSYLTRANSFERASE (FRAGMENT). |  |  |  |  | **2.546** |  |  |  |
| SP1848 | XANTHINE PERMEASE. |  |  |  |  | **1.919** |  |  |  |
| SP1852 | galactose-1-phosphate uridylyltransferase |  | **2.1745** |  |  |  |  |  | **2.4665** |
| SP1853 | galactokinase |  | **2.216** |  |  |  |  | **1.092** | **1.871** |
| SP1856 | transcriptional regulator, MerR family |  |  |  | **1.032** |  |  |  |  |
| SP1857 | cation efflux system protein |  | **3.958** |  | **2.678** |  | **2.624** | **2.361** |  |
| SP1858 | transcriptional regulator, TetR family |  |  |  |  | **1.09** |  |  |  |
| SP1868 | conserved domain protein |  | **2.2345** |  |  |  |  |  |  |
| SP1869 | iron-compound ABC transporter, permerase protein |  | **-2.0195** |  |  |  |  |  |  |
| SP1871 | iron-compound ABC transporter, ATP-binding protein | **1.09** | **-1.9065** |  |  | **-1.235** |  |  |  |
| SP1884 | PTS system, IIABC components |  | **-1.7505** | **-1.313** | **-4.001** |  |  | **-3.332** |  |
| SP1885 | trehalose operon transcriptional repressor |  |  |  | **-2.039** |  |  | **-1.396** |  |
| SP1892 | hypothetical protein | **-1.48** |  |  | **-1.003** |  |  |  |  |
| SP1894 | GLUCOSYLTRANSFERASE-S (EC 2.4.1.5) (GTF-S) (DEXTRANSUCRASE) (SUCROSE 6-GLUCOSYLTRANSFERASE). [2.4.1.5] |  |  |  |  | **-2.52** |  |  |  |
| SP1895 | sugar ABC transporter, permease protein |  | **1.7635** | **-1.1747** | **-1.501** | **-1.589** |  |  |  |
| SP1896 | MULTIPLE SUGAR-BINDING TRANSPORT SYSTEM PERMEASE PROTEIN MSMF. |  |  |  |  | **-3.203** |  |  |  |
| SP1897 | sugar ABC transporter, sugar-binding protein |  | **3.0175** | **-2.2773** | **-2.734** | **-3.919** |  | **-1.403** | **2.2865** |
| SP1898 | alpha-galactosidase |  | **2.7205** | **-2.432** | **-2.43** | **-3.085** |  | **-1.254** |  |
| SP1899 | msm operon regulatory protein | **-1.2987** |  |  |  |  |  |  |  |
| SP1906 | chaperonin, 60 kDa |  |  |  | **1.128** |  |  |  |  |
| SP1908 | single-strand DNA-binding protein, authentic point mutation | **-1.4513** |  | **-1.326** |  |  |  |  |  |
| SP1912 | hypothetical protein |  |  |  |  | **1.088** |  |  |  |
| SP1913 | cspC-related protein, authentic point mutation |  |  |  | **1.13** |  |  |  |  |
| SP1914 | hypothetical protein |  |  |  | **1.569** |  |  |  |  |
| SP1915 | hypothetical protein |  |  |  | **1.868** |  |  |  |  |
| SP1917 | hypothetical protein |  | **1.8119** |  |  |  |  |  |  |
| SP1919 | HYPOTHETICAL PROTEIN HOMOLOGOUS TO SPT01991 |  |  |  |  | **-1.125** |  |  |  |
| SP1920 | HYPOTHETICAL PROTEIN HOMOLOGOUS TO SPT01990 |  |  |  |  | **-1.29** |  |  |  |
| SP1922 | conserved hypothetical protein |  |  |  | **-1.647** |  |  |  |  |
| SP1923 | pneumolysin |  | **-1.8129** |  | **-3.617** |  |  |  |  |
| SP1924 | hypothetical protein |  | **-1.8061** |  | **-3.815** |  |  |  |  |
| SP1926 | hypothetical protein |  | **-1.7416** |  | **-4.239** |  |  |  |  |
| SP1947 | hypothetical protein |  |  | **1.5023** | **1.798** |  | **1.139** | **1.812** |  |
| SP1948 | conserved domain protein |  |  | **1.696** | **2.618** |  | **1.938** | **2.515** |  |
| SP1949 | hypothetical protein |  |  | **1.5527** | **2.493** |  | **1.691** | **2.284** |  |
| SP1950 | bacteriocin formation protein, putative |  |  |  | **1.126** |  |  | **1.636** |  |
| SP1951 | conserved hypothetical protein |  |  |  |  |  |  | **1.663** |  |
| SP1952 | hypothetical protein |  |  |  |  |  |  | **1.423** |  |
| SP1953 | toxin secretion ABC transporter, ATP-binding-permease protein |  |  |  |  |  |  | **1.322** |  |
| SP1954 | serine protease, subtilase family, authentic frameshift |  |  |  |  |  |  | **1.376** |  |
| SP1955 | hypothetical protein |  |  |  |  |  |  | **1.135** |  |
| SP1956 | hypothetical protein |  |  |  |  |  |  | **1.151** |  |
| SP1957 | ABC transporter, ATP-binding protein |  |  |  |  |  |  | **1.061** |  |
| SP1961 | DNA-directed RNA polymerase, beta subunit |  |  |  | **-1.061** |  |  |  |  |
| SP1962 | hypothetical protein |  |  |  |  | **2.074** |  |  |  |
| SP1971 | hypothetical protein |  |  |  | **1.481** | **-1.252** |  |  |  |
| SP1972 | membrane protein |  |  |  | **1.348** |  |  |  |  |
| SP1977 | hypothetical protein |  | **2.3325** |  |  |  |  |  |  |
| SP1992 | cell wall surface anchor family protein |  |  | **-1.1307** | **-1.658** |  |  |  |  |
| SP1996 | universal stress protein family |  |  |  | **2.916** | **1.012** |  |  |  |
| SP2000 | DNA-binding response regulator |  |  | **-1.317** | **-1.609** | **-2.597** |  |  |  |
| SP2001 | sensor histidine kinase, putative |  |  | **-1.5943** | **-1.86** | **-2.576** |  | **-1.014** |  |
| SP2002 | conserved hypothetical protein |  |  | **-2.6127** | **-3.317** | **-4.129** |  |  |  |
| SP2003 | ABC transporter, ATP-binding protein |  |  | **-2.224** | **-2.921** | **-3.856** |  |  |  |
| SP2004 | hypothetical protein |  |  | **-2.29** | **-2.887** | **-3.846** |  |  |  |
| SP2005 | hypothetical protein |  |  | **-1.792** | **-2.706** | **-3.684** |  |  |  |
| SP2014 | IS630-Spn1, transposase Orf2 |  |  |  | **-1.035** |  |  |  |  |
| SP2021 | glycosyl hydrolase, family 1 |  |  |  | **-1.080** |  |  |  |  |
| SP2022 | PTS system, IIC component |  |  |  | **-1.203** |  |  |  |  |
| SP2024 | PTS system, IIA component |  |  |  | **-1.058** |  |  |  |  |
| SP2026 | alcohol dehydrogenase, iron-containing | **-1.4147** |  | **-1.833** | **2.665** | **-1.275** |  | **1.748** | **2.303** |
| SP2029 | preprotein translocase, YajC subunit |  |  |  |  | **1.124** |  |  |  |
| SP2033 | HYPOTHETICAL PROTEIN HOMOLOGOUS TO SPT01855 |  |  |  |  | **-1.117** |  |  |  |
| SP2038 | PTS system, membrane component, putative |  |  |  |  | **1.559** |  |  |  |
| SP2043 | hypothetical protein |  | **2.258** |  |  |  |  |  |  |
| SP2047 | HYPOTHETICAL PROTEIN HOMOLOGOUS TO SPT01837 |  |  |  |  | **-1.795** |  |  |  |
| SP2048 | conserved hypothetical protein | **-2.794** | **2.9775** | **-1.561** | **1.095** | **-1.455** |  |  |  |
| SP2049 | conserved hypothetical protein | **-2.261** |  |  |  | **-2.748** |  |  |  |
| SP2050 | competence protein CglD | **-1.461** | **2.549** |  |  | **-2.894** |  |  |  |
| SP2051 | Competence protein |  |  |  |  | **-1.655** |  |  |  |
| SP2052 | competence protein CglB | **-2.1507** | **2.5075** | **-2.1215** |  | **-2.108** |  |  |  |
| SP2053 | competence protein CglA | **-1.6987** | **2.065** | **-2.5635** |  | **-2.474** |  |  |  |
| SP2055 | alcohol dehydrogenase, zinc-containing |  |  |  | **3.48** |  | **1.468** | **2.573** |  |
| SP2061 | HYPOTHETICAL 13.3 KDA PROTEIN IN TRPE 5REGION. |  |  |  |  | **-1.541** |  |  |  |
| SP2062 | transcriptional regulator, MarR family |  |  | **-1.8577** |  | **-1.828** |  |  | **1.6279** |
| SP2063 | 42 KDA PROTEIN (ORF1) GENE AND 67 KDA MYOSIN-CROSSREACTIVE STREPTOCOCCAL ANTIGEN (ORF1). |  |  |  |  | **-1.487** |  |  |  |
| SP2064 | hypothetical protein |  |  |  |  | **-1.454** |  |  |  |
| SP2065 | MATE efflux family protein |  |  | **-1.1393** | **-1.172** | **-1.33** |  |  |  |
| SP2066 | threonine synthase |  |  |  | **-1.237** |  |  |  |  |
| SP2067 | hypothetical protein |  |  |  | **-1.682** | **-1.253** |  |  |  |
| SP2072 | glutamine amidotransferase, class-I |  | **2.7025** |  |  |  |  |  |  |
| SP2084 | phosphate ABC transporter, phosphate-binding protein |  | **3.999** | **-1.233** |  |  |  |  |  |
| SP2085 | phosphate ABC transporter, permease protein |  | **3.0135** |  |  |  | **1.089** |  | **2.4555** |
| SP2087 | phosphate ABC transporter, ATP-binding protein |  | **3.5415** |  |  |  |  |  | **2.69** |
| SP2106 | glycogen phosphorylase family protein |  |  |  | **2.449** | **-1.316** |  |  |  |
| SP2107 | 4-ALPHA-GLUCANOTRANSFERASE (EC 2.4.1.25) (AMYLOMALTASE) |  |  |  |  | **-1.568** |  |  |  |
| SP2108 | maltose-maltodextrin ABC transporter, maltose-maltodextrin-binding protein |  | **-1.504** |  |  |  |  | **1.65** |  |
| SP2109 | maltodextrin ABC transporter, permease protein |  |  | **-1.1277** |  | **-1.072** |  | **1.498** |  |
| SP2110 | maltodextrin ABC transporter, permease protein |  |  |  |  |  |  | **1.081** |  |
| SP2127 | transketolase, C-terminal subunit |  |  |  |  |  |  | **2.161** |  |
| SP2129 | PTS system, IIC component, putative |  |  |  |  |  |  | **2.179** |  |
| SP2130 | PTS system, IIB component, putative |  |  |  |  |  |  | **2.25** |  |
| SP2131 | transcriptional regulator, BglG family |  |  |  |  |  |  | **1.603** |  |
| SP2141 | glycosyl hydrolase-related protein |  |  |  | **1.239** |  |  | **1.221** |  |
| SP2142 | ROK family protein |  | **2.543** |  | **1.007** |  |  | **1.758** |  |
| SP2145 | antigen, cell wall surface family protein |  | **1.5525** |  |  |  |  |  |  |
| SP2148 | arginine deiminase | **-1.225** | **4.671** | **-1.3083** | **3.527** | **-2.089** | **1.903** | **5.041** | **2.761** |
| SP2150 | ORNITHINE TRANSCARBAMOYLASE (EC 2.1.3.3) |  |  |  |  | **-1.565** |  |  |  |
| SP2151 | CARBAMATE KINASE (EC 2.7.2.2). [2.7.2.2] |  |  |  |  | **-1.122** |  |  |  |
| SP2152 | conserved hypothetical protein |  | **4.553** |  | **1.495** | **-1.31** |  | **3.833** |  |
| SP2153 | peptidase, M20-M25-M40 family |  | **4.3265** |  | **1.495** | **-1.2006** |  | **3.833** |  |
| SP2157 | alcohol dehydrogenase, iron-containing |  | **2.232** |  | **3.129** | **-1.41** | **1.595** | **2.647** | **3.353** |
| SP2158 | L-fucose isomerase |  |  |  | **1.478** | **-1.072** |  | **1.289** |  |
| SP2159 | fucolectin-related protein |  |  |  | **1.815** | **-1.421** | **1.087** | **1.66** |  |
| SP2161 | Phosphotransferase system sugar-specific EII component |  |  |  |  | **-1.493** |  |  |  |
| SP2162 | Phosphotransferase system sugar-specific EII component |  |  |  |  | **-1.034** |  |  |  |
| SP2163 | Phosphotransferase system sugar-specific EII component |  |  |  |  | **-1.031** |  |  |  |
| SP2164 | PTS system, IIA component |  |  |  | **2.664** | **-1.21** | **1.879** | **2.49** |  |
| SP2165 | Fucose pathway protein, function unknown |  |  |  |  | **-1.734** |  |  |  |
| SP2166 | L-fuculose phosphate aldolase | **-1.1023** |  |  | **2.902** | **-1.396** | **2.158** | **3.136** |  |
| SP2167 | L-fuculose kinase fucK, putative |  |  |  | **2.199** | **-1.443** | **1.33** | **2.312** |  |
| SP2169 | zinc ABC transporter, zinc-binding adhesion liprotein |  |  |  | **-1.168** |  |  |  |  |
| SP2170 | zinc ABC transporter, permease protein |  |  |  | **-1.252** |  |  |  |  |
| SP2171 | zinc ABC transporter, ATP-binding protein |  |  |  | **-1.065** |  |  |  |  |
| SP2172 | adc operon repressor AdcR |  |  |  | **-1.012** |  |  |  |  |
| SP2174 | D-alanyl carrier protein |  |  |  | **1.768** |  |  |  |  |
| SP2176 | D-alanine-activating enzyme |  |  | **1.0176** | **2.038** |  |  |  |  |
| SP2177 | hypothetical protein |  |  | **1.007** | **1.94** | **1.855** |  |  |  |
| SP2180 | conserved hypothetical protein, interruption |  |  | **1.6** |  | **1.557** |  |  |  |
| SP2181 | group II intron, maturase, degenerate |  |  |  |  | **-1.242** |  |  |  |
| SP2182 | hypothetical protein | **-1.079** |  |  | **2.113** |  |  | **2.67** |  |
| SP2183 | hypothetical protein |  | **1.8805** |  | **1.043** |  |  | **3.558** | **2.446** |
| SP2185 | hypothetical protein | **-1.1053** | **1.9175** | **-1.803** | **1.699** | **-1.225** |  | **5.186** |  |
| SP2186 | glycerol kinase |  |  | **-1.7185** | **1.203** | **-1.937** |  | **4.871** | **2.0355** |
| SP2197 | ABC transporter, substrate-binding protein, putative |  | **-3.0545** |  | **-1.695** |  |  | **-1.413** | **-2.665** |
| SP2198 | ABC transporter, permease protein |  | **-2.6755** |  | **-1.633** |  |  | **-1.299** | **-2.197** |
| SP2200 | hypothetical protein | **-1.0147** |  |  |  |  |  |  |  |
| SP2206 | ribosomal subunit interface protein |  |  | **1.8787** | **3.5** |  | **1.287** | **1.099** |  |
| SP2228 | inosine-5-monophosphate dehydrogenase |  |  | **1.295** |  | **1.389** |  |  |  |
| SP2232 | conserved hypothetical protein, authentic frameshift |  | **1.8835** |  |  | **-1.431** |  |  |  |
| SP2233 | hypothetical protein |  | **1.683** |  |  | **-1.078** |  |  |  |
| SP2234_1 | hypothetical protein |  |  |  |  | **1.601** |  |  |  |
| SP2235_1 | RESPONSE REGULATOR COME. |  |  |  |  | **-1.183** |  |  |  |
| SP2236 | putative sensor histidine kinase ComD |  |  |  |  | **-1.242** |  |  |  |
| SP2237 | competence stimulating peptide 2 |  |  |  |  | **-1.275** |  |  |  |
| SP2238_1 | HYPOTHETICAL 18.1 KDA PROTEIN IN COMC 5REGION (ORFL). |  |  |  |  | **1.139** |  |  |  |
| SP2239_1 | HTRA PROTEIN. |  |  |  |  | **-3.055** |  |  |  |
| SP2240 | spspoJ protein |  |  |  | **3.698** | **-2.75** |  |  |  |

**Table S6. Proteins showing differences in abundance in the attenuated sRNA mutants compared to the parental TIGR4**. The differences are calculated from spot percentages (individual spot density divided by total density of all measured spots). Polypeptide spots increased in each Mutant vs T4 WT by a fold increase of > 1.7 and p value < 0.05 or a fold increase of > 3.0 are highlighted in blue in the difference column, while spots decreased in each Mutant vs T4 WT with a fold decrease of ≤ -1.7 and p value < 0.05 or a fold decrease of < -3.0 are highlighted in red. All differences were visually verified using the montage images found on the following pages. A total of 684 spots were analyzed. Values are fold change from TIGR4. Proteomic analysis of spots from 2D gel analysis (as per Supplementary Figure 6) yielded pI and molecular weigth (MW).

|  |  |  | **∆F7** | **∆F20** | **∆F22** | **∆F25** | **∆F32** | **∆F41** | **∆F44** | **∆F48** |
| --- | --- | --- | --- | --- | --- | --- | --- | --- | --- | --- |
| **Spot #** | **pI** | **MW** | **Difference** | **Difference** | **Difference** | **Difference** | **Difference** | **Difference** | **Difference** | **Difference** |
| 66 | 6.4 | 80,484 | **1.9** | 1.1 | 1.5 | 1.2 | **2.3** | 1.1 | 1.2 | 1.5 |
| 340 | 6.4 | 39,081 | **1.7** | 1.6 | 1.3 | 1.0 | -1.0 | -1.0 | 1.1 | 1.4 |
| 625 | 6.6 | 17,863 | **3.0** | -1.2 | 1.4 | -1.0 | -1.2 | 1.0 | 1.7 | 1.3 |
| 640 | 5.3 | 16,635 | **5.2** | **17.0** | 1.2 | 1.7 | 1.6 | 1.7 | 1.5 | -1.1 |
| 651 | 5.8 | 15,283 | **1.7** | -1.4 | -1.0 | 1.2 | -1.6 | 2.3 | 1.5 | 1.5 |
| 653 | 5.3 | 15,177 | **4.6** | 1.2 | 1.3 | 2.0 | 1.2 | 1.6 | 1.7 | 1.9 |
| 680 | 5.3 | 11,404 | **2.4** | 1.8 | 1.6 | 2.1 | 1.1 | 1.9 | 2.6 | 2.4 |
| 56 | 5.8 | 82,928 | **-3.0** | 1.1 | -1.7 | -1.7 | **-5.2** | 1.1 | -1.0 | **-8.1** |
| 102 | 5.4 | 71,687 | **-3.4** | 1.3 | -2.1 | **-4.1** | **-4.6** | -1.9 | **-5.6** | **-4.6** |
| 107 | 6.1 | 71,014 | **-2.1** | -1.1 | -1.3 | 1.1 | **-3.1** | 1.1 | -1.6 | -1.1 |
| 167 | 5.0 | 58,096 | **-8.4** | **-10.6** | **-5.7** | **-5.2** | **-4.2** | **-7.6** | **-4.2** | **-6.1** |
| 200 | 5.1 | 51,432 | **-6.3** | -2.4 | **-3.3** | -2.5 | **-4.9** | **-4.0** | -2.3 | **-4.8** |
| 302 | 5.1 | 41,351 | **-8.2** | **-6.5** | **-5.7** | **-3.4** | **-7.3** | **-4.1** | **-10.9** | **-8.0** |
| 314 | 5.8 | 40,910 | **-3.4** | 1.4 | -1.9 | -2.4 | -1.0 | -1.8 | -1.1 | -2.0 |
| 337 | 5.6 | 39,214 | **-2.9** | -2.0 | **-1.7** | -1.7 | 2.4 | **-3.0** | **-2.4** | **-3.3** |
| 522 | 5.5 | 27,663 | **-1.9** | -1.1 | -1.9 | 1.0 | -1.2 | -1.7 | **-1.7** | -1.3 |
| 667 | 5.5 | 13,131 | **-2.0** | 1.0 | -1.5 | -1.5 | -1.0 | -1.9 | -1.6 | -1.6 |
| 50 | 5.9 | 84,684 | 1.7 | **3.2** | 2.5 | 2.1 | **3.2** | 1.4 | 1.6 | 1.8 |
| 92 | 5.6 | 75,200 | -1.1 | **2.0** | 1.0 | 1.1 | 1.1 | 1.7 | 1.2 | -1.1 |
| 124 | 5.9 | 67,296 | -1.5 | **2.7** | -1.3 | -1.0 | 1.4 | -1.1 | -1.7 | -1.3 |
| 137 | 5.6 | 65,012 | -1.5 | **3.7** | -1.6 | -2.0 | -2.8 | -1.1 | -2.3 | -1.1 |
| 150 | 5.5 | 62,553 | 1.4 | **2.5** | 1.2 | 1.3 | -1.2 | 2.2 | 1.4 | 1.2 |
| 195 | 5.9 | 52,557 | 1.1 | **3.9** | 1.1 | 1.6 | -1.0 | 2.1 | -1.0 | 1.2 |
| 197 | 5.8 | 52,284 | 1.1 | **3.0** | -1.3 | 1.2 | -1.2 | 1.2 | -1.0 | 1.1 |
| 213 | 6.4 | 50,102 | 1.3 | **2.2** | 1.6 | 1.2 | 1.1 | 1.2 | 1.1 | 1.1 |
| 214 | 5.9 | 49,966 | 1.3 | **2.2** | -1.4 | 1.3 | **1.8** | 1.1 | 1.4 | 1.3 |
| 234 | 5.1 | 46,808 | -1.9 | **1.7** | -1.1 | -1.3 | -1.2 | **-2.1** | -1.1 | 1.3 |
| 253 | 7.0 | 44,920 | 1.1 | **1.8** | 1.3 | 1.3 | 1.3 | 1.2 | 1.5 | 1.0 |
| 309 | 5.9 | 41,168 | -1.3 | **3.0** | -1.3 | **-2.4** | -1.4 | -1.6 | -1.8 | **-2.0** |
| 328 | 6.7 | 39,943 | 1.3 | **2.8** | 1.4 | 1.6 | 1.6 | -1.1 | 1.3 | 1.8 |
| 341 | 5.1 | 38,749 | 1.2 | **1.7** | -1.1 | 1.2 | **1.8** | -1.1 | -1.0 | -1.0 |
| 344 | 5.7 | 38,854 | -1.1 | **2.0** | -1.3 | -1.2 | 1.1 | -1.1 | -1.1 | -1.4 |
| 350 | 6.3 | 38,777 | 1.3 | **1.8** | 1.2 | 1.1 | **1.8** | 1.1 | 1.4 | 1.3 |
| 352 | 5.6 | 38,597 | 1.1 | **3.6** | -1.2 | -1.0 | 1.0 | -1.1 | -1.1 | -1.0 |
| 358 | 6.2 | 38,219 | -1.0 | **5.4** | 1.2 | -1.5 | 1.7 | -1.5 | 1.3 | 1.1 |
| 364 | 6.7 | 37,813 | 1.2 | **2.4** | 1.2 | 1.2 | -1.0 | -1.0 | 1.0 | 1.3 |
| 366 | 5.2 | 37,414 | 1.0 | **2.2** | 1.1 | 1.3 | 1.0 | -1.3 | -1.4 | 1.3 |
| 372 | 6.0 | 37,103 | 1.0 | **2.7** | 1.3 | 1.1 | -1.0 | 1.2 | -1.2 | -1.2 |
| 394 | 6.7 | 35,429 | 1.1 | **2.0** | 1.1 | 1.2 | -1.5 | 1.0 | 1.2 | 1.4 |
| 406 | 6.2 | 34,618 | -1.2 | **2.9** | -1.0 | -1.2 | -1.2 | -1.6 | 1.0 | 1.0 |
| 453 | 5.6 | 31,552 | -1.3 | **2.1** | 1.1 | -1.1 | **2.4** | -1.2 | 1.1 | -1.4 |
| 482 | 5.5 | 29,804 | 1.1 | **1.9** | 1.0 | 1.3 | -1.2 | -1.4 | 1.1 | 1.2 |
| 511 | 5.7 | 28,268 | 1.2 | **2.2** | 1.1 | 1.5 | -1.1 | -1.0 | 1.2 | 1.0 |
| 548 | 5.6 | 26,311 | -1.0 | **2.0** | 1.2 | 1.2 | 1.3 | 1.1 | 1.2 | 1.2 |
| 559 | 6.2 | 25,795 | 1.2 | **1.8** | 1.2 | 1.1 | **-2.5** | 1.2 | 1.1 | 1.1 |
| 606 | 6.0 | 20,663 | -1.0 | **6.4** | 1.0 | -1.2 | -1.3 | 2.2 | 2.3 | -1.2 |
| 621 | 5.9 | 18,734 | 1.7 | **29.5** | -1.0 | 1.0 | **4.6** | 1.9 | 1.8 | 2.0 |
| 628 | 5.2 | 17,809 | 1.7 | **4.0** | 1.3 | 1.4 | 1.2 | 1.2 | 1.3 | 1.6 |
| 656 | 5.7 | 14,501 | -1.8 | **2.6** | -1.7 | -1.6 | -1.6 | -1.4 | -1.3 | -1.8 |
| 663 | 6.0 | 13,638 | -1.0 | **1.7** | -1.0 | 1.0 | **-2.0** | -1.0 | 1.1 | 1.1 |
| 7 | 5.7 | 169,249 | -1.0 | **-10.2** | -1.0 | -1.2 | -1.9 | 1.3 | -1.7 | -1.2 |
| 10 | 5.5 | 156,125 | -1.2 | **-8.3** | -1.1 | -1.2 | **-3.0** | 1.1 | -1.7 | -1.5 |
| 11 | 5.7 | 156,125 | 1.3 | **-4.3** | -2.0 | -1.3 | -1.3 | -1.4 | -1.1 | 2.3 |
| 19 | 5.6 | 132,502 | 1.3 | **-3.4** | 1.1 | 1.0 | -1.5 | 1.0 | 1.1 | 2.1 |
| 22 | 6.7 | 117,565 | 1.1 | **-5.6** | **1.7** | -1.1 | **3.1** | -1.0 | 1.6 | -2.1 |
| 23 | 6.7 | 115,860 | 1.3 | **-12.0** | 1.3 | 1.3 | **2.4** | 1.1 | 1.9 | -1.6 |
| 24 | 6.7 | 115,007 | 1.3 | **-9.8** | 1.3 | 1.3 | 1.2 | 1.2 | 1.6 | -1.8 |
| 27 | 6.8 | 112,449 | 1.2 | **-3.7** | 1.1 | 1.4 | -2.6 | 1.2 | 1.3 | -1.5 |
| 62 | 5.2 | 81,295 | 1.0 | **-4.7** | 1.1 | -1.1 | -2.2 | -2.0 | -1.6 | -1.2 |
| 78 | 7.0 | 78,340 | 1.1 | **-6.2** | 1.9 | 1.5 | -1.8 | 1.1 | 2.6 | -1.2 |
| 139 | 5.6 | 63,783 | -1.3 | **-3.4** | 1.4 | 1.0 | -1.0 | 1.6 | 1.3 | 1.9 |
| 140 | 5.7 | 63,607 | -2.2 | **-3.9** | -1.9 | -2.3 | -1.6 | -2.4 | -1.4 | -2.9 |
| 159 | 6.6 | 61,565 | -1.4 | **-3.1** | 1.0 | 1.1 | -1.3 | **-2.0** | **-3.4** | -1.4 |
| 162 | 6.1 | 59,102 | -1.1 | **-1.7** | 1.0 | 1.2 | 1.3 | -1.0 | 1.1 | 1.0 |
| 199 | 6.9 | 51,875 | -1.4 | **-4.6** | -1.5 | -1.1 | 1.1 | -1.6 | -2.4 | -1.2 |
| 227 | 5.6 | 48,329 | -1.4 | **-3.4** | -1.4 | -1.2 | -1.6 | -1.3 | -2.1 | **-3.9** |
| 232 | 5.2 | 47,784 | -1.5 | **-2.0** | -1.3 | 1.2 | **4.6** | -1.2 | 1.2 | 1.1 |
| 256 | 5.3 | 44,511 | -1.4 | **-2.4** | -1.4 | 1.0 | 1.1 | -1.1 | 1.0 | -1.0 |
| 268 | 6.5 | 43,284 | 1.2 | **-1.8** | 1.5 | 1.4 | **-3.3** | 1.2 | 1.0 | 1.4 |
| 275 | 5.7 | 42,607 | -1.7 | **-9.0** | -1.4 | -1.7 | 1.2 | -2.0 | -1.3 | -2.6 |
| 276 | 5.4 | 42,453 | -1.5 | **-6.3** | -1.2 | -1.1 | -1.1 | -1.6 | -1.1 | 1.0 |
| 279 | 5.3 | 42,299 | -1.6 | **-8.7** | -1.2 | -1.2 | 1.0 | -2.1 | -1.4 | 1.2 |
| 287 | 6.4 | 42,276 | 1.2 | **-1.8** | 1.1 | -1.3 | -1.1 | -1.3 | 1.1 | 1.2 |
| 292 | 5.4 | 41,990 | -1.1 | **-6.7** | -1.1 | 1.1 | 1.6 | -1.1 | -1.2 | -1.4 |
| 296 | 5.7 | 41,785 | -1.2 | **-4.4** | -1.1 | -1.1 | -1.7 | -1.6 | 1.3 | -1.4 |
| 319 | 6.8 | 40,450 | 1.4 | **-2.5** | 1.7 | 1.6 | **-4.3** | 1.2 | -1.0 | 1.4 |
| 324 | 6.0 | 43,973 | 1.0 | **-2.3** | 1.0 | **-1.7** | -1.1 | 1.1 | -1.2 | -1.4 |
| 326 | 5.8 | 39,985 | -1.1 | **-8.1** | 1.1 | -1.8 | -2.3 | 1.1 | 1.0 | -1.0 |
| 329 | 5.1 | 39,598 | -1.7 | **-7.2** | 1.1 | -1.1 | **2.9** | -1.1 | 1.2 | -2.0 |
| 330 | 6.6 | 39,791 | 1.5 | **-7.0** | -1.0 | 1.7 | **-2.6** | 1.0 | 1.2 | 1.2 |
| 335 | 5.0 | 39,067 | -1.4 | **-3.4** | -1.4 | -1.8 | -1.5 | -2.5 | -1.1 | -1.3 |
| 347 | 5.3 | 38,751 | -1.3 | **-13.4** | -1.1 | -1.0 | **-2.9** | -1.3 | -1.0 | -1.0 |
| 349 | 5.4 | 38,699 | -2.7 | **-5.4** | -1.6 | -1.1 | **-7.1** | -2.1 | -1.6 | -1.5 |
| 359 | 5.6 | 37,980 | -1.1 | **-6.0** | -1.1 | -1.0 | **-4.9** | -1.8 | -2.4 | -1.1 |
| 368 | 6.3 | 37,255 | -2.9 | **-15.1** | -2.2 | **-4.2** | **-7.2** | -2.3 | **-5.1** | -2.4 |
| 380 | 7.2 | 36,454 | -1.5 | **-8.7** | -1.7 | -1.6 | 1.0 | -1.5 | **-3.8** | -1.4 |
| 405 | 6.4 | 34,669 | -1.1 | **-3.3** | 1.3 | 1.1 | -2.5 | 1.0 | 1.6 | 1.4 |
| 457 | 5.9 | 31,090 | -1.1 | **-3.7** | -1.1 | 1.2 | -1.2 | 1.2 | 1.6 | -1.1 |
| 462 | 5.6 | 30,832 | -1.1 | **-2.6** | -1.2 | 1.2 | 1.7 | 1.1 | 1.1 | 1.1 |
| 496 | 7.2 | 28,730 | -1.2 | **-12.7** | -1.4 | -1.3 | -1.2 | -1.7 | -1.3 | 1.2 |
| 497 | 5.6 | 30,252 | 1.0 | **-11.1** | 1.5 | 1.4 | -1.4 | 1.1 | 1.4 | -1.1 |
| 498 | 5.7 | 30,252 | -1.5 | **-4.4** | -1.3 | 1.1 | -2.8 | -1.1 | -1.2 | -1.5 |
| 499 | 5.6 | 30,171 | -1.1 | **-23.7** | -1.2 | 1.1 | **-2.2** | -1.1 | 1.1 | -1.2 |
| 516 | 7.2 | 27,835 | 1.1 | **-2.8** | -1.1 | -1.3 | -1.3 | -1.1 | -1.0 | -1.1 |
| 549 | 5.0 | 25,894 | 1.2 | **-2.2** | 1.1 | 1.0 | 1.1 | -1.1 | 1.0 | 1.0 |
| 561 | 5.8 | 25,351 | -1.1 | **-6.1** | 1.1 | -1.1 | -2.0 | 1.0 | -1.2 | -1.1 |
| 585 | 6.1 | 23,529 | 1.0 | **-3.3** | 1.2 | 1.0 | -1.3 | 1.1 | -1.0 | 1.0 |
| 596 | 5.8 | 21,936 | 1.0 | **-2.2** | 1.0 | -1.0 | **-2.3** | -1.3 | -1.2 | -1.0 |
| 599 | 5.8 | 21,616 | -1.1 | **-2.6** | 1.2 | -1.1 | **-2.8** | -1.4 | -1.1 | -1.1 |
| 611 | 6.3 | 20,093 | 1.3 | **-1.8** | 1.3 | 1.0 | -1.1 | -1.1 | **2.2** | **2.0** |
| 620 | 5.8 | 18,770 | 1.2 | **-14.7** | 1.4 | -1.3 | **-6.1** | -1.2 | -1.2 | -1.0 |
| 662 | 6.1 | 14,030 | 1.1 | **-2.1** | 1.2 | -1.1 | **-2.1** | -1.1 | -1.0 | 1.1 |
| 49 | 6.0 | 85,375 | 2.2 | 2.7 | **3.1** | 2.1 | **5.0** | 2.4 | **2.4** | 1.4 |
| 205 | 6.2 | 51,466 | 1.3 | 1.2 | **2.0** | 1.3 | 1.1 | -1.1 | 1.1 | 1.3 |
| 429 | 6.8 | 33,350 | 1.2 | -1.8 | **1.7** | -1.1 | **2.0** | 1.2 | 1.4 | 1.1 |
| 435 | 6.3 | 32,894 | 1.4 | -1.5 | **2.2** | 1.4 | **2.1** | 1.3 | 1.5 | -1.1 |
| 449 | 6.3 | 32,032 | -1.1 | -1.4 | **2.3** | 1.3 | 2.0 | -1.2 | 1.0 | -1.2 |
| 474 | 6.8 | 30,764 | 1.2 | 1.5 | **1.7** | 1.1 | 1.2 | 1.2 | 1.1 | 1.2 |
| 480 | 6.7 | 30,155 | 1.3 | 2.3 | **1.7** | -1.0 | 1.7 | -1.2 | 2.0 | 2.4 |
| 485 | 6.5 | 29,749 | 1.0 | 1.1 | **2.5** | -1.2 | **1.8** | -1.4 | 1.3 | 1.2 |
| 487 | 6.3 | 29,547 | 1.5 | 1.0 | **3.4** | 1.1 | 2.4 | 1.0 | 2.1 | 1.2 |
| 488 | 6.2 | 29,445 | 1.3 | 1.2 | **2.6** | 1.1 | **3.3** | -1.1 | -1.0 | -1.2 |
| 495 | 6.4 | 30,339 | 1.0 | 1.3 | **1.8** | -1.3 | **3.3** | -1.3 | 1.1 | 1.1 |
| 507 | 6.4 | 28,573 | 1.1 | 1.1 | **2.1** | 1.2 | **1.8** | -1.0 | 1.3 | -1.0 |
| 169 | 5.3 | 58,148 | -2.5 | -2.3 | **-3.4** | -2.3 | **-5.2** | **-3.5** | -2.7 | **-4.1** |
| 221 | 5.4 | 49,011 | -2.9 | 1.1 | **-3.0** | -2.5 | -1.6 | -2.3 | -1.8 | -2.6 |
| 12 | 4.9 | 151,352 | 2.0 | 1.8 | 2.2 | **1.9** | 1.7 | 1.3 | 1.4 | 1.8 |
| 207 | 6.7 | 51,057 | 1.3 | 1.1 | 1.2 | **1.7** | -1.1 | 1.1 | 1.1 | 1.3 |
| 304 | 6.7 | 41,515 | 1.2 | -1.2 | -1.3 | **2.0** | 1.1 | 1.2 | 1.4 | 1.4 |
| 3 | 5.6 | 183,247 | -1.7 | -2.3 | -1.6 | **-3.5** | -2.5 | **-3.0** | -1.8 | -2.2 |
| 323 | 5.9 | 40,293 | -1.2 | -1.5 | -1.2 | **-1.8** | -1.1 | 1.0 | **-1.8** | **-1.8** |
| 327 | 5.6 | 39,933 | -1.4 | -1.5 | -1.5 | **-2.8** | -1.3 | -1.8 | -2.5 | -1.6 |
| 4 | 7.1 | 178,962 | 1.8 | -1.2 | 1.1 | 1.1 | **3.0** | 1.6 | 1.5 | -1.2 |
| 6 | 6.1 | 172,987 | 1.5 | 1.0 | 2.5 | 1.1 | **3.7** | -1.1 | 1.7 | 1.4 |
| 8 | 6.3 | 168,724 | 1.5 | -1.1 | 1.5 | 1.3 | **4.5** | 1.2 | 1.9 | 1.1 |
| 17 | 5.9 | 135,127 | -1.2 | -1.3 | -1.1 | 1.0 | **3.9** | -1.1 | 1.0 | 1.3 |
| 37 | 5.3 | 88,373 | 1.4 | 2.1 | 1.7 | 1.6 | **5.0** | 1.2 | 2.2 | 2.7 |
| 59 | 7.0 | 82,293 | 1.6 | 1.1 | 1.5 | 1.4 | **2.0** | 1.4 | 1.8 | 1.2 |
| 72 | 6.7 | 78,698 | -1.3 | -1.4 | 1.6 | 1.8 | **6.0** | 1.2 | 2.3 | -1.2 |
| 73 | 6.8 | 78,698 | -1.7 | 1.1 | -1.0 | 1.1 | **3.5** | -1.5 | 1.8 | 1.1 |
| 77 | 7.1 | 78,243 | -1.2 | 1.0 | -1.2 | -1.0 | **5.2** | 2.0 | 1.7 | 2.1 |
| 111 | 7.0 | 68,853 | 1.5 | 1.3 | 1.4 | 1.7 | **2.2** | **1.9** | **2.2** | 2.1 |
| 130 | 7.0 | 65,908 | -1.1 | -1.4 | 1.2 | -1.5 | **3.2** | -1.6 | 1.7 | 1.3 |
| 202 | 5.8 | 51,602 | 1.1 | 1.4 | -1.3 | 1.2 | **2.1** | 1.2 | 1.2 | 1.1 |
| 222 | 5.7 | 49,011 | 1.4 | 1.6 | 1.1 | 1.4 | **1.7** | 1.5 | 1.3 | 1.3 |
| 241 | 6.0 | 46,420 | -1.6 | 1.4 | 1.3 | 1.3 | **1.8** | -1.0 | 1.1 | **1.8** |
| 278 | 6.5 | 42,428 | 1.8 | 1.5 | 1.5 | 1.3 | **2.0** | 1.2 | 1.2 | 1.1 |
| 307 | 6.0 | 45,419 | -1.0 | -1.1 | 1.4 | 1.0 | **4.0** | 1.0 | -1.5 | -2.0 |
| 308 | 6.1 | 41,262 | 1.1 | 1.0 | 1.3 | -1.1 | **1.8** | 1.3 | 1.0 | -1.1 |
| 334 | 5.7 | 39,316 | -1.4 | -1.6 | -1.2 | -1.3 | **1.7** | **-1.9** | 1.0 | -1.6 |
| 339 | 5.2 | 38,802 | -1.5 | -2.1 | -1.1 | -1.3 | **4.1** | -1.2 | 1.2 | 1.1 |
| 342 | 6.9 | 39,030 | 1.1 | -1.1 | 1.4 | 1.2 | **2.1** | 1.0 | 1.2 | 1.4 |
| 353 | 5.2 | 38,545 | 1.0 | 1.2 | -1.2 | 1.1 | **1.8** | -1.0 | 1.3 | 1.1 |
| 356 | 5.8 | 38,494 | -1.3 | -1.2 | -1.1 | -1.1 | **1.7** | 1.0 | 1.2 | -1.3 |
| 362 | 5.4 | 37,774 | 1.2 | 1.7 | 1.2 | 1.4 | **4.0** | -1.1 | 1.3 | 1.4 |
| 407 | 5.3 | 34,380 | 1.3 | -1.7 | 1.7 | 1.2 | **3.1** | 1.2 | 1.1 | 1.5 |
| 409 | 6.5 | 34,466 | -1.2 | -1.7 | 1.3 | -1.1 | **2.1** | -1.2 | 1.1 | -1.1 |
| 420 | 7.0 | 33,338 | 1.8 | 1.4 | 1.7 | 1.7 | **2.0** | 1.1 | 1.4 | 1.4 |
| 432 | 6.1 | 33,097 | 1.4 | -1.4 | 1.6 | 1.5 | **2.4** | 1.8 | 1.8 | 1.0 |
| 446 | 6.7 | 32,133 | 1.4 | 1.5 | 2.1 | 1.2 | **2.1** | 1.3 | 1.6 | 1.6 |
| 450 | 6.9 | 32,032 | 1.3 | 1.3 | 1.5 | 1.1 | **1.7** | 1.4 | 1.2 | 1.5 |
| 454 | 5.7 | 31,552 | 1.1 | -1.0 | -1.2 | 1.6 | **6.5** | 1.5 | **2.2** | 1.2 |
| 473 | 5.0 | 30,091 | 1.4 | 1.3 | 1.4 | 1.3 | **1.9** | 1.2 | 1.4 | 1.3 |
| 500 | 6.7 | 30,131 | 1.2 | 1.4 | 1.6 | -1.1 | **2.3** | -1.0 | 1.1 | -1.0 |
| 537 | 6.2 | 26,928 | 1.3 | 2.2 | 1.3 | 1.0 | **1.8** | -1.1 | 1.6 | **2.1** |
| 551 | 5.2 | 25,824 | 1.2 | 1.2 | 1.7 | 1.6 | **1.7** | 1.5 | 1.5 | 1.8 |
| 571 | 6.1 | 24,735 | 1.0 | -1.0 | 1.4 | -1.2 | **1.7** | 1.1 | 1.5 | 1.6 |
| 26 | 6.9 | 113,302 | 1.6 | -1.7 | 1.3 | 1.6 | **-4.2** | 1.6 | 1.5 | 1.0 |
| 85 | 5.4 | 77,659 | -2.9 | -1.5 | -1.6 | -2.5 | **-3.1** | -2.6 | -2.3 | -1.4 |
| 142 | 6.0 | 63,432 | 1.1 | 1.3 | -1.1 | -1.1 | **-1.8** | 1.1 | -1.2 | -1.1 |
| 171 | 6.4 | 57,466 | -1.3 | -1.2 | 1.9 | -1.1 | **-2.0** | 1.1 | 1.3 | 1.7 |
| 229 | 5.9 | 48,057 | -1.1 | -1.9 | -1.1 | 1.1 | **-3.7** | 1.0 | 1.2 | -2.1 |
| 264 | 6.1 | 43,693 | -1.7 | 1.3 | -1.0 | -1.7 | **-2.4** | -1.2 | -1.5 | -1.1 |
| 266 | 6.2 | 43,420 | -1.1 | 1.3 | 1.2 | -1.6 | **-1.8** | -2.2 | **-1.9** | 1.2 |
| 298 | 5.6 | 41,630 | -1.5 | -1.7 | 1.3 | -1.4 | **-3.8** | -1.7 | -1.1 | 1.2 |
| 299 | 6.9 | 41,667 | 1.0 | -1.1 | -1.2 | 1.2 | **-1.7** | 1.0 | -1.1 | 1.2 |
| 322 | 6.9 | 40,400 | 1.3 | -1.1 | 1.2 | 1.7 | **-3.3** | 1.1 | -1.1 | 1.7 |
| 370 | 5.7 | 37,105 | -1.3 | -1.4 | 1.1 | 1.1 | **-1.7** | **-2.1** | 1.3 | -1.3 |
| 422 | 6.2 | 33,654 | -1.2 | -1.0 | 1.1 | 1.3 | **-3.4** | -1.2 | -1.1 | 1.4 |
| 528 | 6.4 | 27,440 | 1.2 | -1.2 | 1.5 | 1.2 | **-3.0** | 1.3 | 1.1 | 1.1 |
| 568 | 6.2 | 24,808 | 1.1 | 1.0 | 1.3 | -1.1 | **-1.9** | -1.1 | -1.0 | 1.1 |
| 577 | 5.8 | 24,141 | -1.1 | 1.1 | 1.0 | -1.7 | **-4.1** | -1.1 | -1.3 | -1.2 |
| 581 | 7.0 | 23,648 | 1.3 | 1.3 | 1.7 | -1.0 | **-3.6** | 1.3 | 1.4 | -1.0 |
| 590 | 6.9 | 22,980 | 1.2 | 1.3 | 1.3 | -1.2 | **-3.2** | 1.3 | -1.1 | 1.0 |
| 592 | 7.0 | 22,615 | 1.7 | -1.1 | 1.4 | 1.1 | **-6.4** | 1.4 | -1.4 | 1.3 |
| 597 | 5.9 | 21,687 | 1.2 | -1.8 | 1.5 | 1.1 | **-1.9** | 1.3 | 1.1 | 1.3 |
| 623 | 5.5 | 18,343 | -1.0 | 1.6 | -1.1 | -1.2 | **-3.2** | -1.3 | -1.0 | 1.3 |
| 629 | 5.1 | 17,748 | -1.0 | 1.3 | 1.2 | 1.1 | **-2.0** | 1.0 | 1.2 | 1.1 |
| 635 | 6.1 | 16,730 | -1.1 | 1.9 | 1.0 | -1.1 | **-1.9** | -1.0 | 1.4 | 1.3 |
| 644 | 6.3 | 15,743 | 1.5 | -1.5 | 1.3 | 1.2 | **-1.9** | 1.5 | 1.3 | 1.2 |
| 664 | 6.5 | 13,366 | 1.3 | -1.3 | 1.1 | -1.1 | **-2.3** | 1.2 | 1.1 | 1.0 |
| 666 | 6.3 | 13,195 | 1.3 | -2.0 | 1.0 | 1.0 | **-2.8** | 1.3 | 1.2 | 1.4 |
| 35 | 6.1 | 90,848 | 1.2 | 1.4 | 1.3 | 1.4 | 1.9 | **1.7** | 1.2 | 1.2 |
| 176 | 7.0 | 56,051 | -2.0 | -1.8 | -2.3 | -2.8 | 1.1 | **-4.3** | -2.1 | -1.9 |
| 284 | 5.5 | 42,247 | -1.6 | 2.5 | -1.2 | -1.1 | 1.1 | **-2.3** | -1.3 | 1.0 |
| 306 | 5.3 | 41,270 | 1.2 | 1.6 | 1.2 | -1.0 | 1.7 | **-5.4** | -1.2 | 2.7 |
| 396 | 5.7 | 35,203 | -1.5 | 1.2 | -1.0 | 1.2 | -1.4 | **-2.0** | -1.3 | -1.4 |
| 419 | 6.4 | 33,807 | -2.3 | -1.1 | 1.1 | -2.6 | 1.3 | **-3.2** | -1.3 | 1.5 |
| 466 | 6.7 | 30,916 | 1.1 | -1.4 | 1.4 | 1.0 | 1.1 | 1.0 | **2.2** | 2.5 |
| 676 | 6.1 | 11,929 | 1.8 | -2.0 | -1.1 | 1.5 | -2.1 | 1.8 | **2.6** | 2.6 |
| 127 | 6.4 | 67,083 | -1.0 | -1.8 | -1.3 | 1.4 | -1.6 | -1.1 | **-2.0** | 1.1 |
| 68 | 5.4 | 79,766 | 1.2 | -1.1 | -1.1 | 1.3 | 1.5 | 1.6 | 1.4 | **1.8** |
| 108 | 5.1 | 69,126 | 1.3 | -1.3 | 1.2 | -1.0 | 1.5 | 1.0 | -1.1 | **2.1** |
| 112 | 4.9 | 68,768 | 1.3 | -1.1 | 1.1 | 1.2 | 1.1 | 1.2 | 1.6 | **1.9** |
| 132 | 5.1 | 65,547 | 2.1 | -1.3 | 1.6 | 1.1 | -1.3 | -1.0 | 1.9 | **3.0** |
| 204 | 5.1 | 51,160 | 1.1 | -1.3 | 1.2 | -1.1 | -1.0 | -1.3 | 1.4 | **2.0** |
| 240 | 6.0 | 46,420 | -1.6 | -1.0 | 1.2 | 1.4 | 1.2 | -1.7 | 1.0 | **1.9** |
| 365 | 7.0 | 37,441 | 1.3 | 1.3 | 1.3 | 1.2 | 1.9 | -1.0 | 1.3 | **1.7** |
| 553 | 5.1 | 25,755 | 1.2 | 1.2 | -1.0 | 1.5 | 1.1 | -1.1 | 2.0 | **2.4** |
| 613 | 5.6 | 19,872 | 1.4 | -1.6 | 1.5 | -1.1 | -1.7 | 1.1 | 1.7 | **2.1** |
| 136 | 5.4 | 65,012 | -1.7 | -1.9 | -2.2 | -2.8 | 1.2 | -1.5 | -2.2 | **-3.0** |
| 376 | 6.0 | 36,797 | -1.1 | -2.3 | 1.2 | -1.1 | -1.2 | 1.2 | -1.1 | **-2.7** |
| 433 | 6.5 | 33,097 | 1.1 | -1.6 | 1.5 | -1.0 | -1.1 | 1.3 | -1.0 | **-4.1** |

**Figure S1. Northern blots of sRNAs identified by RNA-seq.** Probes specific for the respective sRNAs were generated (Supplementary Table 2) and used to query for the presence of signal. Samples marked by a * indicate the knockout strain being run adjacent to the parental TIGR4.


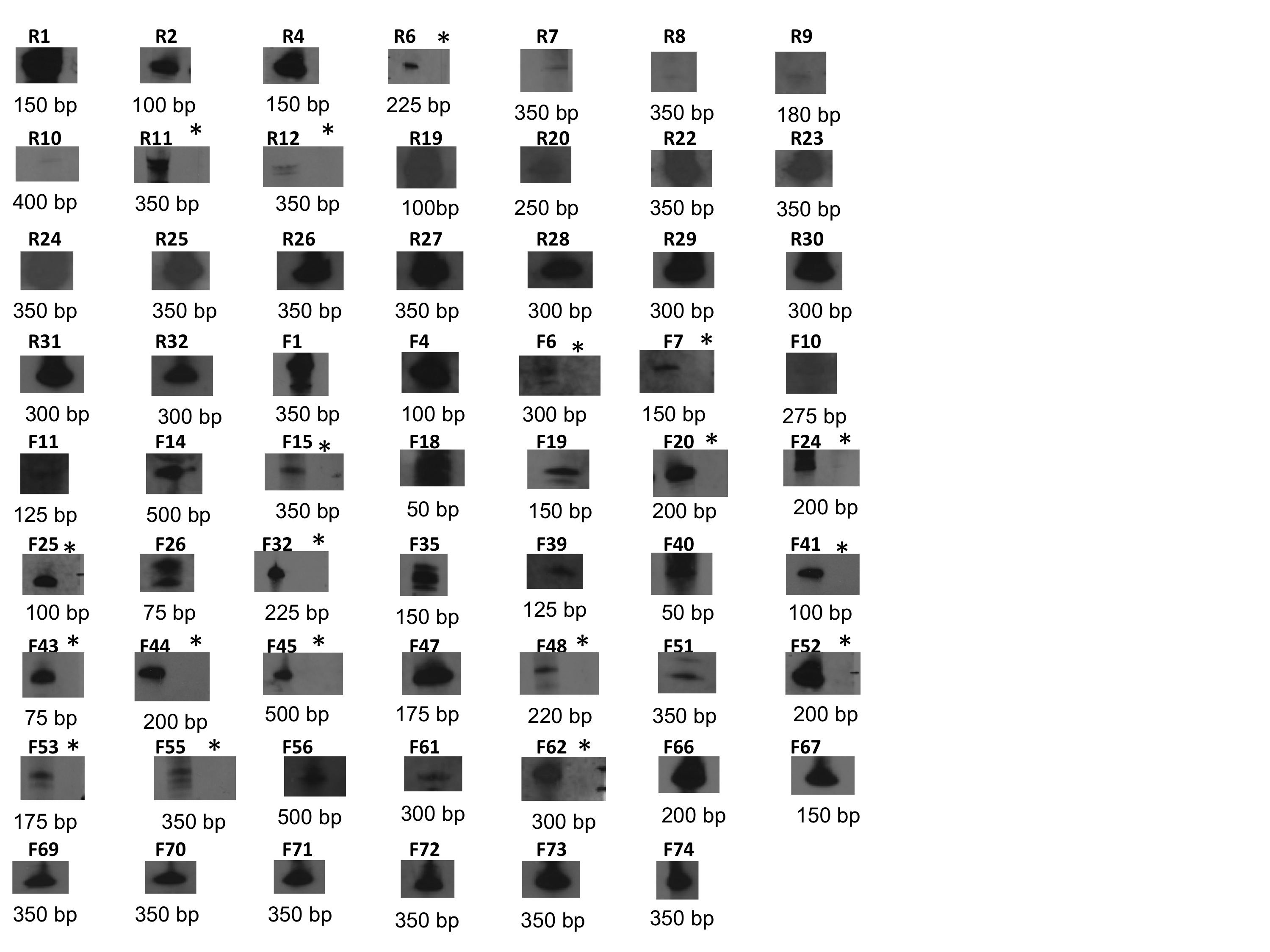


**Figure S2. Differential expression of sRNA F13 in TCS response regulator knockouts.** The graph shows sequence coverage for the F13 intergenic region flanked by two ORFs (red bars; SP493 and SP494). Height of the gray line corresponds to sequence coverage. Each strain was assayed at least 3 times. T4R= TIGR4 parental strain.


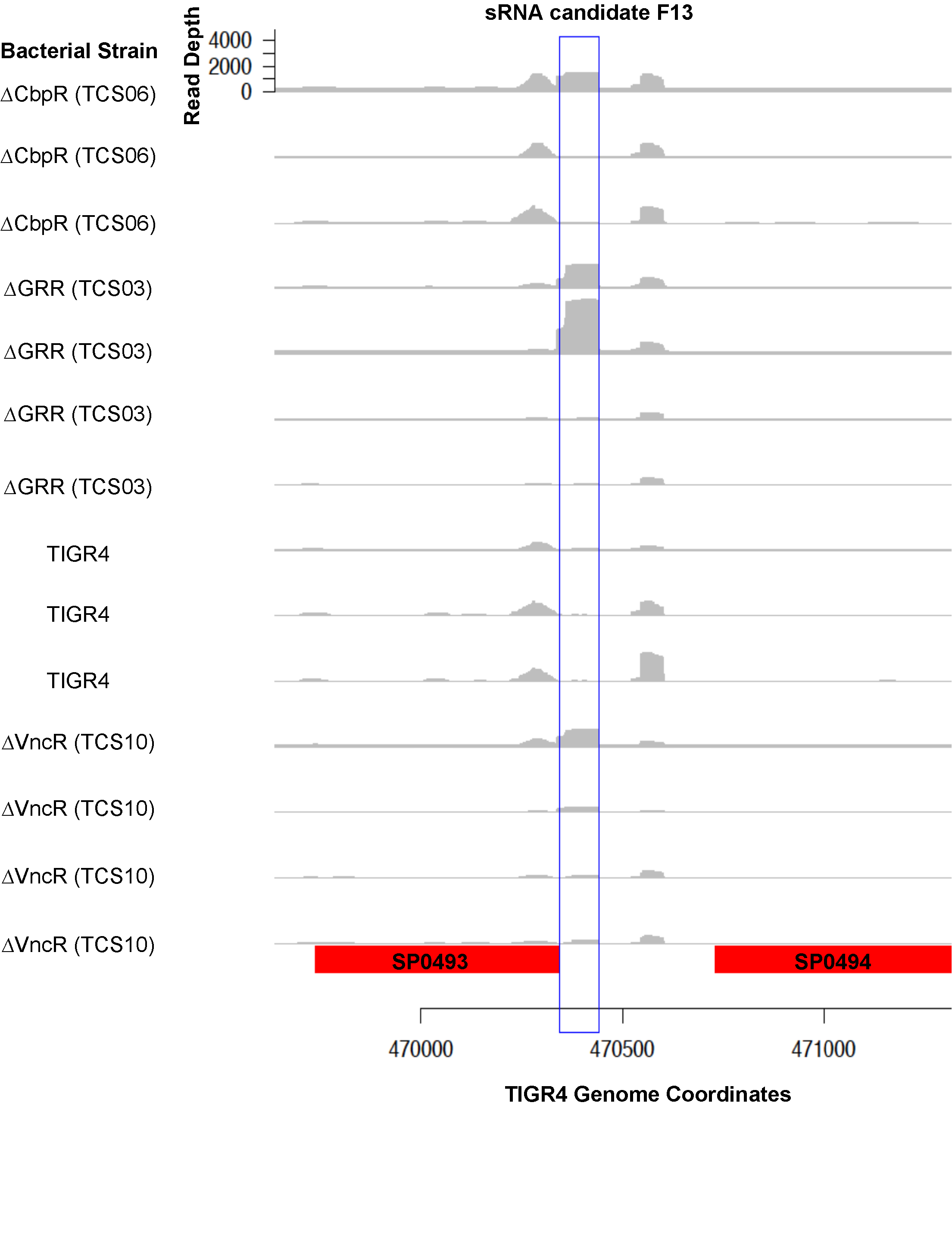


**Figure S3.** Motifs overrepresented in the sRNA sequences identified using the MEME software to identify motifs. The sRNA that the motif was found within is indicated (Name), along with the position within the RNA (Start), the strand encoding the motif (+ or -), the p-value, and the consensus of the various motifs (large letters above list of individual sequences; taller letters indicate greater conservation).


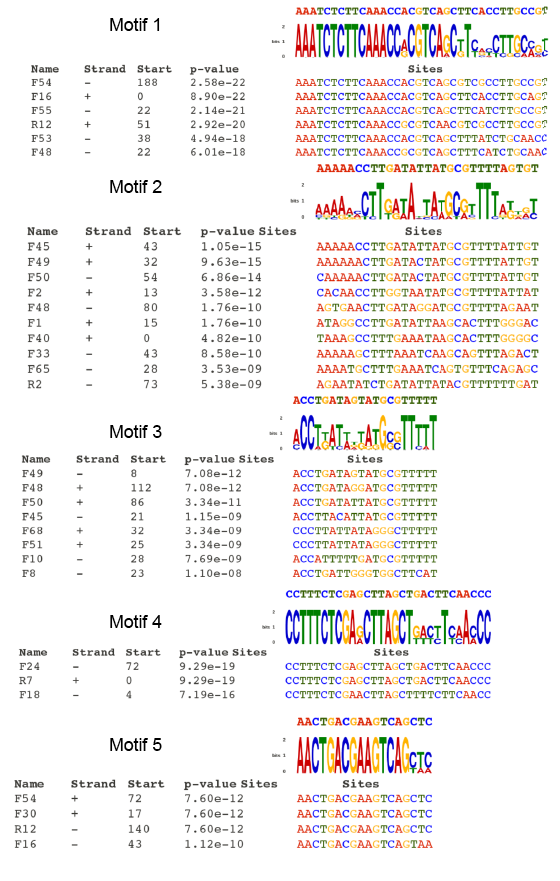


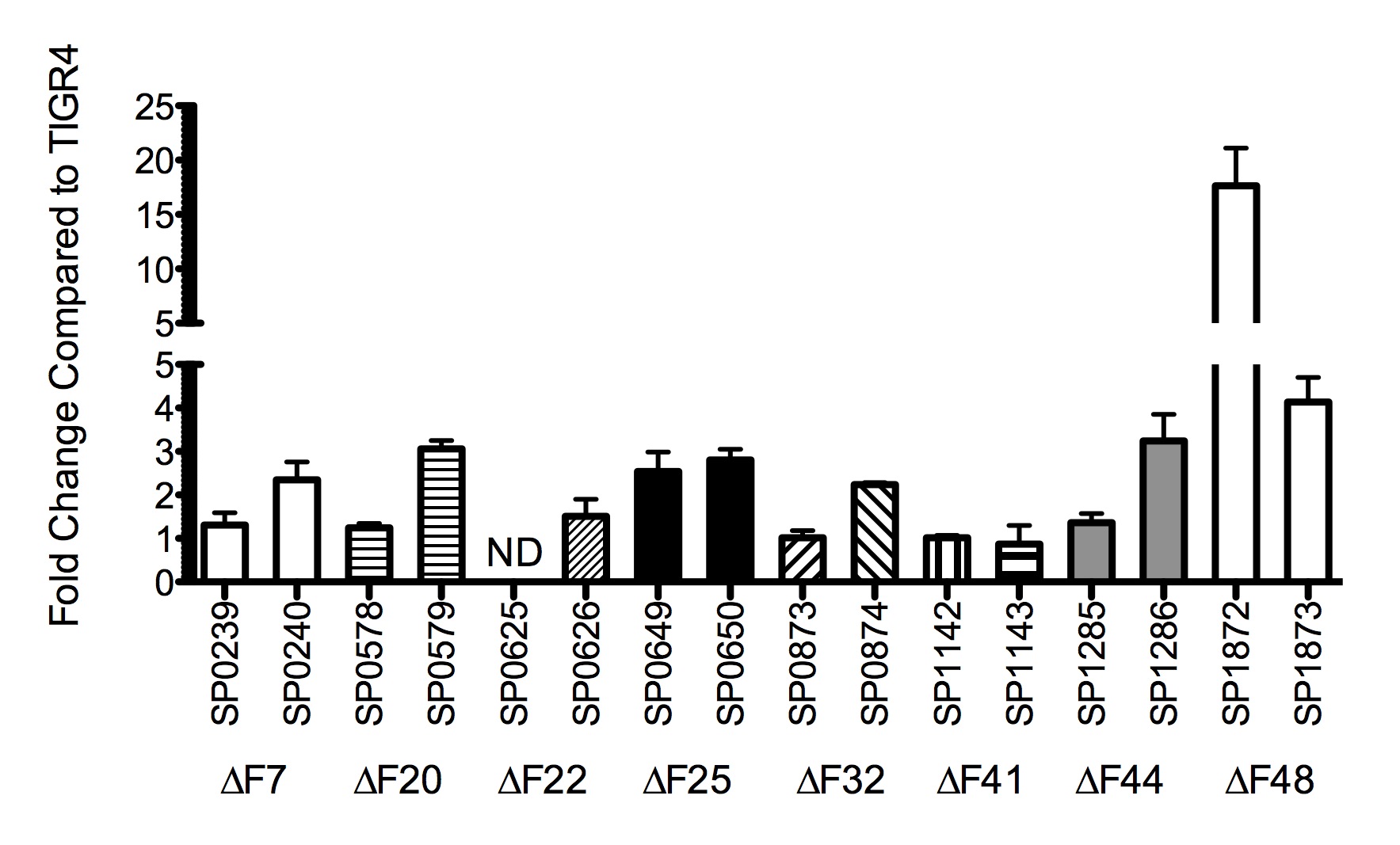


**Figure S4. qRT-PCR analysis of the genes flanking the sRNA knockouts.** All values are given relative to the abundance of transcript observed in TIGR4. Data presents the mean and standard deviation from 3 biological replicates. ND= not detected. Each sRNA is shown below its two flanking genes (pair of bars sharing fill pattern).

**Figure S5. Adhesion and invasion of TIGR4R and the attenuated sRNA mutants to activated nasopharyngeal (Detroit) and endothelial (RBCEC6) cells.** Data represents mean and standard deviation from at least 3 independent experiments. TIGR4R values were set at 100%; absolute values in cfu/well were 5x10^5 and 4x10^4 for nasopharyngeal adhesion and invasion respectively (A); and 1x10^5 and 2x10^3 CFUs per well for endothelial adhesion and invasion (B) respectively.


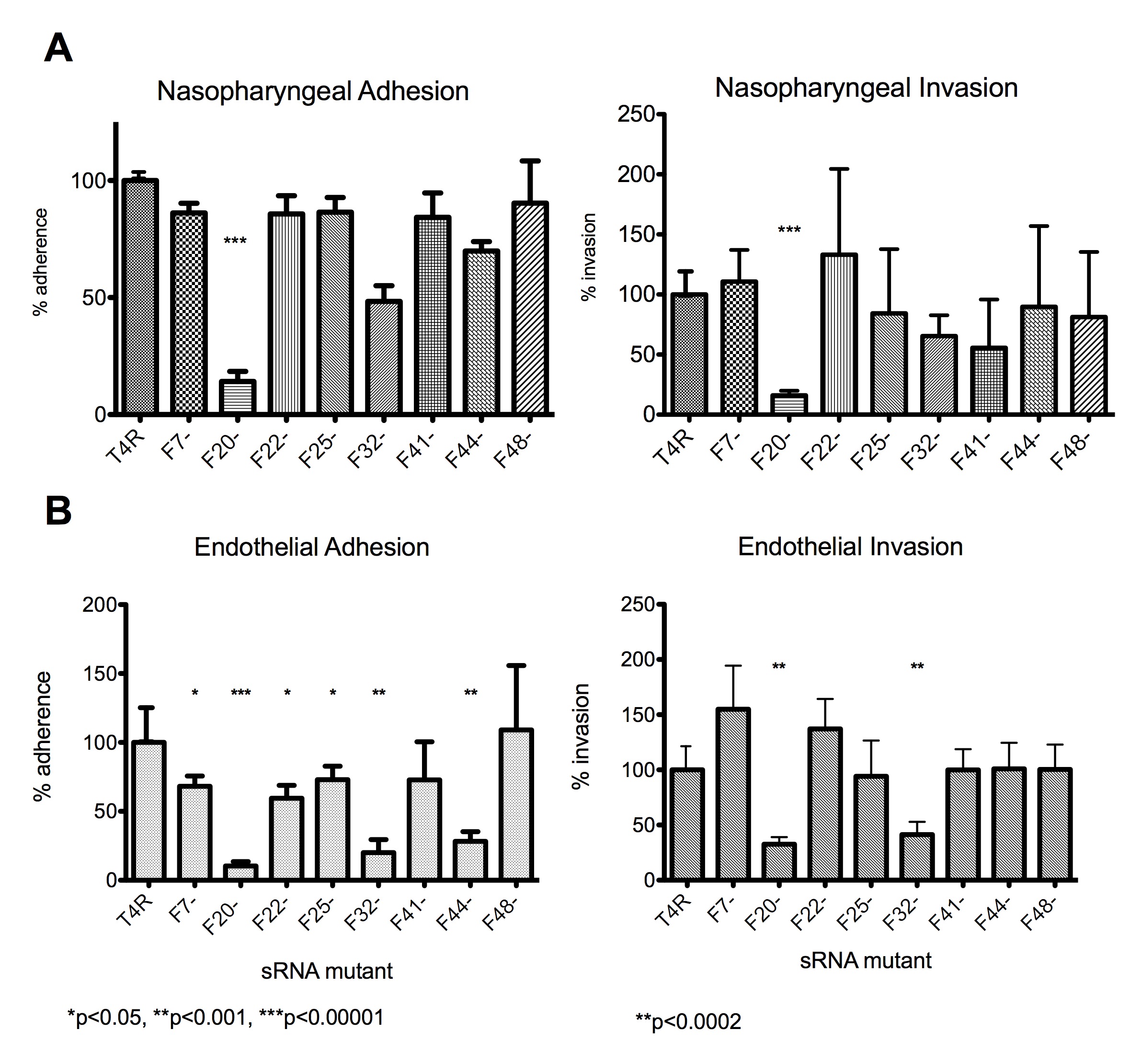


**Figure S6.** Representative 2D gel utilized for the proteomic analysis of TIGR4 with the individual spots being identified by circles. Molecular weight markers are shown on far right.


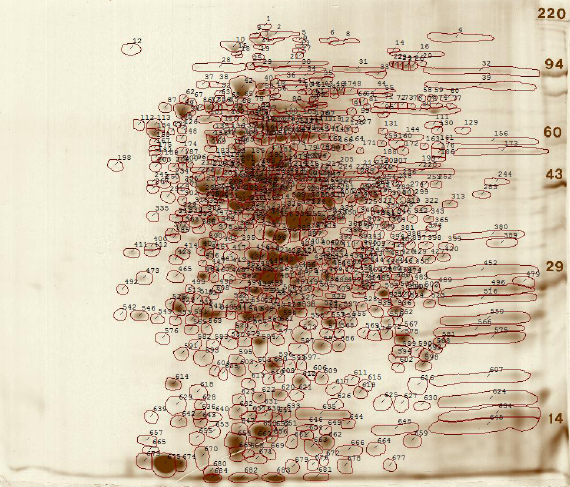

Supplement: Text S1 — Supplementary Materials. This file includes the following: Supplementary Methods; Strains used in this study (Table S1), Primers used in this study (Table S2); Coordinates of the additional regions of the TIGR4 genome containing the conserved motifs identified in Figure S4 (Table S3); Raw data from the Tn-seq screen (Table S4); Microarray analysis of attenuated sRNA mutants (Table S5); Proteins showing differences in abundance in the attenuated sRNA mutants compared to the parental TIGR4 (Table S6); Northern blots of sRNAs identified by RNA-seq (Figure S1); Differential expression of sRNA F13 in TCS response regulator knockouts (Figure S2); Motifs overrepresented in the sRNA sequences identified using the MEME software to identify motifs (Figure S3); qRT-PCR analysis of the genes flanking the sRNA knockouts (Figure S4); Adhesion and invasion of TIGR4R and the attenuated sRNA mutants to activated nasopharyngeal (Detroit) and endothelial (RBCEC6) cells (Figure S5); Representative 2D gel utilized for the proteomic analysis of TIGR4 with the individual spots being identified by circles (Figure S6). (DOC) [file ppat.1002788.s001.doc]
